# Senkyunolide I Inhibits mtDNA‐cGAS‐STING Signaling in Macrophages via Targeting VDAC1 Oligomerization to Attenuate Ulcerative Colitis

**DOI:** 10.1002/advs.77045

**Published:** 2026-08-07

**Authors:** Zhiming Ye, Yihang Huang, Bohao Han, Lei Zhang, Can Yu, Yang Yang, Bing Liu, Yongping Jian, Zhixiang Xu, Cheng Zeng

**Affiliations:** ^1^ Center for Drug Research and Development Guangdong Pharmaceutical University Guangzhou Guangdong China; ^2^ Key Laboratory of Pathobiology Ministry of Education Jilin University Changchun Jilin China; ^3^ Department of Hepatobiliary‐Pancreatic & Hernia Surgery The Affiliated Guangdong Second Provincial General Hospital of Jinan University Guangzhou Guangdong China; ^4^ Department of Pharmacy Zhuhai People's Hospital (Zhuhai Hospital Affiliated With Jinan University) Zhuhai China; ^5^ College of Pharmacy Guangdong Pharmaceutical University Guangzhou China; ^6^ School of Life Sciences Henan University Kaifeng Henan China; ^7^ Key Specialty of Clinical Pharmacy The First Affiliated Hospital of Guangdong Pharmaceutical University Guangzhou Guangdong China

**Keywords:** cGAS‐STING, senkyunolide I, ulcerative colitis, VDAC1 oligomerization

## Abstract

Ulcerative colitis (UC) is a chronic inflammatory bowel disease with limited therapeutic options. The cyclic GMP‐AMP synthase (cGAS)‐stimulator of interferon genes (STING) pathway, activated by cytosolic mitochondrial DNA (mtDNA), has been increasingly implicated in UC. Through proteomics and artificial intelligence modeling, this study identified for the first time that senkyunolide I (SEI), a primary bioactive phthalide from *Ligusticum striatum* DC. (*L. striatum*), inhibits experimental colitis via the cGAS‐STING pathway, with mechanistic validation performed in both intestinal tissues and cultured macrophages. Mutagenesis, activity‐based protein profiling, and micro‐scale thermophoresis reveal that SEI directly binds voltage‐dependent anion channel 1 (VDAC1) at residue K12 to inhibit its stress‐induced oligomerization. This blockade prevents mtDNA release into the cytosol, thereby suppressing the cGAS‐STING cascade and subsequent M1 macrophage polarization, as well as downstream NLRP3 inflammasome activation, pyroptosis, and ferroptosis in macrophages and colon tissues. Overexpression of VDAC1‐WT and VDAC1‐K12A in mice confirms this mechanism. Clinically, VDAC1 expression in UC patients positively correlates with cGAS‐STING activation and disease severity. Collectively, SEI alleviates colitis by targeting VDAC1 oligomerization to inhibit the cGAS‐STING pathway in macrophages, establishing the VDAC1‐cGAS‐STING axis as a promising therapeutic strategy for UC.

## Graphical abstract

In macrophages, senkyunolide I (SEI) directly targets the K12 residue of VDAC1 to inhibit its stress‐induced oligomerization, a critical upstream event that effectively prevents mitochondrial DNA release and subsequent cGAS‐STING pathway activation. This suppresses NLRP3 inflammasome‐mediated pyroptosis, ferroptosis, and oxidative stress, thereby restoring intestinal barrier integrity and alleviating ulcerative colitis.

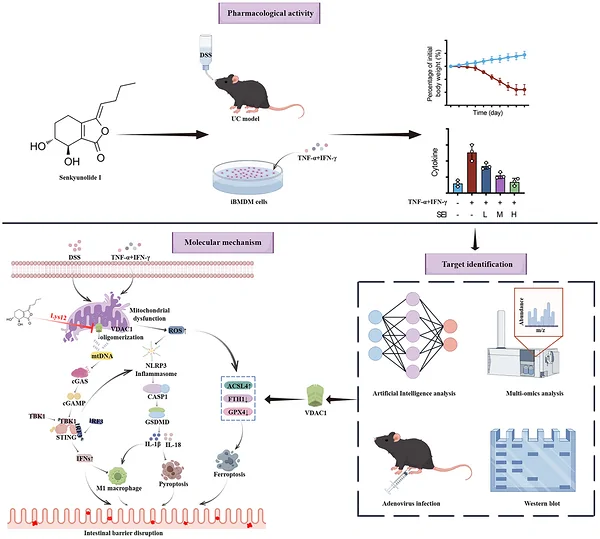

**Abbreviations**
AAV: adeno‐associated virus
AB‐PAS: Alcian Blue‐Periodic Acid‐Schiff
ABPP: activity‐based protein profiling
ACSL4: acyl‐CoA synthetase long chain family member 4
AI: artificial intelligence
ATP: adenosine 5'‐triphosphate
CAT: catalase
CASP1: cysteine‐aspartic acid protease 1
CCL5: C–C motif chemokine ligand 5
CETSA: cellular thermal shift assay
cGAMP: cyclic GMP‐AMP
cGAS: cyclic GMP‐AMP synthase
CD86: cluster of differentiation 86
CXCL10:  C–X–C motif chemokine ligand 10
DAI: disease activity index
DARTS: drug affinity responsive target stability
DCCM: dynamic cross‐correlation matrix
D‐Loop: displacement loop
DSS: dextran sulfate sodium
dsDNA: double‐stranded DNA
ELISA: enzyme‐linked immunosorbent assay
FTH1: ferritin heavy chain 1
GO: gene ontology
GPX: glutathione peroxidase
GPX4: glutathione peroxidase 4
GSH: glutathione
GSDMD: gasdermin D
H&E: Hematoxylin and Eosin
IBD: inflammatory bowel disease
IF: immunofluorescence
IFIT1: interferon‐induced protein with tetratricopeptide repeats 1
IFN‐γ: interferon‐gamma
IFN‐I: type I interferon
IL‐1β: interleukin‐1 beta
IL‐6: interleukin‐6
IL‐23: interleukin‐23
iBMDM: immortalized bone marrow‐derived macrophages
iNOS: inducible nitric oxide synthase
IRF3: interferon regulatory factor 3
ISG15: interferon‐stimulated gene 15 ubiquitin‐like modifier
KEGG: Kyoto Encyclopedia of Genes and Genomes
LPS: lipopolysaccharides
MDA: malondialdehyde
MST: micro‐scale thermophoresis
mt‐cytB: cytochrome b
mt‐ND1: NADH‐ubiquinone oxidoreductase chain 1
mt‐ND2: NADH‐ubiquinone oxidoreductase chain 2
mtDNA: mitochondrial DNA
MUC2: mucin 2
mPTP: mitochondrial permeability transition pore
NLRP3: NLR family Pyrin domain containing 3
PCA: principal component analysis
qRT‐PCR: quantitative real‐time PCR
RMSD: root‐mean‐square deviation
RMSF: root‐mean‐square fluctuation
ROS: reactive oxygen species
SEI: senkyunolide I
SOD: superoxide dismutase
STING: stimulator of interferon genes
TBK1: TANK binding kinase 1
TEM: transmission electron microscopy
TFAMtranscription factor A: transcription factor Amitochondrial
TFF3: trefoil factor 3
Tomm20: mitochondrial import receptor subunit TOM20 homolog
UC: ulcerative colitis
VDAC1: voltage‐dependent anion channel 1
WB: Western blot
WT: wild‐type
ZO‐1: zonula occludens‐1
5‐ASA: 5‐aminosalicylic acid

## 1 Introduction

The cyclic GMP‐AMP synthase (cGAS)‐stimulator of interferon genes (STING) pathway is an evolutionarily conserved DNA‐sensing machinery [1, 2, 3]. It orchestrates innate immune responses by detecting cytosolic double‐stranded DNA (dsDNA) from pathogens or damaged organelles. Upon binding to cyclic GMP‐AMP (cGAMP), the second messenger synthesized by cGAS, STING undergoes oligomerization and translocates from the endoplasmic reticulum to the Golgi apparatus. In the Golgi, it recruits TANK‐binding kinase 1 (TBK1) and interferon regulatory factor 3 (IRF3), initiating type I interferon (IFN‐I) and pro‐inflammatory cytokine production. This pathway is crucial for antiviral defense. However, aberrant STING activation is increasingly recognized as a driver of chronic inflammation in autoimmune diseases, including systemic lupus erythematosus, Aicardi‐Goutières syndrome, and inflammatory bowel disease (IBD) [4, 5]. In ulcerative colitis (UC), a severe IBD subtype, persistent mucosal inflammation arises from dysregulated innate immune signaling [6]. However, the specific molecular mechanisms by which STING dysregulation drives intestinal inflammation in UC, particularly its upstream triggers, remain poorly defined.

Recent studies indicate that macrophages are central regulators in the pathogenesis of UC [7, 8, 9]. In these studies, macrophages adopt a pro‐inflammatory phenotype marked by excessive cytokine secretion and tissue‐damaging reactive oxygen species (ROS) [10, 11]. Under cellular stress, the extracellular release of mitochondrial DNA (mtDNA) constitutes a pivotal pro‐inflammatory event [12, 13]. The core mechanism for this event is mitochondrial membrane permeabilization. This process begins with the opening of the mitochondrial inner membrane permeability transition pore, leading to mitochondrial matrix swelling, outer membrane disruption, and subsequent mtDNA release from the matrix into the intermembrane space and cytoplasm. Importantly, voltage‐dependent anion channel 1 (VDAC1) on the mitochondrial outer membrane oligomerizes in response to stress, forming large‐pore channels that allow mtDNA passage and provide another crucial pathway for its cytoplasmic release [14, 15]. Ultimately, cytoplasmic mtDNA activates the cGAS‐STING signaling pathway. This perpetuates a vicious cycle of inflammation and exacerbates disease by triggering NLRP3 inflammasome activation and ferroptosis [12, 16]. These processes are closely associated with intestinal epithelial barrier disruption.

Natural compounds derived from medicinal plants are increasingly recognized as valuable resources for drug discovery [17]. *L. striatum* (Chuanxiong), a well‐known traditional Chinese medicine herb, exerts potent anti‐inflammatory, antioxidant, and anti‐thrombotic effects [18, 19]. As a core medicinal herb, Chuanxiong is widely incorporated into multiple classic traditional Chinese medicine formulas, such as Quyushengxin formula [20, 21] and Renshen Baidu Powder [22], both of which have demonstrated clinical efficacy in the management of UC in China. The major bioactive constituents of Chuanxiong include phthalide‐rich volatile oils, alkaloids, and phenolic acids [23]. Senkyunolide I (SEI), a predominant phthalide derivative, stands out as a particularly promising candidate due to its exceptional abundance, chemical stability, favorable pharmacokinetic profile, and potent anti‐inflammatory efficacy. As a characteristic marker component of dried Chuanxiong rhizomes, SEI reaches a concentration of approximately 10 mg/g, ranking it among the three most abundant phthalides alongside ligustilide and senkyunolide A [24]. In stark contrast to ligustilide, which readily undergoes *cis–trans* isomerization and oxidative degradation under ambient conditions, SEI exhibits exceptional chemical stability [25]. Furthermore, SEI possesses favorable amphiphilicity, which enables it to rapidly enter the circulation and efficiently penetrate biological membranes [24]. Importantly, its oral bioavailability reaches 37.25%, nearly fivefold higher than the 8% bioavailability of senkyunolide A, with efficient gastrointestinal absorption and rapid clearance following intravenous injection [26, 27]. Accumulating preclinical studies have demonstrated that SEI exerts significant protective effects against hepatic ischemia‐reperfusion injury, thoracic aortic aneurysms and dissections, hepatic steatosis, cecal ligation and puncture‐induced pulmonary injury, and allergic rhinitis [28, 29, 30, 31], with these beneficial effects mediated by suppressing apoptosis, alleviating oxidative damage, and inhibiting key pro‐inflammatory signaling pathways. These pharmacological actions are highly relevant to the pathogenesis of UC, a disease characterized by dysregulated immune responses, epithelial barrier disruption, and excessive mucosal oxidative stress [32]. Although recent evidence suggests protective effects of SEI in functional constipation [33], its specific therapeutic potential and mechanisms of action in UC require further exploration.

In this study, we integrated multi‐omics profiling, artificial intelligence (AI) prediction, and orthogonal experimental validation to investigate the therapeutic potential and mechanism of SEI in ulcerative colitis. We demonstrate that SEI effectively ameliorates experimental colitis by directly targeting VDAC1. Specifically, SEI binds to the K12 residue of VDAC1, inhibits its stress‐induced oligomerization, and consequently blocks the mitochondrial DNA‐cytosolic escape and the subsequent activation of the cGAS‐STING‐NLRP3 inflammatory axis. Furthermore, SEI alleviates oxidative stress and suppresses associated pyroptosis and ferroptosis. Our work not only identifies SEI as a novel VDAC1 inhibitor with therapeutic promise for UC but also establishes the VDAC1‐cGAS‐STING signaling axis as a druggable pathway for the treatment of this disease.

## 2 Results

### 2.1 SEI Alleviates Disease Symptoms in Dextran Sulfate Sodium (DSS)‐Induced Colitis Mice

The DSS‐induced experimental colitis model effectively mimics the pathological features of human UC, making it a valuable tool for investigating disease pathogenesis and developing novel therapeutic agents [34]. To evaluate the effects of SEI on pathological changes, we established DSS‐induced acute and chronic UC models (Figure 1A,B). As shown in Figure 1C–J, mice in the DSS‐treated group exhibited typical UC manifestations, including progressive weight loss, increased disease activity index (DAI) scores, colon shortening, elevated splenic index, and prominent inflammatory cell infiltration (predominantly neutrophils) in the colonic mucosa. In contrast, treatment with graded doses of SEI (12.5, 25, 50 mg/kg/day) or 5‐ASA (100 mg/kg/day) markedly alleviated DSS‐induced pathological manifestations, as reflected by attenuated body weight loss (Figure 1C), lowered DAI scores (Figure 1D), restored colon length (Figure 1E,F), normalized elevated splenic index (Figure 1G), reduced neutrophil infiltration, and ameliorated mucosal ulceration and inflammatory cell accumulation in colonic tissues (Figure 1H–J). Similarly, these protective effects were also observed in DSS‐induced chronic colitis models. These results confirm that SEI ameliorates UC pathological symptoms in a significant dose‐dependent manner.

**FIGURE 1:**
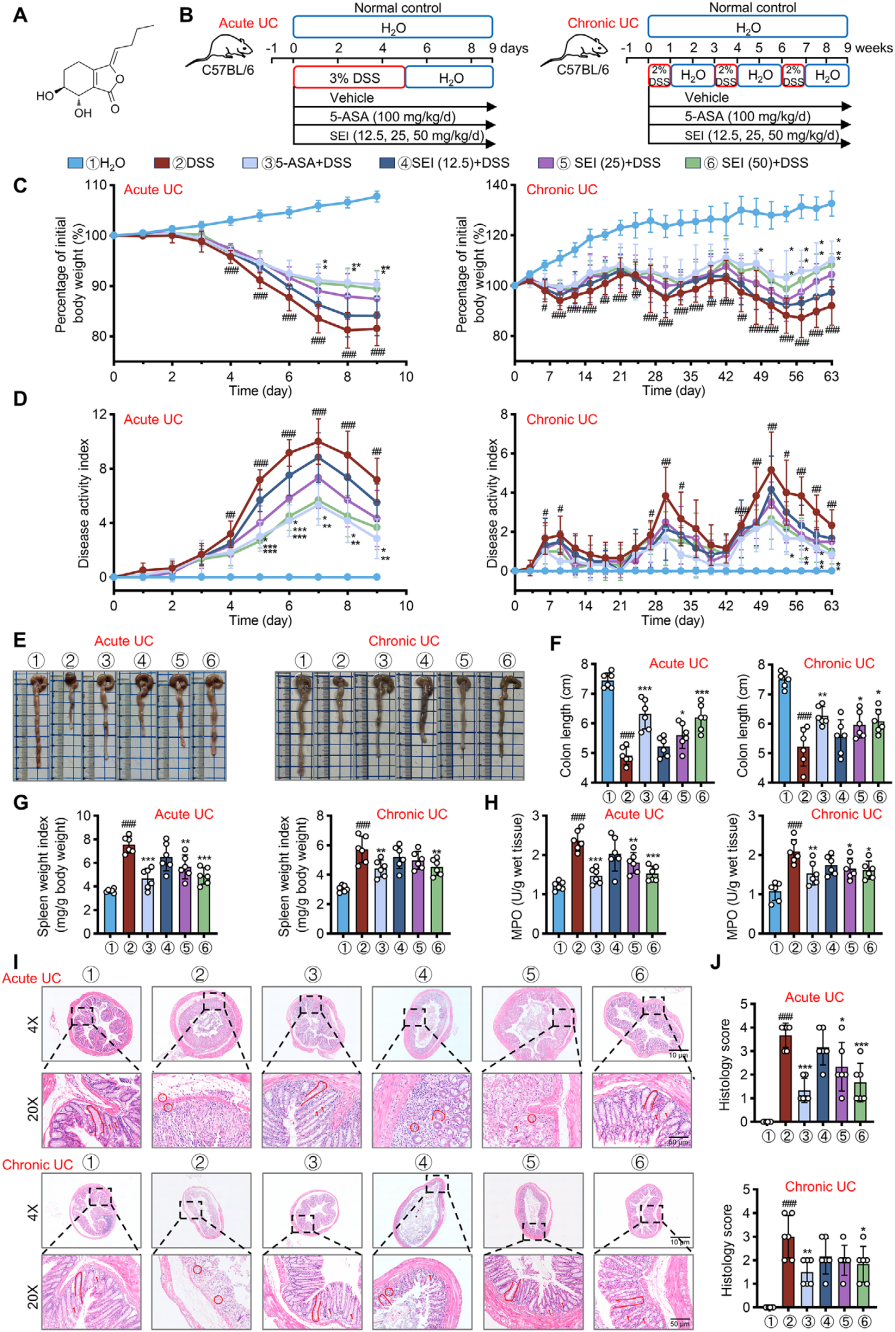
SEI alleviates disease symptoms in DSS‐induced colitis mice. (A) Chemical structure of SEI. (B) Schematic illustration of the animal experiment (*n* = 6 for each group). (C) Daily assessments of body weight change and (D) DAI were conducted. (E) Gross morphology images of the colon were captured on day 9 or day 63 after DSS treatment, and (F) colon length was measured. (G) The spleen index of mice. (H) Myeloperoxidase (MPO) levels in the colon were measured. (I) Colonic sections from mice were subjected to H&E staining (*U*‐shaped curve: *U*‐shaped crypt; arrow: goblet cell; circle: inflammatory cells), and (J) a semiquantitative histological score was assessed. Values were expressed as mean ± SD (*n* = 6). ^##^ *p* < 0.01, ^###^ *p* < 0.001 versus H_2_O group; *p < 0.05, **p < 0.01, ***p < 0.001 versus DSS group.

### 2.2 SEI Relieves Intestinal Barrier Damage in Mice With Colitis

Loss of intestinal barrier integrity represents one of the most prominent pathological features of UC [35]. Assessment of intestinal permeability using FITC‐Dextran revealed that SEI and 5‐ASA effectively reversed the DSS‐induced elevation in intestinal permeability (Figure 2A; Figure S1A). The intestinal mucus barrier, primarily composed of mucus secreted by goblet cells, serves as the host's primary defense against external pathogens and safeguards the intestinal epithelium [36]. Alcian Blue‐Periodic Acid‐Schiff (AB‐PAS) staining demonstrated that DSS‐induced colitis in mice decreased mucin secretion (stained blue) by goblet cells, resulting in a thinner mucus layer, whereas both SEI and 5‐ASA promoted mucin secretion, thereby restoring mucus layer thickness (Figure 2B). Western blot (WB) and immunofluorescence (IF) analyses further confirmed that SEI and 5‐ASA upregulated mucin 2 (MUC2) expression (Figure 2C,D; Figure S1B,C). Tight junction proteins, critical components of the intestinal epithelial barrier, are key indicators of intestinal epithelial integrity [37]. WB and IF analyses demonstrated that SEI and 5‐ASA reversed the DSS‐induced downregulation of tight junction proteins (ZO1, Occludin, and Claudin1), with the high‐dose SEI (50 mg/kg/day) exerting the most potent restorative effect (Figure 2C,D; Figure S1B,C). Trefoil factor 3 (TFF3), a pivotal regulator of intestinal mucosal barrier function, enhances mucus stability, facilitates epithelial repair, and modulates tight junction proteins [38]. SEI and 5‐ASA significantly upregulated TFF3 protein expression (Figure 2E). E‐cadherin is a key adhesion junction protein that interacts with β‐catenin to maintain intestinal barrier integrity and prevent pathogen penetration [39]. Ki67 is a proliferation marker that drives epithelial cell regeneration and repair following intestinal injury [40]. Additionally, SEI and 5‐ASA markedly elevated E‐cadherin and Ki67 protein levels (Figure 2F). Collectively, these findings demonstrate that SEI restores intestinal barrier function disrupted in DSS‐induced colitis.

**FIGURE 2:**
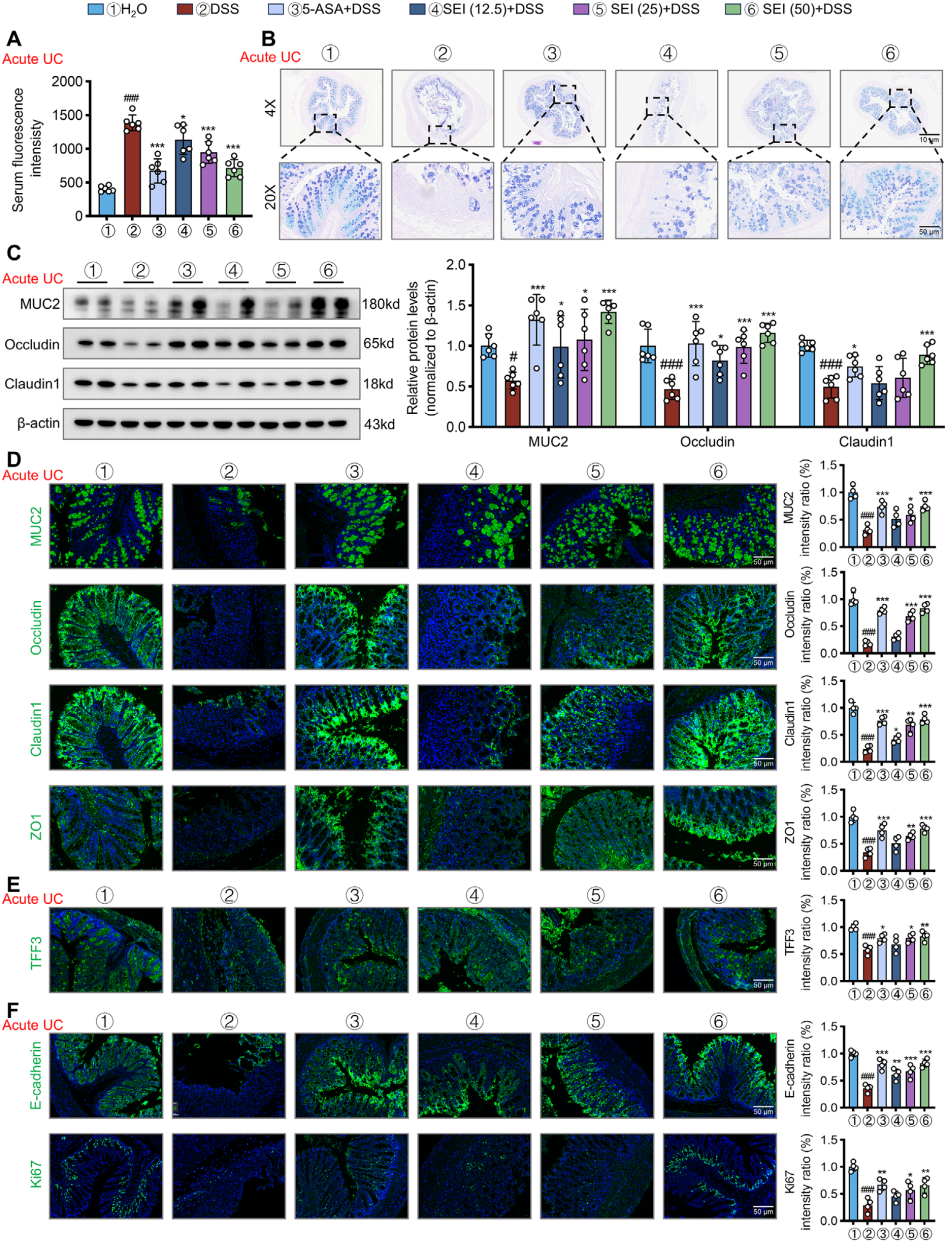
SEI relieves intestinal barrier damage in mice with colitis. (A) The serum fluorescence intensity was measured after administering FITC‐dextran (*n* = 6). (B) Representative AB‐PAS staining images. (C) The protein expression of MUC2, Occludin and Claudin1 in the colonic tissues was determined by WB (*n* = 6). (D) Representative fluorescent images of MUC2, Occludin, Claudin1 and ZO1 in the colonic tissues and their relative quantification (*n* = 4). (E) Representative fluorescent images of TFF3 in the colonic tissues and their relative quantification (*n* = 4). (F) IF staining for Ki67 and E‐cadherin in colon tissues and their relative quantification (*n* = 4). Scale bar = 50 µm. Values were expressed as mean ± SD. ^#^ *p* < 0.05, ^###^ *p* < 0.001 versus H_2_O group; *p < 0.05, **p < 0.01, ***p < 0.001 versus DSS group.

### 2.3 SEI Inhibits DSS‐Induced Colonic Inflammation and Suppresses M1 Polarization of Macrophages

The inflammatory cytokine storm, characterized by elevated levels of IL‐1β, IL‐6, TNF‐α, and IL‐23, represents a central component in the pathogenesis of UC [41]. To evaluate the effect of SEI on colonic inflammation, we measured these cytokines using quantitative real‐time PCR (qRT‐PCR) and ELISA. Both methods showed that SEI and 5‐ASA significantly reduced their expression in colon tissues (Figure 3A,B; Figure S2A,B). Upon inflammatory stimulation, macrophages undergo a continuum of functional reprogramming, wherein pro‐inflammatory (M1‐like) and anti‐inflammatory (M2‐like) phenotypes constitute two polar extremes that jointly govern inflammatory progression and tissue homeostatic balance [42]. Given that SEI can inhibit the M1 macrophage‐secreted cytokines (IL‐1β, IL‐6, TNF‐α, and IL‐23), we examined its effect on macrophage polarization. Immunoblot analysis showed that DSS treatment increased the expression of CD86 and iNOS (M1 markers). However, SEI and 5‐ASA significantly reduced the expression of CD86 and iNOS (Figure 3C), as well as the colocalization of CD86 and F4/80 (Figure 3D,E; Figure S2C,D), which indicates a decrease in M1 macrophage infiltration. Flow cytometry data further confirmed that the proportion of CD86^+^ M1‐type macrophages in the colon was higher in the DSS group than in the normal control group. However, SEI reversed these changes while increasing the proportion of CD206^+^ M2‐type macrophages (Figure 3F,G). These results suggest that SEI reduces macrophage infiltration and inhibits M1 macrophage polarization, thereby exerting anti‐inflammatory effects in UC mice.

**FIGURE 3:**
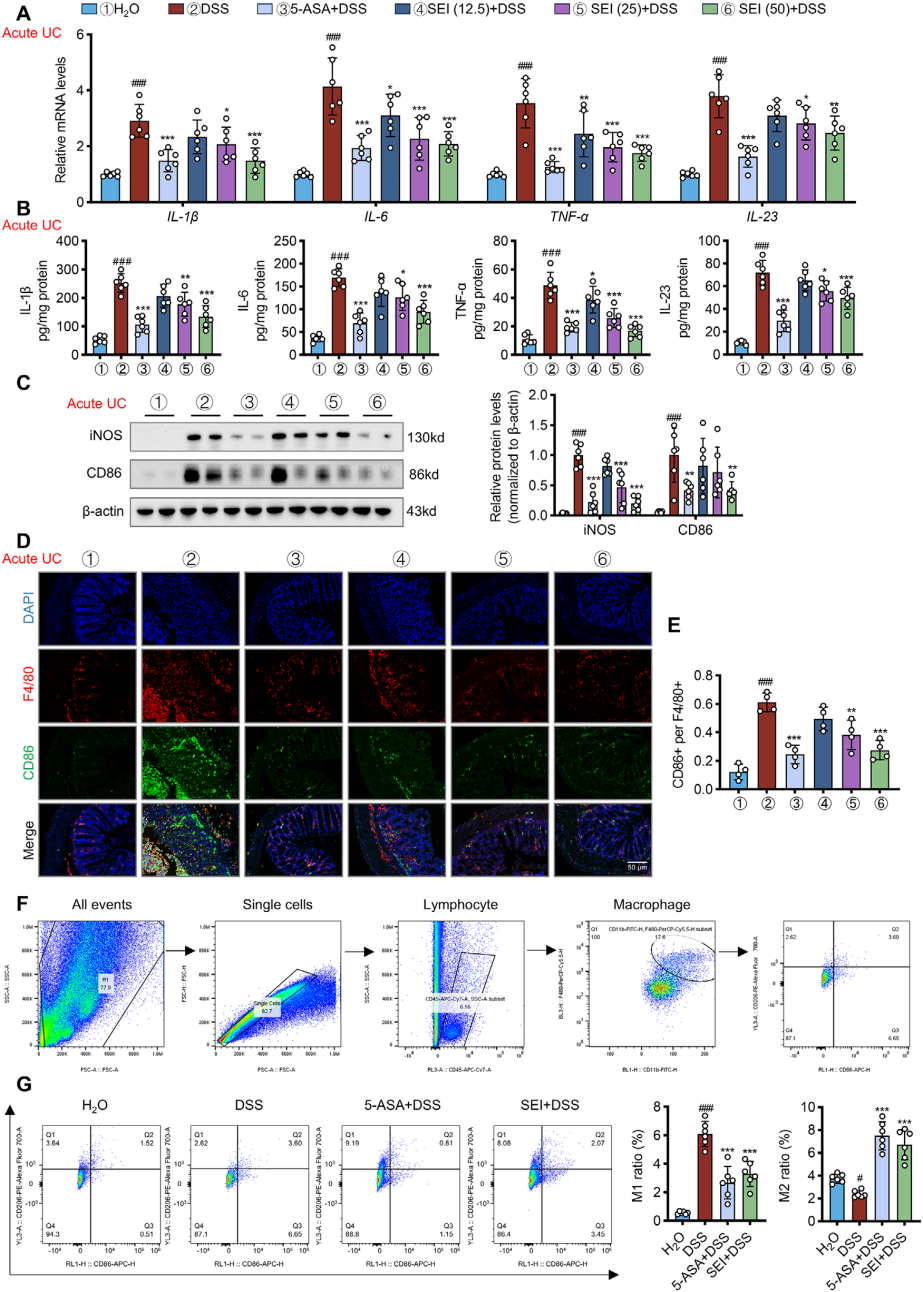
SEI inhibits DSS‐induced colonic inflammation and suppresses M1 polarization of macrophages. (A) qRT‐PCR analysis of inflammatory cytokines (*IL‐1β*, *IL‐6*, *TNF‐α* and *IL‐23*) (*n* = 6). (B) ELISA for inflammatory cytokine production in colonic tissue, including IL‐1β, IL‐6, TNF‐α and IL‐23 (*n* = 6). (C) The protein expression of iNOS and CD86 in colonic tissue was determined by WB (*n* = 6). (D) Representative immunofluorescence images of colonic tissue stained for F4/80 (red) and CD86 (green) with DAPI (blue) for nuclear counterstaining. Scale bar = 50 µm. (E) Relative fluorescence intensity of CD86^+^F4/80^+^ cells was quantified (*n* = 4). (F) Flow cytometry gating strategy. (G) Representative flow cytometry dot plot. SEI reduced the levels of CD86^+^ M1‐type macrophages in the colonic lamina propria and increased the levels of F4/80^+^CD206^+^ M2‐type macrophages. Bars are color‐coded to represent experimental groups: light blue = H_2_O‐treated normal control; red = DSS‐induced acute UC model; pale blue = 5‐ASA‐treated positive control (co‐administered with DSS); dark blue = low‐dose SEI (12.5 mg/kg) + DSS; purple = medium‐dose SEI (25 mg/kg) + DSS; green = high‐dose SEI (50 mg/kg) + DSS. Values were expressed as mean ± SD. ^#^ *p* < 0.05, ^###^ *p* < 0.001 versus H_2_O group; *p < 0.05, **p < 0.01, ***p < 0.001 versus DSS group.

To further investigate the in vitro effects of SEI, we first cultured iBMDM cells, a widely used immortalized mouse bone marrow‐derived macrophage cell line in immunology and cell biology research [43]. Subsequently, these cells were exposed to different concentrations of SEI, and the cytotoxicity of SEI was assessed using the CCK‐8 assay. SEI at concentrations equal to or lower than 100 µm for 24 h did not affect cell survival, indicating no significant toxicity to iBMDM cells (Figure S2E). Next, we cultured iBMDM cells and exposed them to TNF‐α plus IFN‐γ for 24 h to mimic an inflammatory cellular state and induce M1 polarization [44, 45], while simultaneously treating them with different doses of SEI. qRT‐PCR results showed that SEI dose‐dependently decreased the mRNA expression of pro‐inflammatory cytokines including IL‐1β, IL‐6, TNF‐α, and IL‐23, in response to TNF‐α plus IFN‐γ exposure (Figure S2F). Concurrently, SEI also dose‐dependently reduced the mRNA expression of genes associated with M1 macrophage polarization, including iNOS and CD86 (Figure S2G).

### 2.4 SEI Inhibits the Activation of mtDNA‐cGAS‐STING Inflammatory Signaling Pathway

To further investigate the underlying mechanism by which SEI inhibits UC, we performed a global proteomic analysis of mouse colon tissues (Figure 4A). Principal component analysis (PCA) and heatmap revealed significant differences between the UC (DSS + saline) and SEI (DSS + SEI) groups (Figure 4B,C). Compared with the UC group, 102 proteins were significantly decreased (fold change < 0.5 and *p*‐value < 0.05) and 34 proteins increased (fold change > 1.2 and *p*‐value < 0.05) in the SEI‐treated group. Then, Gene Ontology (GO) and Kyoto Encyclopedia of Genes and Genomes (KEGG) pathway analyses were applied to analyze the differentially expressed proteins and indicated that SEI treatment resulted in downregulated expression of proteins associated with biological processes including inflammatory response, ferroptosis, cytoplasmic DNA sensing pathways, and cellular responses to IFN‐I (Figure 4D). A heatmap showed that cGAS‐STING pathway‐associated proteins were significantly downregulated in SEI‐treated colitis mice compared with untreated colitis mice (Figure 4E). In addition, a deep learning model, the graph convolutional network‐based drug “on‐target” pathway prediction algorithm (GDOP), was used to predict the potential target signaling pathways of SEI. The top 10 “targeted” signaling pathways of SEI predicted by the GDOP model were shown in Figure 4F,G, and the full prediction results were detailed in Table S1. Notably, the fourth‐ranked pathway “Cytosolic sensors of pathogen‐associated DNA” and the ninth‐ranked pathway “STING‐mediated induction of host immune responses” suggested the regulation of SEI on the cGAS‐STING signaling pathway activated by cytoplasmic DNA.

**FIGURE 4:**
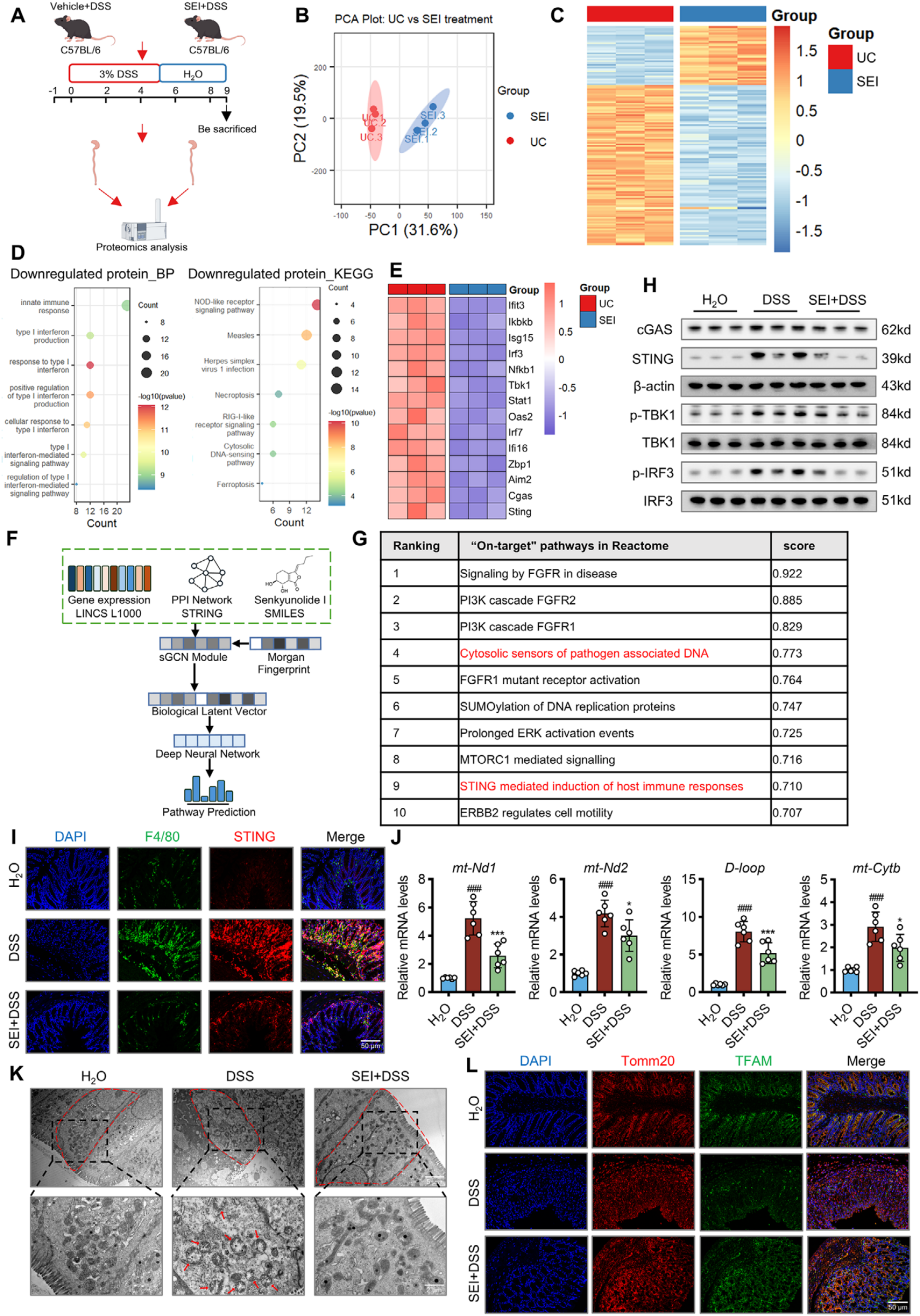
SEI inhibits the activation of the mtDNA‐cGAS‐STING inflammatory signaling pathway. (A) Proteomics analysis was performed on the colons of mice in the DSS group and the SEI (50 mg/kg/day) treatment group. For each group, two colons were pooled as one sample, and duplicate samples were tested (*n* = 3 biological replicates per group). (B) Two‐dimensional spatial distribution diagram of proteomics PCA of colon tissue from DSS group mice and SEI treatment group mice (*n* = 3). (C) Proteomic heatmaps of colonic tissues from DSS and SEI‐treated mice (*n* = 3). (D) GO analysis and KEGG analysis showing that SEI treatment altered the expression of proteins involved in several biologic processes, including the cGAS‐STING pathway. (E) Heatmap showing that cGAS‐STING pathway‐associated proteins were significantly downregulated in SEI‐treated colitis mice compared with untreated colitis mice. (F) The gene expression signature induced by SEI and the protein–protein interaction (PPI) network were integrated via a spectral‐based Graph Convolutional Network (sGCN) module to generate a biological latent vector. Concurrently, the SMILES notation of SEI was encoded into a Morgan fingerprint. These two representations were concatenated and processed by a deep dense network to predict the likelihood of SEI targeting specific biological pathways. (G) The top 10 predicted “on‐target” pathways for SEI. (H) WB analysis of the protein levels of cGAS, STING, TBK1, p‐TBK1, IRF3, and p‐IRF3 in colonic tissue (*n* = 6). (I) IF staining for F4/80 (green) and STING (red) in colon tissue. (J) The mRNA expression levels of *mt‐Nd1*, *mt‐Nd2*, *D‐loop*, and *mt‐Cytb* were measured by qRT‐PCR (*n* = 6). (K) The ultrastructure of mouse colon tissue was examined using TEM, revealing damaged mitochondria (red arrows) in intestinal epithelial cells (red circles). Scale bars = 5 µm. Original magnification ×2000. (L) IF staining for Tomm20 (red) and TFAM (green) in colonic tissue (*n* = 4). Scale bar = 50 µm. Values were expressed as mean ± SD. ^###^ *p* < 0.001 versus H_2_O group; *p < 0.05, **p < 0.01, ***p < 0.001 versus DSS group.

Furthermore, WB and IF also confirmed the inhibitory effect of SEI on the cGAS‐STING‐signaling cascade (Figure 4H; Figure S3A,B). Subsequent qRT‐PCR verified the downregulatory effects of SEI on key downstream targets in the cGAS‐STING pathway, such as IFNB, CCL5, CXCL10, IFIT1, and ISG15 (Figure S3C). Double IF results further showed that STING was predominantly colocalized with the macrophage marker F4/80, and SEI treatment reduced STING expression in macrophages (Figure 4I; Figure S3D).

Cytoplasmic ssDNA or dsDNA can activate STING and initiate downstream signaling [2]. Compared with the H_2_O group, the expression of mtDNA‐related genes was significantly upregulated in colonic tissue from the DSS group, while SEI treatment significantly reversed this change (Figure 4J). Mitochondrial damage, which stems from the opening of the mitochondrial permeability transition pore (mPTP) and the disruption of cristae, is one of the primary sources of mtDNA release [2]. Transmission electron microscopy (TEM) revealed that mitochondria in the UC mice appeared enlarged and rounded with reduced matrix density, shortened or disrupted cristae, and vacuolar degeneration, while SEI treatment improved these changes (Figure 4K). The mitochondrial transcription factor TFAM stabilizes mtDNA by binding to it and promotes autophagic clearance of cytoplasmic mtDNA [46]. IF results demonstrated that SEI reversed DSS‐induced TFAM downregulation (Figure 4L; Figure S3E). Collectively, these findings indicate that SEI inhibits cGAS‐STING pathway activation by preserving mitochondrial integrity and minimizing dsDNA leakage.

To investigate the effect of SEI on the activation of the cGAS‐STING pathway, we selected macrophages for further analysis, as this pathway is mainly activated in macrophages during inflammatory conditions like DSS‐induced colitis [47]. The results showed that SEI concentration‐dependently inhibited TNF‐α plus IFN‐γ‐induced phosphorylation of STING, TBK1, and IRF3 in iBMDMs (Figure S4A,B). A similar phenomenon was also observed in THP‐1‐differentiated macrophages (Figure S4C,D). Consistent with in vivo results, SEI apparently suppressed the expression of IRF3‐responsive genes (IFNB, CCL5, CXCL10, ISG15 and IFIT1) in iBMDM cells and THP‐1 cells (Figure S4E–N). We also examined cytosolic mtDNA through qRT‐PCR analysis by adopting primers specific to mtDNA sequences. TNF‐α combined with IFN‐γ stimulates mtDNA release, whereas SEI can reduce such release (Figure S4O).

To investigate whether this regulatory relationship is widespread among macrophages, we isolated macrophages derived from colonic tissue and peritoneal macrophages from mice and cultured them in primary cultures. The results showed that SEI was equally capable of inhibiting the phosphorylation of STING, TBK1, and IRF3 in both colonic tissue‐derived macrophages and peritoneal macrophages stimulated by TNF‐α and IFN‐γ (Figure S4P–S), suggesting that SEI suppresses the cGAS‐STING pathway across multiple macrophage populations, rather than targeting only a single subset.

To further confirm that the anti‐colitic effect of SEI is mediated through the STING signaling pathway in vivo, we treated DSS‐induced colitis mice with the specific STING inhibitor H‐151 alone or in combination with SEI. As shown in Figure S5, both SEI and H‐151 monotherapy significantly ameliorated DSS‐induced colitis, as evidenced by attenuated body weight loss, reduced DAI scores, restored colon length, and decreased inflammatory cell infiltration (Figure S5A–G). Notably, the combination of SEI and H‐151 did not confer additional protection compared with either agent alone. These results support the conclusion that the therapeutic effect of SEI is dependent on suppressing STING signaling.

Collectively, these results demonstrate that SEI inhibits the cGAS‐STING pathway in macrophages by preserving mitochondrial integrity and reducing mtDNA release, and that this effect is essential for its anti‐colitic activity.

### 2.5 Activity‐Based Protein Profiling (ABPP) Proteomic Analysis Confirms VDAC1 as Target Protein of SEI

Given the significant therapeutic efficacy of SEI in UC, we employed ABPP to identify its molecular targets and mechanisms [48]. SEI‐P probes containing clickable alkyne tags were synthesized (Figure 5A). Click chemistry enabled the conjugation of Cy3 or biotin to SEI‐P, labeling SEI‐bound protein targets. Target proteins were subsequently identified via mass spectrometry or fluorescence imaging. SEI‐P retained the anti‐inflammatory activity of SEI, validating its utility as a surrogate for target identification and imaging (Figure 5B–D). Incubation of iBMDM cells with SEI‐P revealed uniform subcellular distribution of the probe, enabling comprehensive screening of intracellular targets for SEI (Figure 5E). We incubated TNF‐α plus IFN‐γ‐activated iBMDM cells with SEI‐P, and the proteins subsequently labeled by SEI‐P were click‐conjugated to the fluorescent dye Cy3‐azide (Figure 5F). For competitive ABPP, pre‐activated iBMDM cells were pretreated with or without SEI before 1 h labeling with 50 µm SEI‐P. Labeled proteins were conjugated to fluorescent dyes, separated by SDS‐PAGE, and visualized. SEI‐P broadly labeled cellular proteins, but pre‐incubation with 25, 50, or 100 µm SEI competitively abolished labeling (Figure 5G), confirming probe specificity. Biotinylated targets were enriched via streptavidin agarose and trypsin‐digested. Peptides were analyzed by quantitative mass spectrometry (Figure 5H). Among 975 identified proteins, 15 candidates met selection criteria (*p* < 0.05 and the log_2_ (fold change [FC]) of the competitive group (100 µm SEI + 50 µm SEI‐P) compared to the probe group SEI‐P (50 µm) was <−1; Figure 5I). Notably, VDAC1 exhibited a higher probe/competitor ratio with significantly enriched labeling. SDS‐PAGE showed a prominent ∼35 kDa band (consistent with VDAC1) in SEI‐P lanes that diminished in SEI‐competition lanes (Figure 5G). These results collectively identify VDAC1 as a direct target of SEI.

**FIGURE 5:**
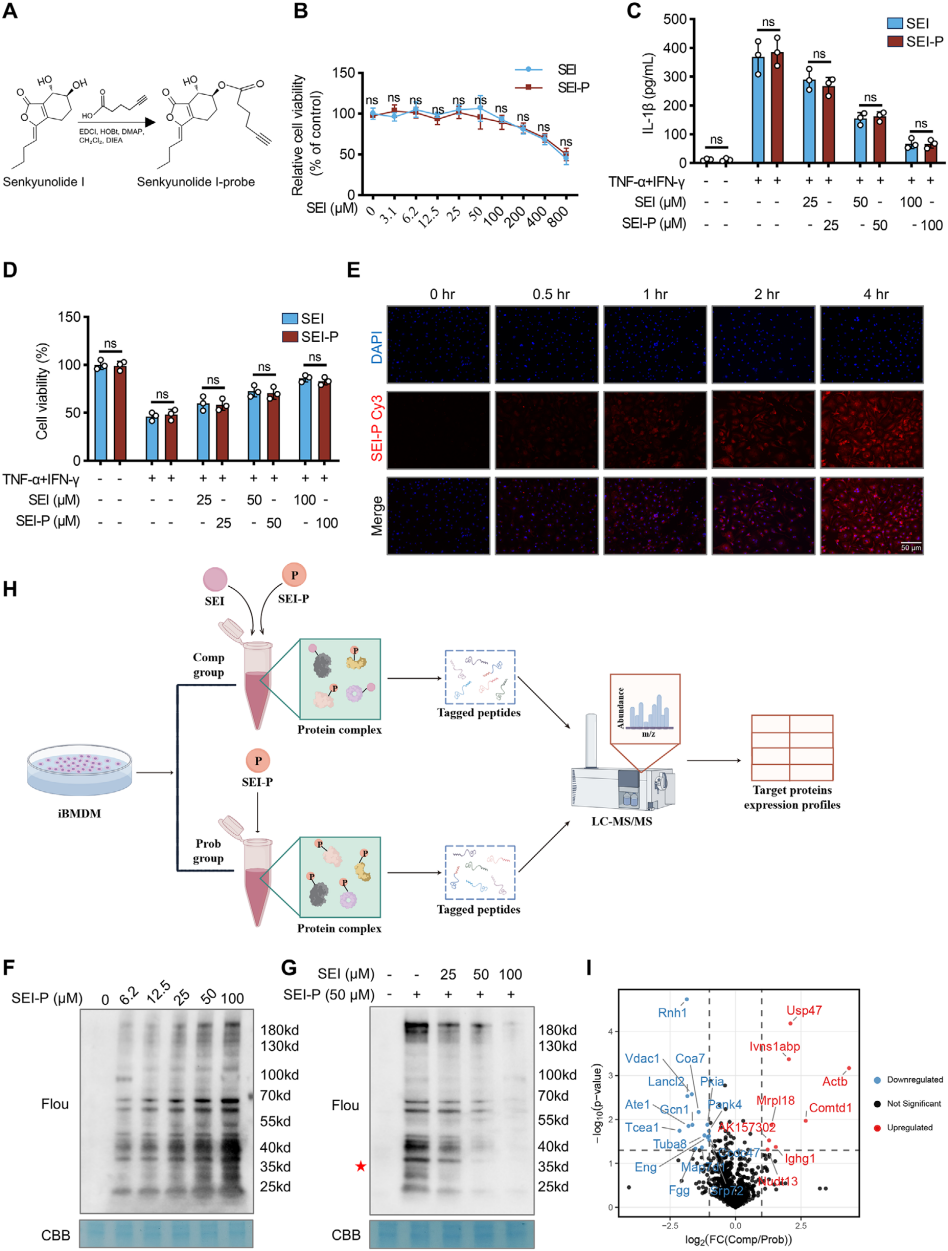
ABPP proteomic analysis confirms VDAC1 as target protein of SEI. (A) Chemical structures of SEI and SEI probe (SEI‐P). (B) Cell viability of iBMDM cells treated with SEI or SEI‐P. (C) Release of inflammatory cytokine IL‐1β and (D) cell viability in TNF‐α plus IFN‐γ‐induced iBMDM cells. (E) Cellular imaging of SEI‐P with different exposure times in iBMDM cells. (F) Dose‐dependent labeling of proteins by SEI‐P in iBMDM cells. (G) Competition between SEI and SEI‐P for protein binding in situ (red star: 35 kD). (H) Chemical proteomics analysis workflow for identifying potential targets of SEI, created using Figdraw. (I) Volcano plot of proteins identified in the ABPP method. The graph displayed the log_2_ FC of the competition group (100 µm SEI + 50 µm SEI‐P) versus SEI‐P (50 µm) (*x*‐axis) against the −log_10_(*p‐*value) (*y*‐axis). Among these, points with *p* < 0.05 and log_2_ *FC* < −1 (blue) were selected as target protein candidates. Values were expressed as mean ± SD (*n* = 3).

### 2.6 SEI Directly Interacts With VDAC1 to Inhibit its Oligomerization

Subsequently, molecular docking studies were conducted to investigate the interaction between SEI and VDAC1. AutoDock Vina docking simulations indicated that SEI exhibits moderate to strong binding affinity with VDAC1 (the lowest binding energy of −6.8 kcal/mol), forming four hydrogen bonds with Lys12, Tyr173, Gln179, and Leu180 (Figure 6A). Then, cellular thermal shift assay (CETSA) and drug affinity responsive target stability (DARTS) were used to further confirm this direct interaction (Figure 6B–D). Micro‐scale thermophoresis (MST) analysis unequivocally demonstrated a direct interaction between SEI and VDAC1 with strong binding affinity (*K_d_* = 28 µm) (Figure 6E).

**FIGURE 6:**
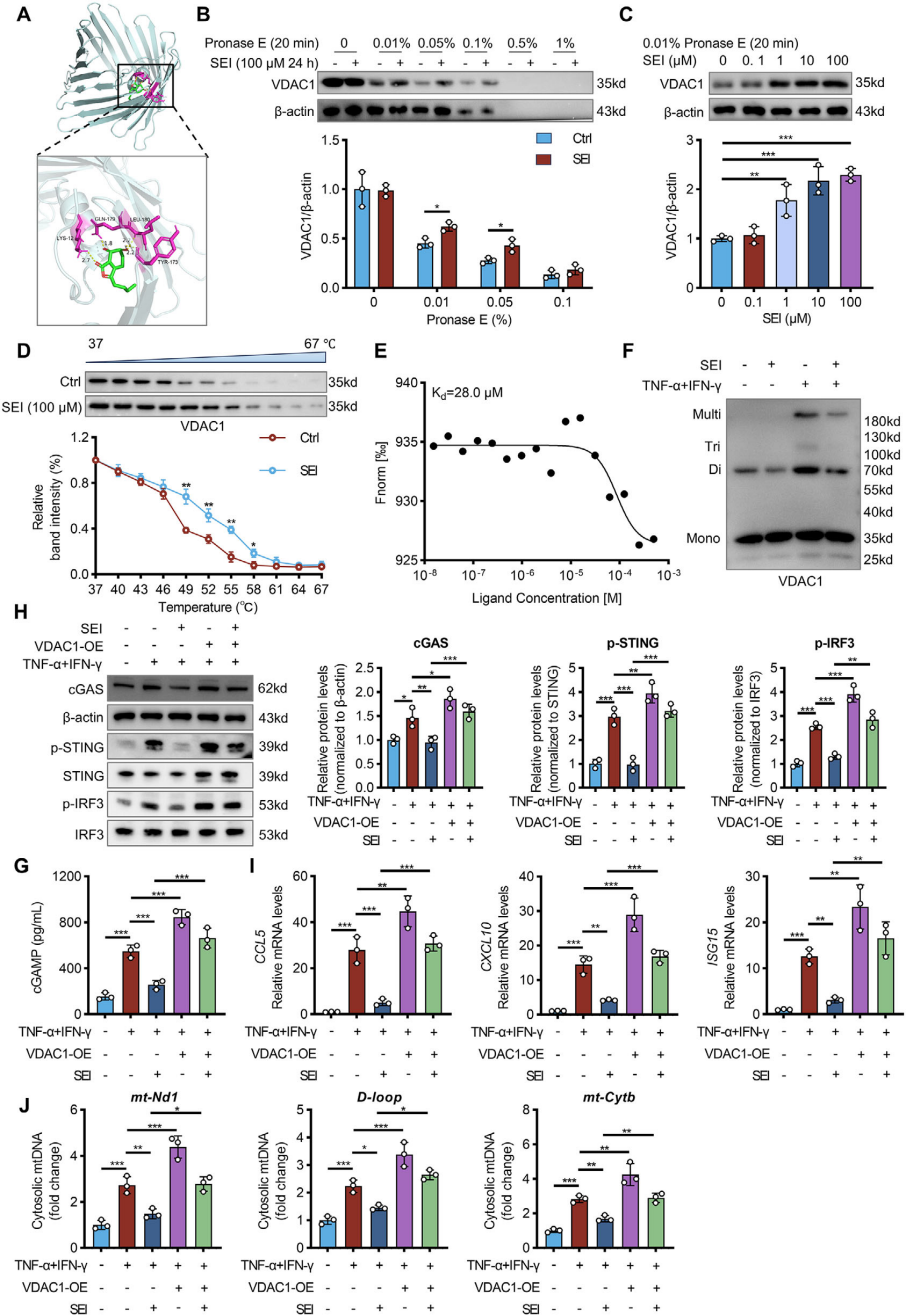
SEI directly interacts with VDAC1 to inhibit its oligomerization. (A) The interaction between SEI and VDAC1 was detected using molecular docking. (B) Lysates from iBMDM cells were incubated with or without SEI (100 µm) for 24 h. Different concentrations of pronase E were added for 20 min, and VDAC1 content was analyzed using WB analysis. (C) Lysates from iBMDM cells were incubated with SEI at the indicated concentrations for 24 h, with a final concentration of 0.01% pronase E added for 20 min. The level of VDAC1 was assessed through WB analysis. (D) iBMDM cells were incubated with SEI (100 µm) for 24 h. These samples were then analyzed using CETSA. Values were expressed as mean ± SD (*n* = 3). *p < 0.05, **p < 0.01 versus SEI group. (E) MST demonstrating a direct interaction between SEI and EGFP‐tagged VDAC1 in lysates from EGFP‐VDAC1 expressing HEK293T cells. (F) Immunoblotting analysis of VDAC1 cross‐linking in iBMDM cells, untreated or stimulated with TNF‐α plus IFN‐γ, with or without addition of SEI (100 µm). (G) The VDAC1‐OE plasmid was transfected into iBMDM cells, and cGAMP production was subsequently measured by ELISA. (H) iBMDM cells were transfected with VDAC1‐OE, and then the phosphorylation levels of STING and IRF3 were measured by WB. (I) The mRNA expression levels of *CCL5*, *CXCL10*, and *ISG15* in iBMDM cells transfected with VDAC1‐OE were measured by qRT‐PCR. (J) qRT‐PCR analysis of cytoplasmic mtDNA (*mt‐Nd1*, *D‐loop* and *mt‐Cytb*). Values were expressed as mean ± SD (*n* = 3). *p < 0.05, **p < 0.01, ***p < 0.001.

To investigate how SEI exerts its regulatory role on VDAC1 activity, we measured the oligomerization of VDAC1 using the VDAC1 cross‐linking assay [49]. Our results demonstrated that TNF‐α plus IFN‐γ increased VDAC1 oligomerization, while SEI administration significantly suppressed this oligomerization (Figure 6F). To further identify whether SEI‐mediated inhibition of the cGAS‐STING pathway is dependent on VDAC1, we examined the effect of VDAC1 overexpression on cGAMP production, cGAS expression levels, and STING and IRF3 phosphorylation levels in the presence of SEI. Transfection of the VDAC1 plasmid significantly increased the protein level of VDAC1 and impaired the inhibitory effect of SEI on cGAMP production, STING and IRF3 phosphorylation, and cGAS expression levels (Figure 6G,H; Figure S6A). Concurrently, overexpression of VDAC1 also suppressed the role of SEI in key downstream target genes of the cGAS‐STING pathway (Figure 6I). We then investigated the effects of VDAC1 overexpression on mtDNA levels in iBMDMs in the presence of SEI. Following transfection with the VDAC1 plasmid, the inhibitory effect of SEI on the release of mtDNA into the cytoplasm was also attenuated (Figure 6J). The above results demonstrate that SEI directly interacts with VDAC1 to inhibit its oligomerization, thereby exerting an inhibitory effect on the cGAS‐STING pathway.

### 2.7 SEI Binds Directly to VDAC1 at Residue K12 and Disrupts its Oligomerization

To elucidate the binding mode between SEI and VDAC1, molecular dynamics (MD) simulations were employed. The stability of the SEI‐VDAC1 complex was evaluated over a 100 ns simulation trajectory. Both root‐mean‐square deviation (RMSD) and root‐mean‐square fluctuation (RMSF) analyses demonstrated that the complex remained stable throughout the simulation. Notably, the key interacting residues exhibited minimal fluctuations, all below 1 Å (Figure 7A–C).

**FIGURE 7:**
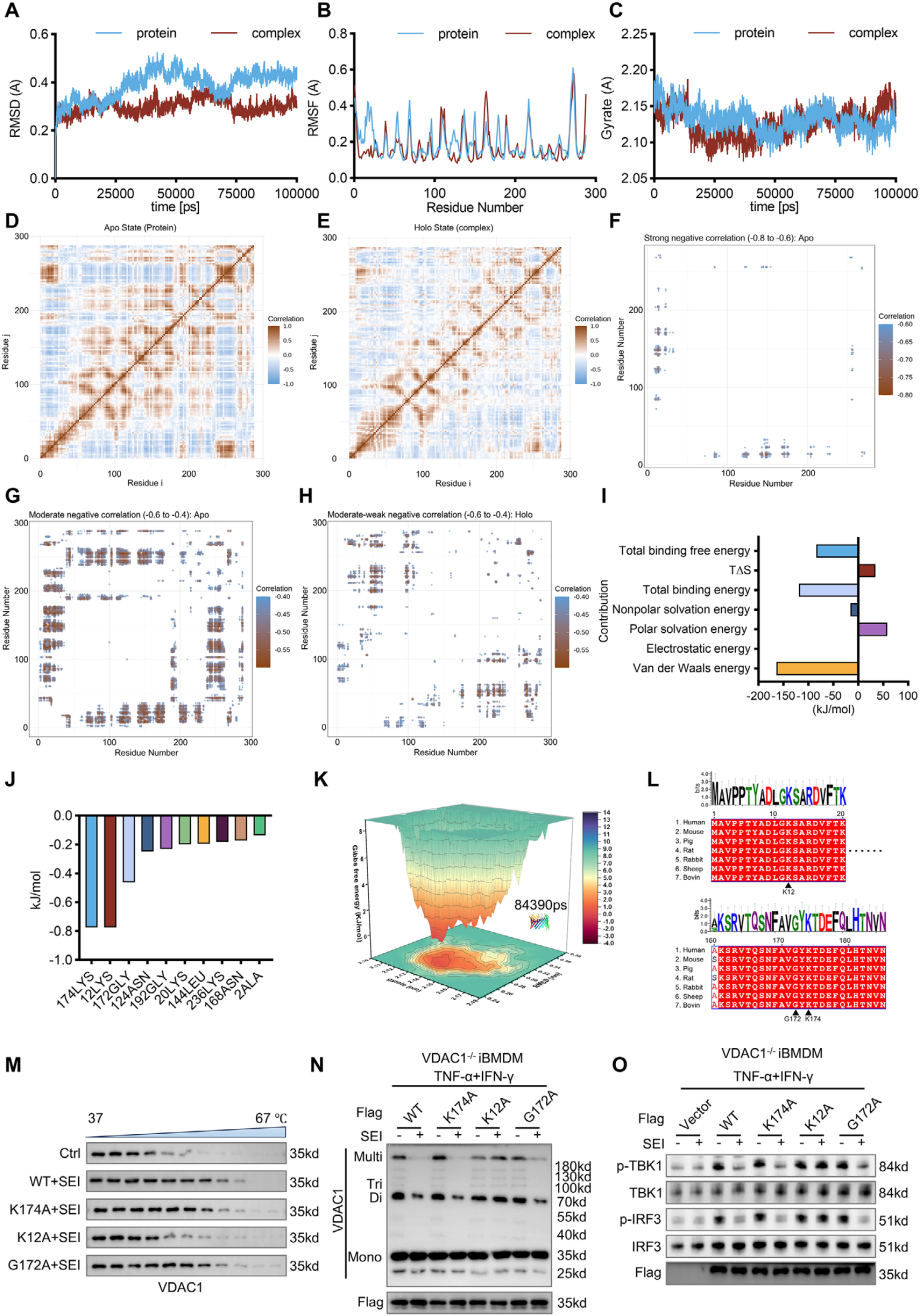
SEI directly binds to K12 on VDAC1. (A) The RMSD of the VDAC1 backbone was simulated for a range of 100 ns. (B) The RMSF values of all amino acid residues were simulated. (C) Radius of gyration of the apo (blue) and protein when bound to the ligand (red) for the 100 ns simulation. (D) DCCM analysis matrix of VDAC1 protein; the region in red indicates residue pairs in horizontal and vertical coordinates have positive correlation in movement patterns, while the region in blue indicates negative correlation. (E) DCCM analysis matrix of SEI‐VDAC1 complex. (F–H) Movement correlation of each residue with a negative correlation coefficient ranged from −0.6 to −0.8 of VDAC1 protein (F), ranged from −0.4 to −0.6 of VDAC1 protein (G), and ranged from −0.4 to −0.6 of SEI‐VDAC1 complex (H). (I) The total binding free energy was calculated, and a series of contribution components were analyzed. Data are presented as energy changes in units of kJ/mol. (J) Ten residues of the VDAC1‐SEI complex were selected and analyzed. Data were presented as energy changes in a unit of kJ/mol with different contributors indicated by colors. (K) Free energy landscape. (L) Sequence conservation analysis of VDAC1 protein using ESPript 3.0. (M) The iBMDM cells were transfected with K174, K12, and G172 mutation plasmids and then treated with DMSO or SEI (100 µm) for 1 h. The interaction between SEI and VDAC1 was detected using the CETSA assay. Values were expressed as mean ± SD (*n* = 3). *p < 0.05, **p < 0.01, ***p < 0.001 versus Ctrl group. (N) VDAC1^−/−^ iBMDM cells were transfected with Flag‐VDAC1(WT), Flag‐VDAC1(K174A), Flag‐VDAC1(K12A), or Flag‐VDAC1(G172A). Immunoblotting analysis of VDAC1 cross‐linking in iBMDM cells stimulated with TNF‐α plus IFN‐γ, with or without addition of SEI (100 µm). (O) VDAC1^−/−^ iBMDM cells were transfected with an empty vector, Flag‐VDAC1‐WT, Flag‐VDAC1(K174A), Flag‐VDAC1(K12A), or Flag‐VDAC1(G172A). WB analyses of the quantity of p‐TBK1 and p‐IRF3 after stimulation with TNF‐α plus IFN‐γ and treatment with SEI (100 µm) or left untreated (control) for 24 h. Values were expressed as mean ± SD (*n* = 3). *p < 0.05, **p < 0.01, ***p < 0.001.

To gain deeper insight into how SEI binding influences the intrinsic motions of VDAC1, we performed dynamic cross‐correlation matrix (DCCM) analysis. This analysis maps coordinated motions between residue pairs. In the apo‐VDAC1 simulation, we observed widespread negative correlations, indicative of antagonistic, twisting motions between different structural domains (Figure 7D). This highlights dynamic interactions within the protein core that may contribute to instability. Strikingly, SEI binding markedly diminished these negative correlations across the VDAC1 structure (Figure 7E). The pronounced reduction in medium‐strength negative correlations (in the −0.4–−0.6 range) signifies that SEI restricts the inherent flexible dynamics of VDAC1, effectively “locking” it into a more rigid conformation (Figure 7F–H). This SEI‐induced rigidification is consistent with a stabilization of a specific VDAC1 state unfavorable for large‐scale conformational rearrangements.

The binding free energy for the SEI‐VDAC1 complex was calculated as −118.59 kJ/mol using the MM/PBSA method, with van der Waals forces being the major contributor (−163.57 kJ/mol) (Figure 7I). Notably, ten specific residues were identified as crucial contributors to the total binding energy (−118.59 kJ/mol) (Figure 7J). Analysis of the free energy landscape pinpointed the most favorable binding conformation at 84 390 ps (Figure 7K). Conservation analysis of the K12, G172, and K174 residues in VDAC1 indicated that these residues exhibited high conservation across multiple species (Figure 7L). Guided by these in silico predictions, site‐directed mutants (K12A, G172A, and K174A) were constructed for experimental validation. CETSA confirmed that these alanine substitutions altered the thermal stability of the VDAC1‐SEI complex (Figure 7M; Figure S7A). The stability curve for K12A shifted more toward the blank group than the curves of G172A and K174A, indicating that mutation of K12 abolished the binding of SEI to VDAC1. We further investigated the effects of these mutations on VDAC1 oligomerization. The experimental results showed that the G172A and K174A mutations partially impaired the inhibitory effect of SEI on VDAC1 oligomerization, while the K12A mutation markedly impaired this effect (Figure 7N; Figure S7B). This suggested that SEI primarily suppressed VDAC1 oligomerization by targeting and binding to K12. We then examined the roles of these mutations in SEI‐mediated inhibition of cGAS‐STING signaling. The K12A mutant significantly impaired the inhibitory effect of SEI on TBK1 and IRF3 phosphorylation and downstream pathway activation (Figure 7O; Figure S7C,D). In contrast, G172A and K174A mutations exhibited only minor effects (Figure 7O; Figure S7C,D). These findings show that SEI suppresses the STING‐TBK1‐IRF3 signaling cascade by targeting the K12 residue on VDAC1, consistent with a prior study indicating that positively charged residues in the N‐terminal domain of VDAC1, especially K12, are critical for oligomerization [14].

### 2.8 SEI Mitigates Mitochondrial Damage‐Induced Oxidative Stress and Attenuates Both Pyroptosis and Ferroptosis in UC Mice

VDAC1 oligomerization forms pores in the mitochondrial outer membrane, leading to mitochondrial damage and excessive ROS generation [50]. Therefore, we next investigated whether SEI reduced ROS production. As shown in Figure 8A–F, SEI treatment significantly reduced ROS production and attenuated the DSS‐induced elevation in MDA levels as well as the reduction in antioxidant enzyme levels, including SOD, CAT, GPX, and GSH, in UC mice. We next investigated the protective effect of SEI on mitochondrial function in vitro. Exposure to TNF‐α plus IFN‐γ caused mitochondria to become spherical and shortened, but this was reversed by SEI treatment (Figure S8A). Additionally, TNF‐α plus IFN‐γ exposure significantly disrupted mitochondrial membrane potential, as shown by increased green fluorescence and reduced red fluorescence, while SEI treatment decreased JC‐1 monomer formation, indicating improved mitochondrial function (Figure S8B).

**FIGURE 8:**
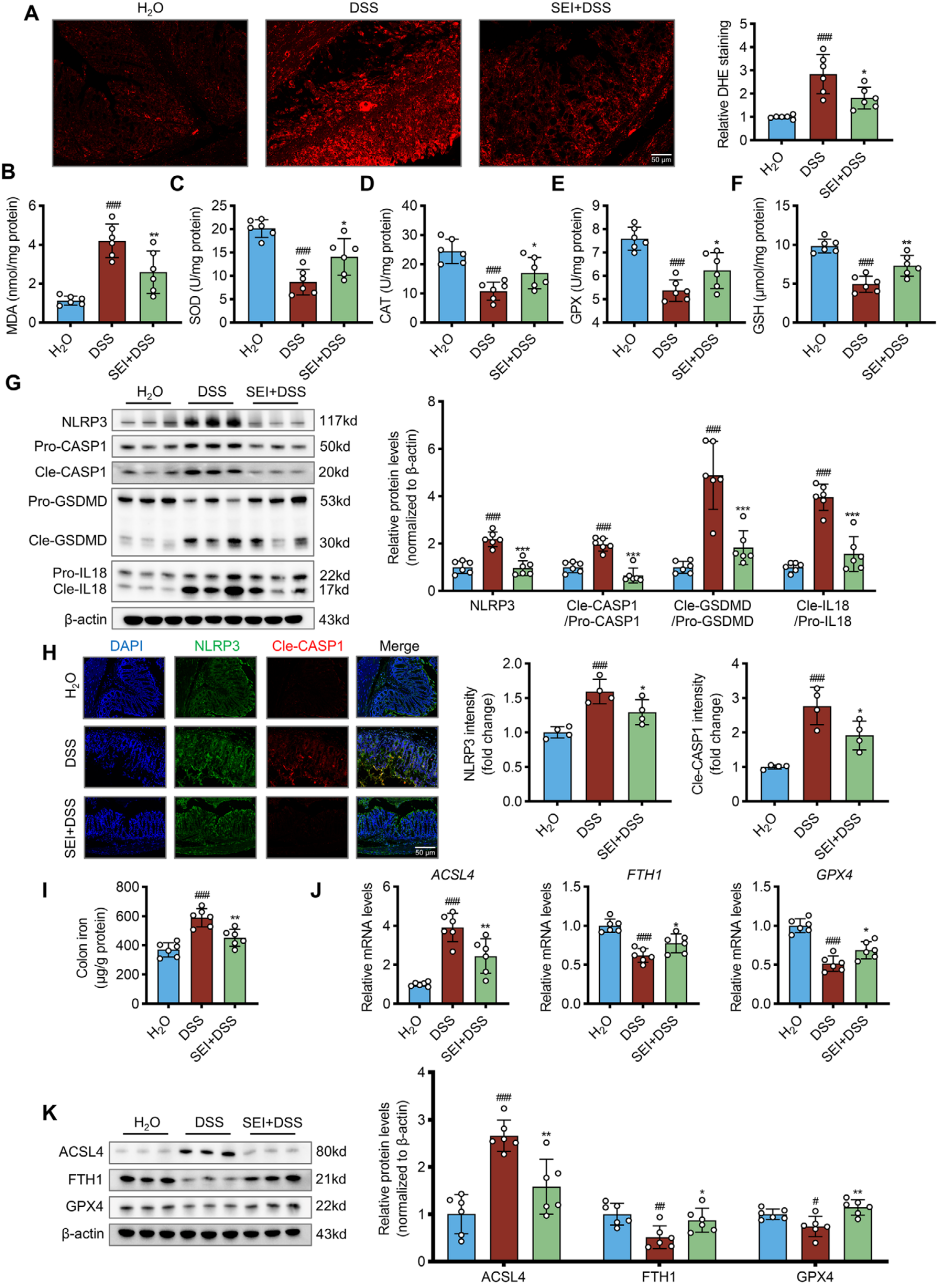
SEI alleviates mitochondrial damage‐induced oxidative stress and reduces intestinal cell pyroptosis and ferroptosis in colonic tissues of UC mice. (A) Representative images of DHE staining showing increased ROS production in colonic tissue and their relative quantification (*n* = 6). (B) MDA content in colonic tissue. Antioxidant enzyme (C) SOD, (D) CAT, (E) GPX and (F) GSH content in colonic tissue (*n* = 6). (G) WB analysis of the protein levels of NLRP3, CASP1, GSDMD and IL18 in colonic tissue (*n* = 6). (H) IF staining for NLRP3 (green) and Cle‐CASP1 (red) in colonic tissue (*n* = 4). (I) Iron levels of colonic biopsy tissue were determined by the Iron Assay Kit (*n *= 6). (J) The mRNA expression levels of *ACSL4*, *FTH1*, and *GPX4* were measured by qRT‐PCR (*n* = 6). (K) WB analysis of the protein levels of ACSL4, FTH1, and GPX4 in colonic tissue (*n* = 6). Scale bar = 50 µm. Values were expressed as mean ± SD. ^#^ *p* < 0.05, ^##^ *p* < 0.01, ^###^ *p* < 0.001 versus H_2_O group; *p < 0.05, **p < 0.01, ***p < 0.001 versus DSS group.

Accumulating evidence indicates that mitochondrial damage triggers NLRP3 inflammasome assembly, leading to CASP1 activation and intestinal pyroptosis [51, 52]. Therefore, we assessed the role of SEI on NLRP3 inflammasome and pyroptosis and found that SEI effectively suppressed NLRP3 inflammasome activation and associated pyroptosis in colon tissues of UC mice (Figure 8G,H) and iBMDM cells (Figure S8C–E).

In addition to inducing pyroptosis, ROS can also oxidize membrane polyunsaturated fatty acids through lipoxygenases or Fenton reactions, leading to the formation of lipid peroxides and subsequently triggering ferroptosis [50]. SEI significantly reduced colonic iron levels and modulated the expression of key ferroptosis‐related proteins, including ACSL4, FTH1, and GPX4, in DSS‐induced colitis mice (Figure 8I–K). Consistently, in vitro experiments confirmed that SEI treatment reduced ferroptosis and restored antioxidant system balance (Figure S8F–J). These findings collectively indicate that SEI mitigates oxidative stress‐induced pyroptosis and ferroptosis in colon tissue.

To determine whether the anti‐ferroptotic effect of SEI contributes to its therapeutic action in vivo, we treated mice with DSS‐induced colitis using a combination of the specific ferroptosis agonist erastin and SEI. As shown in Figure S9A–G, the coadministration of erastin markedly reversed the protective effects of SEI, as evidenced by aggravated body weight loss, elevated DAI scores, shortened colon length, and more severe colonic histopathological damage in the combination group compared to the SEI + DSS group. These results indicate that the anti‐colitic effect of SEI is, at least in part, mediated through the inhibition of ferroptosis. Together with the observed reduction in ferroptosis markers (Figure 8I–K), these data further confirm that suppression of ferroptosis is a critical downstream event contributing to the anti‐inflammatory effect of SEI.

### 2.9 The VDAC1 K12 Site is Required for the Protective Roles of SEI in UC Mice

To further validate the mechanism by which SEI alleviates DSS‐induced UC by targeting the K12 site of VDAC1, we established mouse models overexpressing VDAC1‐WT, VDAC1‐K12A, and a negative control (EGFP) in the colon by intraperitoneal injection of adeno‐associated virus (AAV) 9 carrying the VDAC1^WT^ or VDAC1^K12A^ gene. Four weeks after the injection of AAV9, fluorescence imaging of the tissues showed successful infection of the colon by AAV9 and increased expression of EGFP protein in the colonic tissues (Figure S10A). Moreover, at the end of the experiment, we also detected VDAC1 overexpression in each group by WB (Figure S10B). In the UC model, overexpression of VDAC1^K12A^ significantly abrogated SEI‐mediated colonic protection. This was manifested by increased weight loss, elevated DAI scores, shortened colon length, increased splenic index, neutrophilic infiltration, and extensive inflammatory cell infiltration in the colonic mucosa (Figure 9A–G). In addition, overexpression of VDAC1^K12A^ impaired the protective effect of SEI on intestinal barrier function (Figure 9H–J; Figure S10C,D). Notably, overexpression of VDAC1^K12A^ diminished the effect of SEI on reducing intestinal inflammatory responses and M1 polarization of macrophages (Figure 9K–L; Figure S10E). Finally, we evaluated the safety of SEI in vivo, and the results showed no noticeable toxic effects or histological changes in major organs (heart, liver, spleen, kidneys, and colon) at the tested doses (Figure S11A–C). Overall, these findings highlight a key mechanism by which SEI exerts a protective effect against UC development in vivo by targeting the VDAC1 K12 site and thus inhibiting the cGAS‐STING pathway.

**FIGURE 9:**
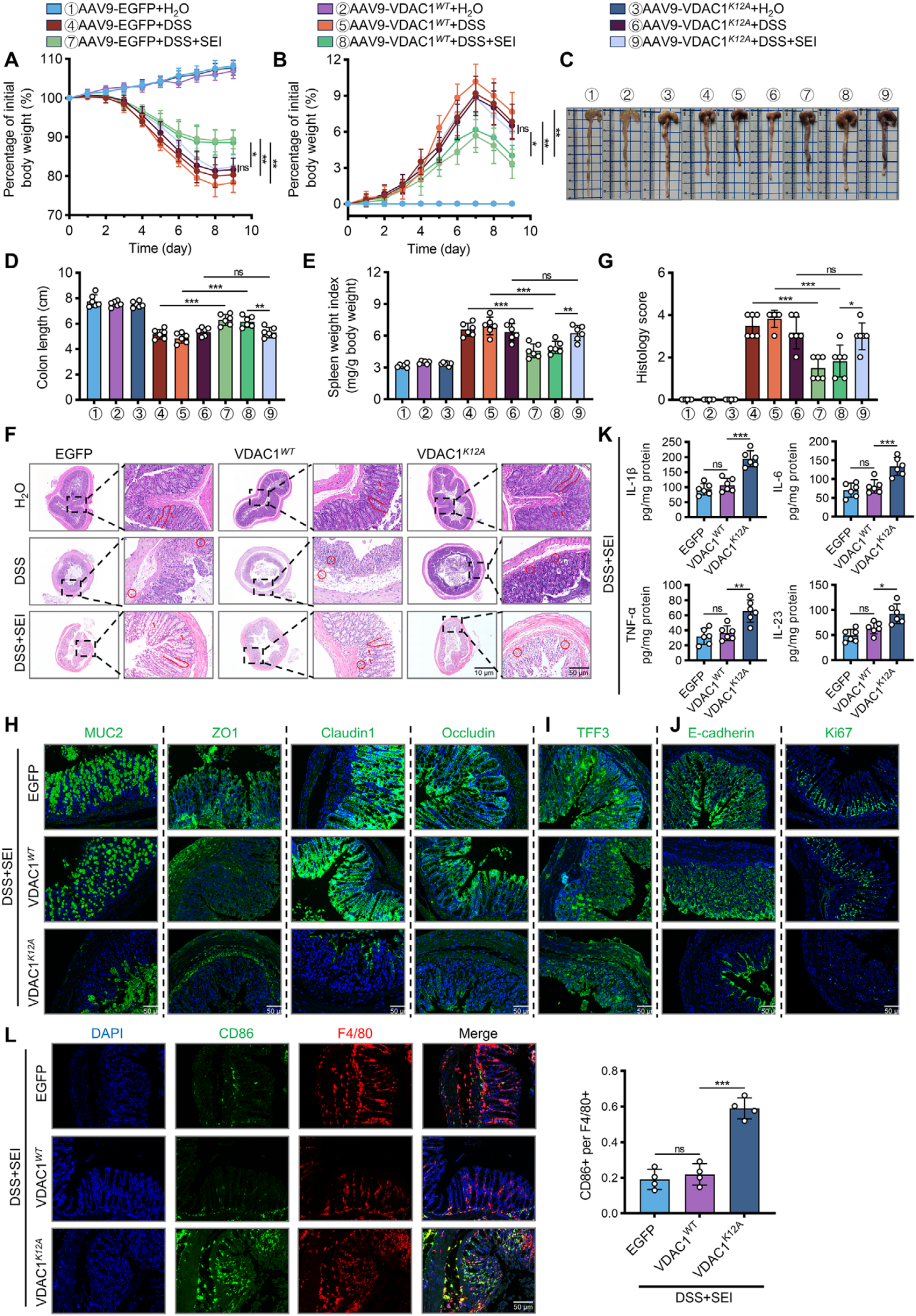
The VDAC1 K12 site is required for the protective roles of SEI in UC mice. (A) Daily assessments of body weight change and (B) DAI were conducted (*n* = 6). (C) Gross morphology images of the colon were captured on day 9 after DSS treatment, and (D) colon length was measured (*n* = 6). (E) The spleen index of mice after DSS treatment. (F) Colonic sections from mice were subjected to H&E staining (*U*‐shaped curve: *U*‐shaped crypt; arrow: goblet cell; circle: inflammatory cells), and (G) a semiquantitative histological score was assessed (*n* = 6). (H) Representative fluorescent images of MUC2, ZO1, Claudin1 and Occludin in the colonic tissues (*n* = 4). (I) Representative fluorescent images of TFF3 in the colonic tissues (*n* = 4). (J) IF staining for Ki67 and E‐cadherin in colon tissues (*n* = 4). (K) ELISA for inflammatory cytokine production in colonic tissues, including IL‐1β, IL‐6, TNF‐α, and IL‐23 (*n* = 6). (L) IF staining for F4/80 (red) and CD86 (green) in colon tissues (*n* = 4). Scale bar = 50 µm. Values were expressed as mean ± SD. *p < 0.05, **p < 0.01, ***p < 0.001.

### 2.10 VDAC1 Expression is Increased in Both Active UC and Quiescent UC and Positively Correlates With Disease Severity

Our previous findings demonstrated that VDAC1 oligomerization activates the cGAS‐STING signaling pathway, mediating the development and progression of colitis in mice. To investigate whether VDAC1 plays a similar role in UC patients and to assess its clinical relevance, we collected colonic mucosal biopsy samples from the margins of inflamed colonic tissue in UC patients. We observed significant upregulation of VDAC1 and cGAS‐STING pathway‐related proteins in UC patients compared with healthy controls (Figure 10A). Furthermore, IF analysis confirmed that STING was predominantly expressed in macrophages and showed significantly higher expression in actively inflamed UC patients compared to both healthy controls and quiescent UC patients (Figure 10B), indicating its potential involvement in UC pathogenesis. Notably, consistent with our murine model findings, VDAC1 expression in UC colonic tissues exhibited a positive correlation with the expression of cGAS‐STING‐related proteins (cGAS, STING, p‐TBK1, p‐IRF3) and with Mayo endoscopic scores, confirming the clinical relevance of the VDAC1‐cGAS‐STING axis (Figure 10C).

**FIGURE 10:**
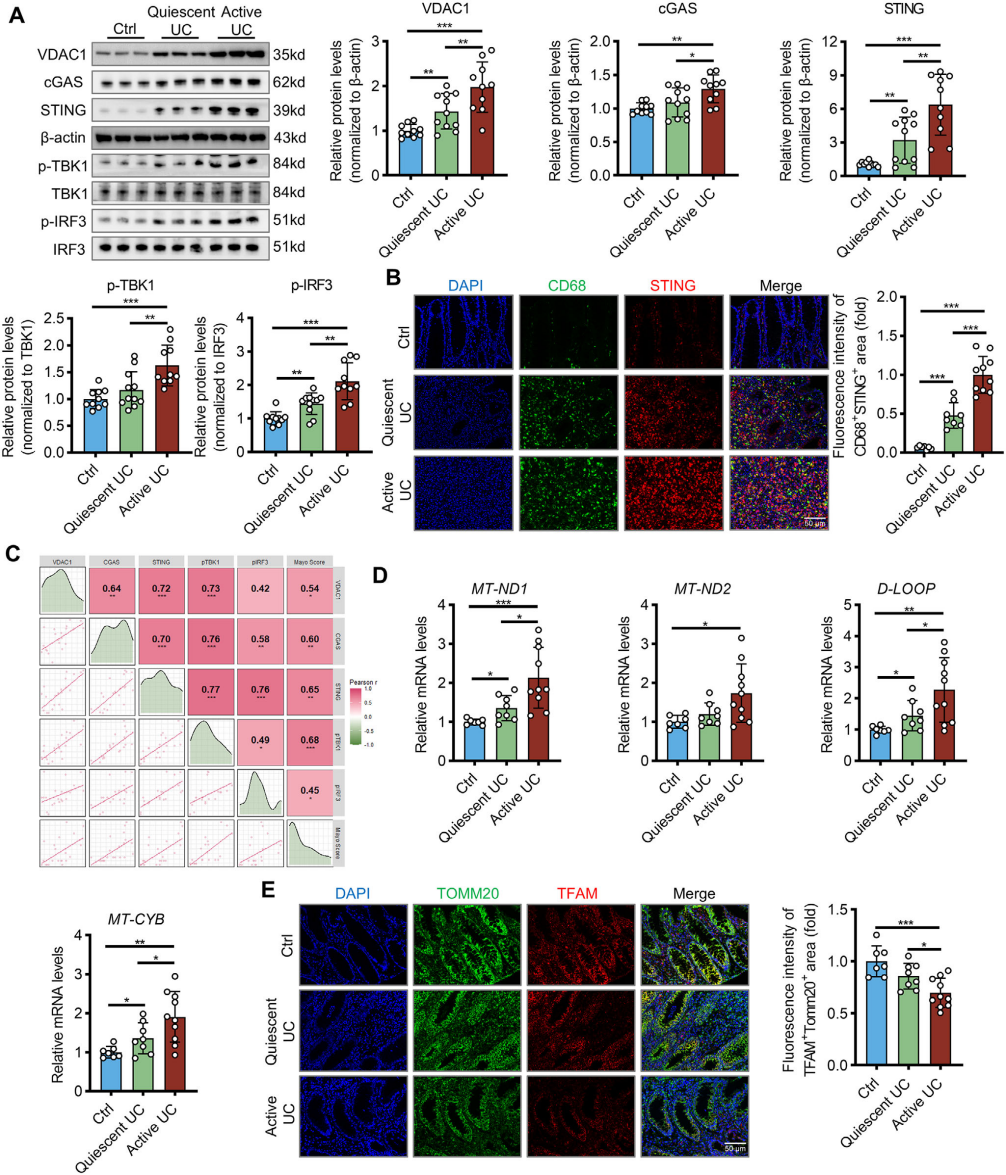
VDAC1 expression is increased in both active UC and quiescent UC and positively correlates with disease severity. (A) WB analysis of the protein levels of VDAC1, CGAS, STING, TBK1, p‐TBK1, IRF3, and p‐IRF3 in colonic tissue of healthy controls (*n* = 10), quiescent UC patients (*n* = 11), and active UC patients (*n* = 10). (B) IF staining for CD68 (green) and STING (red) in colonic tissue of healthy controls (*n* = 7), quiescent UC patients (*n* = 8) and active UC patients (*n* = 10). (C) Evaluate the association between VDAC1 and proteins associated with the cGAS‐STING pathway, as well as the severity of UC, through correlation analysis (*n* = 21). (D) The mRNA expression levels of *MT‐ND1*, *MT‐ND2*, *D‐LOOP*, and *MT‐CYB* in colonic tissue of healthy controls (*n* = 7), quiescent UC patients (*n* = 8), and active UC patients (*n* = 10) were measured by qRT‐PCR. (E) IF staining for TOMM20 (green) and TFAM (red) in colonic tissue of healthy controls (*n* = 7), quiescent UC patients (*n* = 8) and active UC patients (*n* = 10). Scale bar = 50 µm. Values were expressed as mean ± SD. *p < 0.05, **p < 0.01, ***p < 0.001.

We next examined changes in mtDNA‐related gene expression. qRT‐PCR analysis revealed that mtDNA‐related genes were significantly elevated in active UC patients compared to both healthy controls and quiescent UC patients. Importantly, expression levels in quiescent UC were also higher than those in healthy controls, indicating a graded increase corresponding to disease severity (Figure 10D). In addition, IF analysis showed that TFAM expression was significantly downregulated in active UC patients (Figure 10E).

In summary, these results demonstrate that VDAC1 expression is increased in UC and correlates positively with disease activity. The activation of the cGAS‐STING pathway, along with elevated mtDNA‐related gene expression, follows a severity‐dependent gradient across healthy controls, quiescent UC, and active UC. These findings suggest that VDAC1 oligomerization promotes mtDNA damage and dsDNA release, thereby activating the cGAS‐STING pathway in a manner closely associated with UC pathogenesis.

## 3 Discussion

In this study, we delineated a novel pharmacological pathway through which the natural compound SEI ameliorates experimental UC, with several layers of innovation. First, to our knowledge, this is the first report demonstrating the potent therapeutic efficacy of SEI in both acute and chronic models of UC. Second, we identified VDAC1 as a direct molecular target of SEI and revealed that SEI specifically binds the K12 residue to inhibit its oligomerization, a previously unreported mechanism of action for this compound in macrophages. Most importantly, in the context of UC pathogenesis, we established VDAC1 oligomerization as a critical upstream event governing mtDNA‐mediated activation of the cGAS‐STING pathway, thus linking mitochondrial outer membrane permeability to a key innate immune signaling cascade in intestinal inflammation. Our integrated approach, combining AI‐aided prediction, activity‐based proteomic profiling, and in vivo genetic validation, has provided compelling evidence that targeting the VDAC1‐cGAS‐STING axis with SEI is a promising therapeutic strategy for UC.

Dysregulation of the cGAS‐STING axis specifically within macrophages is a fundamental contributor to the pathogenesis of intestinal inflammation [47]. Clinical and experimental evidence indicates that both cGAS and STING are significantly upregulated in patients with UC during active phases and in murine colitis models, and their levels exhibit a positive correlation with disease severity [5, 53]. Aberrant activation of this pathway disrupts the epithelial barrier, facilitates macrophage infiltration and M1 polarization, and stimulates the production of a cascade of pro‐inflammatory cytokines, thus fueling a self‐perpetuating cycle of inflammation [54, 55]. Consequently, targeted inhibition of the cGAS‐STING pathway has emerged as a promising therapeutic strategy for UC. In the current study, we performed an integrative analysis of AI‐based pathway prediction and proteomics analysis, combined with functional validation, to confirm the inhibitory role of SEI on the cGAS‐STING pathway in the pathogenesis of UC, although this pathway was not ranked among the top candidates in the proteomics analysis. Current cGAS‐STING pathway inhibitor development has largely focused on downstream nodes [56, 57]. However, targeting downstream components may have limitations, including incomplete suppression from persistent upstream signaling, a narrow therapeutic window if STING inhibitors broadly disrupt its homeostatic immune functions, and compensatory activation of other inflammatory pathways [2, 58, 59]. Our study identifies VDAC1, a key regulator of the initial trigger, as a promising pharmacological target upstream of the STING pathway. Targeting cGAMP generation at this point may be more effective for controlling inflammation than inhibiting downstream STING signaling [60]. Notably, existing upstream inhibitors of the cGAS‐STING pathway mostly target the mPTP [13, 61, 62], while SEI targets VDAC1 oligomerization, which represents another critical pathway for mtDNA release. This provides a novel upstream intervention strategy for UC treatment.

Our study demonstrates that VDAC1 oligomerization is a well‐established gateway for the cytosolic release of pro‐inflammatory mitochondrial components, including mtDNA in UC. This mechanism complements the recently elucidated pathways of mtDNA release in gut inflammation, including necroptosis‐inducing mtDNA release [63] and TFAM‐bound mtDNA release [53]. These studies collectively underscore the important role of mtDNA leakage in colitic pathogenesis. The cornerstone of our discovery is the direct inhibition of VDAC1 oligomerization by SEI. By selectively plugging this leak via VDAC1, SEI effectively quenches a primary trigger for cGAS‐STING activation at its source. Notably, although the role of VDAC1 in mitochondrial permeability has been explored in UC [64], our work integrates this oligomerization process, mtDNA release, and cGAS‐STING activation into a coherent, druggable signaling axis in UC pathogenesis. Under cellular stress, the N‐terminal domain of VDAC1 undergoes translocation, thereby exposing a positively charged region where residue K12 is located [14]. This residue acts as a docking site for mtDNA. Hence, the K12 residue is not merely a passive component but an active facilitator in the series of events that connect mitochondrial damage to innate immune activation. Our finding that SEI directly interacts with K12 to inhibit this oligomerization process effectively halts the initial step in this harmful cascade, presenting a precise upstream intervention point.

The functional consequences of VDAC1 inhibition by SEI in macrophages are multifaceted. VDAC1 oligomerization‐induced mitochondrial dysfunction serves as a core upstream regulator in this pathological network: its activation triggers the release of mtDNA and ROS, which in turn activate the cGAS‐STING pathway and NLRP3 inflammasome, promote macrophage ferroptosis [65], and skew macrophage polarization toward the pro‐inflammatory M1 phenotype [66]; in turn, excessive oxidative stress and mitochondrial damage further amplify cGAS‐STING signaling, forming a positive feedback loop that aggravates intestinal mucosal inflammation, epithelial barrier dysfunction, and other UC‐related pathological injury [5]. By inhibiting VDAC1 oligomerization and reducing mitochondrial damage, SEI significantly mitigated oxidative stress by reducing excessive ROS generation. This directly attenuated the key drivers for NLRP3 inflammasome assembly, pyroptosis, and ferroptosis [67]. Our data showing SEI‐mediated suppression of NLRP3 activation, GSDMD cleavage, and normalization of ferroptosis markers are in line with the established role of the cGAS‐STING axis in promoting these cell death pathways [5]. Regarding the safety profile of SEI, our preliminary toxicological assessment indicated no observable histological alterations or signs of toxicity in major organs at the effective doses in mice. This finding provides an important preliminary safety foundation for further development. It is noteworthy that the parent herb, *L. striatum*, has a long history of clinical use in traditional medicine, which may suggest a favorable biocompatibility profile for its constituents [18, 19, 68]. However, comprehensive studies on the pharmacokinetic properties, long‐term toxicity, and possible off‐target effects of SEI in higher organisms are still necessary to advance its clinical potential.

In conclusion, we identified SEI as a novel inhibitor of VDAC1 oligomerization that targets the VDAC1‐mtDNA‐cGAS‐STING axis to alleviate colitis via a multi‐pronged mechanism in macrophages: preserving mitochondrial integrity, arresting inflammatory signaling initiation, and attenuating oxidative stress and related cell death. These findings not only highlight SEI as a promising candidate for UC therapy but also establish the VDAC1‐cGAS‐STING pathway as an innovative therapeutic target for chronic inflammatory and autoimmune diseases. Nevertheless, several limitations of this study should be noted: most mechanistic investigations were performed in a DSS‐induced acute colitis model, which cannot fully replicate the complex pathological characteristics of clinical chronic UC; additionally, given the heterogeneity and multiple origins of colonic macrophages and the lack of mature protocols to simultaneously distinguish and isolate distinct macrophage subsets in colonic tissue, we were unable to verify the subset‐specific regulatory effects of SEI. These limitations do not compromise the core conclusions of this work, but point to important directions for future research, in which more advanced experimental approaches will be applied to further clarify the cell‐specific regulatory mechanisms of SEI against UC.

## 4 Experimental Section

### 4.1 Reagents and Antibodies

Dextran sulfate sodium salt (molecular weight [MW]: 36–50 kDa) (SKU: 0216011090) was purchased from MP Biomedicals (California, USA). Senkyunolide I (HY‐N0745), Erastin (HY‐15763), H‐151 (HY‐112693), TNF‐α (HY‐P7058 or HY‐P7090), IFN‐γ (HY‐P7025 or HY‐P7071), Lipopolysaccharides (LPS, HY‐D1056), Adenosine 5'‐triphosphate (ATP, HY‐B2176) were purchased from MedChem Express. TSA Plus Fluorescent Double‐Label Three‐Color Staining Kit was purchased from Servicebio (Wuhan, China). Detailed antibody information is provided in Table S2.

### 4.2 Ethics

This study was conducted in accordance with the Declaration of Helsinki and approved by the Ethics Committee of Zhuhai Hospital Affiliated with Jinan University (Approval No. 2023[108]). Written informed consent was obtained from all participants. Colonic mucosal biopsies were collected from 21 UC patients and 10 healthy donors at the same hospital. Diagnosis and disease activity assessment followed the Chinese Consensus on Diagnosis and Treatment of Inflammatory Bowel Disease (Beijing, 2018) [69]. Patient demographics are detailed in Table S3.

All animal procedures complied with the NIH Guide for the Care and Use of Laboratory Animals and were approved by the Institutional Animal Care Committee of Guangdong Pharmaceutical University (Approval No. gdpulac2022794).

### 4.3 Animal Protocol

Male C57BL/6 mice were purchased from Guangdong Medical Laboratory Animal Center (Guangdong, China). All mice were housed at a constant room temperature with a 12/12 h light–dark cycle and fed with a standard rodent diet and water in the Animal Centre of Guangdong Pharmaceutical University.

1. To evaluate the effect of SEI on UC mice, chemically induced acute colitis was established as reported previously [70]. Mice were randomly divided into six groups: control group (H_2_O), untreated colitis group, colitis + low‐dose SEI group (12.5 mg/kg/day) [71], colitis + middle‐dose SEI group (25 mg/kg/day), colitis + high‐dose SEI group (50 mg/kg/day) and colitis + 5‐ASA (100 mg/kg/day) group [72]. Colitis was induced by administering 3% (*w/v*) DSS in drinking water for 5 days, followed by a switch to normal drinking water for an additional 4 days. SEI and 5‐ASA were dissolved in saline and administered to the indicated groups by gavage once daily for the duration of the 9‐day experiment. The control and untreated colitis groups received an equivalent volume of saline. To construct a chronic colitis model in mice, DSS at a concentration of 2% (*w/v*) was given ad libitum for 7 days, followed by normal drinking water for 14 days. The cycle was repeated three times (7 days of DSS, 14 days of water).
2. To evaluate whether VDAC1 K12 participates in SEI‐mediated protection against UC, wild‐type mice were infected with AAV9 vectors expressing VDAC1 WT (AAV9‐VDAC1^WT^) or VDAC1 K12A (AAV9‐VDAC1^K12A^), or with an empty vector (AAV9‐EGFP). The mouse VDAC1 gene was cloned into AAV9 by Genechem Co., Ltd (Shanghai, China). This model utilized a total of 54 mice, with each experimental group comprising 6 mice. Each mouse received intraperitoneal injections of AAV9 virus at a dose of 5 × 10^11^ vg. Four weeks after injection, the UC mouse model was established, and different treatments were given according to the group.
3. To further verify that the protective effect of SEI is mediated through the STING signaling pathway, mice were randomly divided into five groups (*n* = 6 per group): control group (H_2_O), DSS group, DSS + SEI group (50 mg/kg/day), DSS + H‐151 group (10 mg/kg), and DSS + SEI + H‐151 group. Colitis was induced by administering 3% (*w/v*) DSS in drinking water for 5 days, followed by a switch to normal drinking water for an additional 4 days. SEI was dissolved in saline and administered daily by oral gavage at 50 mg/kg body weight. H‐151 was administered intraperitoneally at 10 mg/kg body weight every other day for 9 days, starting from day 0 [73].
4. To investigate whether SEI exerts its anti‐UC effect by inhibiting ferroptosis, mice were randomly divided into four groups (*n* = 6 per group): control group (H_2_O), DSS group, DSS + SEI group (50 mg/kg/day), and DSS + SEI + Erastin group. Colitis was induced by administering 3% (*w/v*) DSS in drinking water for 5 days, followed by a switch to normal drinking water for an additional 4 days. SEI was dissolved in saline and administered daily by oral gavage at 50 mg/kg body weight. Erastin was administered intraperitoneally at 30 mg/kg body weight every other day for 9 days, starting from day 0 [74].
5. To evaluate the in vivo safety of SEI, mice were randomly divided into four groups: a control group (receiving saline only), a low‐dose SEI group (12.5 mg/kg/day), a medium‐dose SEI group (25 mg/kg/day), and a high‐dose SEI group (50 mg/kg/day). SEI was dissolved in saline and administered daily by oral gavage to the respective treatment groups over a 9‐day experimental period, while the control animals received an equal volume of saline. After the experiment, tissue samples including the heart, liver, spleen, kidney, and colon were collected from all mice for pathological analysis.

All mice were monitored daily. DAI scores were calculated based on established criteria [75]. Intestinal permeability was measured by oral administration of FITC‐dextran (MW 4000) at a dose of 600 mg/kg of body weight.

### 4.4 AI Model Construction and Implementation

The AI model for target prediction was developed by strictly following the methodology described by Liu et al. [76]. Briefly, a GDOP model was built by integrating compound‐induced gene expression signatures from the LINCS L1000 database with a human protein–protein interaction network. The model unified these biological profiles with chemical structure fingerprints (Morgan fingerprints) via a deep neural network to predict potential “on‐target” pathways for a given compound. This pre‐trained model was then applied to SEI. The gene expression profile and structural information of SEI were processed and input into the model, which generated a ranked prediction of its activity across curated biological pathways. The top predicted pathways guided our subsequent hypothesis and experimental investigation into its mechanism of action in colitis.

### 4.5 Cell Lines

THP‐1 cells (iCell‐h213), HEK293T cells (iCell‐h237), and iBMDM cells (iCell‐0060a) were purchased from iCell Bioscience Inc. (Shanghai, China). These cell lines were cultured in RPMI‐1640 or DMEM media, supplemented with 10% (*v/v*) fetal bovine serum and 1% (*v/v*) penicillin‐streptomycin, and maintained at 37°C in a humidified atmosphere with 5% CO_2_. THP‐1‐derived macrophages were induced from THP‐1 cells under the stimulation of phorbol 12‐myristate 13‐acetate (PMA, 100 ng/mL) for 24 h.

### 4.6 Histological Analysis and IF

Colon samples designated for histological analysis were fixed in 4% paraformaldehyde at room temperature and embedded in paraffin. Thin sections were prepared and stained with Hematoxylin and Eosin (H&E). IF staining was performed using a TSA Plus Fluorescent Double‐Label Three‐Color Staining Kit. Following deparaffinization and antigen retrieval, slides were subjected to multiplex fluorescent immunohistochemistry using the TSA Fluorescence Double Staining Kit. For the observation of ultrastructural changes using TEM analysis, the colon tissues were sliced into 1 mm^3^ tissue blocks and fixed with an electron microscope fixative containing 2.5% glutaraldehyde. After undergoing dehydration with ethanol, the samples were embedded in Durcupan resin to prepare ultra‐thin sections for TEM analysis.

To image SEI‐P, iBMDM cells were seeded into sterile glass‐bottom dishes and stimulated with TNF‐α plus IFN‐γ for 3 h. Cells were then placed in medium containing 100 µm SEI‐P and incubated for 0, 0.5, 1, 2, or 4 h as specified. Cells were fixed with 4% paraformaldehyde, incubated with DAPI dye, washed twice with PBS, and finally scanned using confocal microscopy.

### 4.7 VDAC1 Cross‐Linking Assay

To stabilize and detect VDAC1 oligomers, a chemical cross‐linking assay was performed in intact iBMDM cells using the membrane‐permeable cross‐linker EGS (ethylene glycol bis (succinimidyl succinate); Thermo Fisher). The assay was performed based on established protocols with modifications suitable for our experimental system [49]. In brief, harvested cells were washed with cold PBS (pH 7.4) and incubated with 0.5 mm EGS in PBS at 30°C for 20 min. The reaction was quenched with Tris‐HCl (pH 7.8) to a final concentration of 20 mm. After centrifugation, the cell pellet was lysed, and protein concentration was determined by BCA assay. Proteins (50 µg per lane) were separated by 10% SDS‐PAGE and analyzed by immunoblotting using an anti‐VDAC1 antibody.

### 4.8 In‐Gel Fluorescence Labeling of Proteome

In‐gel fluorescence labeling of the proteome was performed as previously described [48, 77]. iBMDM macrophages were primed with TNF‐α plus IFN‐γ for 3 h, followed by treatment with SEI‐P or vehicle (DMSO) for 2 h. Total proteins were extracted and quantified. Equal protein aliquots were incubated for 2 h at room temperature with a click chemistry cocktail containing 50 µmol/L Cy3‐azide, 100 µmol/L THPTA, 1 mmol/L sodium ascorbate, and 1 mmol/L CuSO_4_. Proteins were precipitated with ice‐cold acetone, dissolved in SDS loading buffer, and denatured. After separation by 10% SDS‐PAGE, SEI‐P‐labeled proteins were visualized using a ChemiDoc MP imaging system. Total protein levels were assessed by Coomassie Brilliant Blue staining.

### 4.9 Competitive Labeling Experiments

For competition assays, cell lysates were pre‐incubated with the competitor SEI for 1 h prior to labeling with SEI‐P for 2 h. Subsequent click chemistry conjugation, protein precipitation, SDS‐PAGE separation, and fluorescence/Coomassie staining were performed identically to the procedures described in the “In‐gel Fluorescence Labeling” section above.

### 4.10 Target Pull‐Down and LC‐MS/MS Identification

To identify SEI‐P‐binding targets, iBMDM cells were activated with TNF‐α plus IFN‐γ for 3 h, then treated with SEI (competition group) or vehicle (probe‐labeled group) for 1 h, followed by incubation with SEI‐P for 2 h. Total protein was subjected to click chemistry using a cocktail containing Biotin‐N3. Proteins were precipitated, resuspended in PBS containing 1.5% SDS, and incubated with streptavidin beads overnight at 4°C. Beads were washed stringently, and bound proteins were eluted, separated by SDS‐PAGE, and processed for in‐gel tryptic digestion. Peptides were desalted, labeled with TMT reagents, and analyzed by LC‐MS/MS on an Orbitrap Astral Mass Spectrometer (Thermo Scientific) [78]. Enriched proteins were selected based on significant abundance changes (fold change <−1, *p* < 0.05) between competition and probe‐only groups.

### 4.11 Statistical Analysis

All statistical analyses and graphical presentations were completed with GraphPad Prism (Ver. 9.4.1). Continuous data were summarized as mean ± SD from a minimum of three independent experimental repeats. Comparisons between two groups were performed using the unpaired two‐tailed Student's *t*‐test. For multi‐group comparisons not involving repeated measurements, one‐way analysis of variance (ANOVA) was used, followed by Tukey's multiple comparison test or Dunnett's test as appropriate. For repeated‐measures data, such as body weight and DAI score collected over time, two‐way repeated‐measures ANOVA was used. When significant main effects or interactions were detected, post‐hoc comparisons were performed using Bonferroni‐corrected *t‐*tests. Bivariate correlations were assessed using Pearson's correlation coefficient (two‐tailed). All statistical tests were two‐tailed, and a *p*‐value < 0.05 was considered statistically significant.

## Author Contributions

B.L., Y.J., Z.X., and C.Z. supervised the study and designed the research. Z.Y., Y.H., B.H., L.Z., C.Y., and Y.Y. performed the experiments. Z.Y. and C.Z prepared the manuscript. Y.Y. helped with clinical data collection. Y.H. and B.H. helped with data analysis. All authors read, revised, and approved the final manuscript.

## Conflicts of Interest

The authors declare no conflicts of interest.

## Supporting information




**Supporting File**: advs77045‐sup‐0001‐SuppMat.docx.

## Data Availability

The data that support the findings of this study are available from the corresponding author upon reasonable request.
