# Supplementary material for: Senkyunolide I Inhibits mtDNA‐cGAS‐STING Signaling in Macrophages via Targeting VDAC1 Oligomerization to Attenuate Ulcerative Colitis
Source: Adv Sci (Weinh). 2026 Aug 7;13(60):e77045. doi: 10.1002/advs.77045 (PMC13449549; doi:10.1002/advs.77045)
Supplement: Supplementary file 1 [file ADVS-13-e77045-s001.docx]

**Supporting Information for**

**Original article**

**Senkyunolide I Inhibits** **mtDNA-cGAS-STING Signaling in Macrophages via Targeting VDAC1 Oligomerization to Attenuate Ulcerative Colitis**

*Zhiming Ye, Yihang Huang, Bohao Han, Lei Zhang, Can Yu, Yang Yang, Bing Liu*, Yongping Jian*, Zhixiang Xu*, Cheng Zeng**

Zhiming Ye, Yihang Huang, Bohao Han, Can Yu, Cheng Zeng

Center for Drug Research and Development, Guangdong Pharmaceutical University, Guangzhou, Guangdong, China.

Zhiming Ye

Key Laboratory of Pathobiology, Ministry of Education, Jilin University, Changchun, Jilin, China.

Lei Zhang

Department of Hepatobiliary-Pancreatic and Hernia Surgery, The Affiliated Guangdong Second Provincial General Hospital of Jinan University, Guangzhou, Guangdong, China.

Yang Yang

Department of Pharmacy, Zhuhai People’s Hospital (Zhuhai Hospital Affiliated with Jinan University), Zhuhai, China.

Bing Liu

College of Pharmacy, Guangdong Pharmaceutical University, Guangzhou, China.

E-mails: liubing520@gdpu.edu.cn

Yongping Jian

School of Life Sciences, Henan University, Kaifeng, Henan, China.

E-mails: yongpingjian123@163.com

Zhixiang Xu

Key Laboratory of Pathobiology, Ministry of Education, Jilin University, Changchun, Jilin, China; Department of Urology, The First Hospital of Jilin University, Changchun, Jilin, China; School of Life Sciences, Henan University, Kaifeng, Henan, China.

E-mails: zhixiangxu@jlu.edu.cn

Cheng Zeng

Key Specialty of Clinical Pharmacy, The First Affiliated Hospital of Guangdong Pharmaceutical University, Guangzhou, Guangdong, China.

E-mails: [zengcheng@gdpu.edu.cn](mailto:zengcheng@gdpu.edu.cn)

**Supplemental Methods**

*Cell Viability Assay:* Cell viability was evaluated using the CCK-8 assay. iBMDM cells were seeded into 96-well plates separately and incubated in a 37 °C incubator with 5% CO_2_ overnight. The following day, cells were exposed to different concentrations of SEI. After 24 h, 10 µL of CCK-8 solution was added to each well and incubated for 1 h before measuring absorbance at 450 nm.

*WB Analysis:* Tissues and cells were lysed with RIPA lysate (Thermo Scientific) containing protease and phosphatase inhibitors and analyzed by WB using standard methods, and PVDF membranes were incubated with specific antibodies.

*Molecular Docking and Molecular Dynamics Simulations:* The structure of SEI was downloaded from PubChem, and the three-dimensional structure of VDAC1 (PDB: 2JK4) was obtained from the RCSB PDB. Discovery Studio was used to add water and hydrogen atoms to the VDAC1 protein. SEI was docked onto VDAC1 using AutoDock Vina. The docking results were analyzed and visualized using PyMOL software (version 2.2). To assess the binding stability between SEI and VDAC1 and to calculate their binding free energies, we performed 100 ns molecular dynamics simulations using the GROMACS software package (https://www.gromacs.org/).

*RNA isolation and qRT-PCR:* Total RNA was isolated from tissues and cells using TRIzol Reagent (Invitrogen). Reverse transcription was conducted to generate cDNA libraries utilizing the ABScript III RT Master Mix for qPCR with gDNA Remover (ABclonal, Wuhan, China). qRT-PCR was carried out with the SYBR Green Fast qPCR Mix (ABclonal, Wuhan, China) on an iCycler (Bio-Rad) according to the manufacturer's instructions. The primers utilized in qRT-PCR are listed in Supporting Information Table S4.

*ELISA and Biochemical Assay:* The levels of IL-1β, IL-6, TNF-α, and IL-23 in colonic tissues were quantified using commercially available ELISA kits (MultiSciences Biotech, Shanghai, China). Intestinal concentrations of MDA, SOD, CAT, GPX, and GSH were determined according to the manufacturer's guidelines using commercial kits (Nanjing Jiancheng Bioengineering Institute, China).

*Measurement of mtDNA:* Cytosolic fractions were prepared using digitonin‑based permeabilization (25 μg/mL digitonin in 150 mM NaCl, 50 mM HEPES, pH 7.4) followed by sequential centrifugation at 2,000 × g and 20,000 × g to remove nuclei and mitochondria. DNA was extracted from the final supernatant and subjected to qPCR using primers specific for mitochondrial genes *mt‑Nd1*, *D‑loop*, and *mt‑Cytb*, with *18S rRNA* as a nuclear control to exclude contamination. Relative cytosolic mtDNA levels were calculated using the 2⁻^ΔΔCt^ method and normalized to 18S rRNA.

*Mitochondrial Morphology Analysis:* For mitochondrial morphology analysis, after the indicated treatments, cells were incubated with 1 μM MitoTracker Red CMXRos (Molecular Probes, Invitrogen, USA) for 30 min at 37 °C (1). The structure of mitochondria was viewed by confocal scanning microscopy (LSM980, Zeiss, Germany).

*Isolation and primary culture of* *colonic tissue-derived macrophages and peritoneal macrophages:* For colonic macrophages, the mouse colon was removed, flushed with ice‑cold PBS, cut longitudinally, and minced into small pieces. The tissue was subjected to three 20‑min incubations in HBSS containing 2 mM EDTA at 37 °C with gentle agitation to remove the epithelial layer. The remaining tissue was then digested in RPMI‑1640 containing 1.25 mg/mL collagenase D, 1 mg/mL dispase II, and 0.1 mg/mL DNase I for 30-35 min at 37 °C. The resulting cell suspension was filtered through a 70 μm strainer, and colonic macrophages were isolated by positive selection using anti‑F4/80 magnetic beads (100-0659, STEMCELL Technologies) according to the manufacturer’s instructions.

For peritoneal macrophages, mice were injected intraperitoneally with 2 mL of sterile 3% Brewer’s thioglycollate broth four days before harvest. The peritoneal cavity was lavaged with 8-10 mL of ice‑cold PBS, and the recovered fluid was centrifuged at 400 × g for 10 min. Peritoneal macrophages were then purified using anti‑F4/80 magnetic beads as above.

Both colonic tissue-derived and peritoneal macrophages were seeded in culture plates and incubated at 37 °C in a 5% CO₂ incubator for downstream functional assays.

*Flow cytometry*: Colonic total mononuclear cells were isolated based on a previously described protocol with modifications (2). Briefly, the entire colon was removed, flushed with ice-cold PBS, and minced into small pieces. The tissue was digested with collagenase D (0.5 mg/mL), DNase I (0.5 mg/mL), and dispase II (3 mg/mL) in PBS for 20 min at 37 °C under slow rotation. The digestion was repeated 2–3 times, and the resulting cell suspension was filtered through a 40‑μm cell strainer, washed, and used directly for flow cytometry. The cells were harvested and stained with fluorescently labeled monoclonal antibodies according to the manufacturer’s instructions. Lamina propria M1 and M2 macrophages were identified as CD206^-^ CD86^+^ and CD206^+^ CD86^-^ cells, respectively. Finally, the M1 and M2 polarization of the macrophages was analyzed by flow cytometry (Attune NxT, Thermo Fisher Scientific).

*Plasmid Construction and Cell Transfection:* VDAC1-deficient iBMDM cells were generated by CRISPR/Cas9 technique (VDAC1 Mouse Gene Knockout Kit, Origene, KN518873). Complete knockout of VDAC1 was verified by WB analysis. Expression vectors encoding Flag-tagged VDAC1 mutants with K12A, K174A and G172A substitutions were generated by QuikChange Lightning Multi Site-Directed Mutagenesis Kit (Agilent Technologies, 210516) following the experimental method described in our previous report (3). For transfection of plasmids into HEK293T and iBMDM cells, Lipofectamine 3000 (Invitrogen, L3000015) was used according to the manufacturer's instructions.

*CETSA:* iBMDM were incubated with or without SEI (100 μM) for 24 h. Subsequently, the cells were collected and subjected to the CETSA as described previously (4). In brief, the incubated cells were divided into 11 equal parts. Each part underwent a 3-min heat treatment at different temperatures (37, 40, 43, 46, 49, 52, 55, 58, 61, 64, and 67 °C). The heated cells were then frozen at -80 °C for 12 h, followed by a 5-min incubation at room temperature, and this process was repeated once more. Afterward, cell lysates were obtained through centrifugation at 20,000 × g for 20 min, and the levels of VDAC1 were assessed using WB analysis.

*DARTS:* The extracts derived from iBMDM cells were incubated either with or without SEI (100 μM), following the procedures as described previously (4). Incubation was performed for 24 h at room temperature. Subsequently, the lysates were divided into six distinct portions to undergo digestion, using various concentrations of pronase E (Sigma-Aldrich). Each digestion lasted for 20 min at room temperature. Upon the addition of loading buffer to stop the digestion, the samples were promptly boiled. These treated samples were individually loaded onto 10% SDS-PAGE for subsequent WB analysis.

**References**

1.	Zeng C, Duan F, Hu J, Luo B, Huang B, Lou X, Sun X, Li H, Zhang X, Yin S, Tan H. NLRP3 inflammasome-mediated pyroptosis contributes to the pathogenesis of non-ischemic dilated cardiomyopathy. Redox Biol. 2020;34:101523.

2.	Weigmann B, Tubbe I, Seidel D, Nicolaev A, Becker C, Neurath MF. Isolation and subsequent analysis of murine lamina propria mononuclear cells from colonic tissue. Nat Protoc. 2007;2(10):2307-2311.

3.	Ye Z, Deng M, Yang Y, Song Y, Weng L, Qi W, Ding P, Huang Y, Yu C, Wang Y, Wu Y, Zhang Y, Yuan S, Nie W, Zhang L, Zeng C. Epithelial mitochondrial fission-mediated PANoptosis is crucial for ulcerative colitis and its inhibition by saquinavir through Drp1. Pharmacol Res. 2024;210:107538.

4.	Jafari R, Almqvist H, Axelsson H, Ignatushchenko M, Lundback T, Nordlund P, Martinez Molina D. The cellular thermal shift assay for evaluating drug target interactions in cells. Nat Protoc. 2014;9(9):2100-2122.

**Supplemental Figures and Tables**

**Figure S1.** **SEI relieves intestinal barrier damage in colitis mice.** (A) Serum fluorescence intensity was measured in mice with chronic UC models following injection of FITC-dextran (n = 6). (B-C) Representative fluorescent images of MUC2, Occludin, and Claudin-1 in the colonic tissues and their relative quantification from a chronic UC mouse model (n = 4). Values were expressed as mean ± SD. ^###^*P* < 0.001 *vs.* H_2_O group; **P* < 0.05, ***P* < 0.01, ****P* < 0.001 *vs.* DSS group.

**Figure S2.** **SEI inhibits DSS-induced colonic inflammation and suppresses M1 polarization of macrophages.** (A) qRT-PCR analysis of inflammatory cytokines (*IL-1β*, *IL-6*, *TNF-α* and *IL-23*) in colonic tissue from a chronic UC mouse model (n = 6). (B) ELISA for inflammatory cytokine production in colonic tissue from a chronic UC mouse model, including IL-1β, IL-6, TNF-α and IL-23 (n = 6). (C-D) IF staining for F4/80 (red) and CD86 (green) in colonic tissue and their relative quantification from a chronic UC mouse model (n = 4, ^###^*P* < 0.001 *vs.* H_2_O group; **P* < 0.05, ***P* < 0.01, ****P* < 0.001 *vs.* DSS group). (E) The cytotoxicity of SEI on iBMDM cells was assessed by CCK-8 assay. Values were expressed as mean ± SD (n = 3). **P* < 0.05, ***P* < 0.01, ****P* < 0.001 *vs.* SEI (0 μM) group. (F) The mRNA expression levels of *IL-1β*, *IL-6*, *TNF-α* and *IL-23* were measured by qRT-PCR (n = 3). (G) The mRNA expression levels of *iNOS* and *CD86* were measured by qRT-PCR (n = 3). Values were expressed as mean ± SD. **P* < 0.05, ***P* < 0.01, ****P* < 0.001.

**Figure S3.** **SEI inhibits the activation of mtDNA-cGAS-STING inflammatory** **signaling pathway.** (A) The protein expression of cGAS, STING, p-TBK1 and p-IRF3 in the colonic tissues of mice was determined by WB (n = 6). (B) IF staining for cGAS, p-TBK1, and p-IRF3 in colonic tissue and their relative quantification (n = 4). (C) The mRNA expression levels of *IFNB*, *CCL5*, *CXCL10*, *IFIT1* and *ISG15* were measured by qRT-PCR (n = 6). (D) IF staining for F4/80 and STING in colonic tissue and their relative quantification (n = 4). (E) IF staining for TFAM and Tomm20 in colonic tissue and their relative quantification (n = 4). ^##^*P* < 0.01, ^###^*P* < 0.001 *vs.* H_2_O group; **P* < 0.05, ***P* < 0.01, ****P* < 0.001 *vs.* DSS group.

**Figure S4.** **SEI inhibits the activation of mtDNA-cGAS-STING inflammatory** **signaling pathway** **in macrophages.** WB analysis of the phosphorylation levels of key proteins in the cGAS‐STING pathway and their relative quantification in iBMDM cells (A-B) and THP‐1 cells (C-D) co‐treated with SEI and TNF-α plus IFN-γ for 24 h. The mRNA expression levels of *IFNB*, *CCL5*, *CXCL10*, *ISG15* and *IFIT1* in iBMDM cells (E-I) and THP‐1 cells (J-N) co‐treated with SEI and TNF-α plus IFN-γ for 24 h were measured by qRT-PCR. (O) qRT-PCR analysis of cytoplasmic mtDNA (*mt-Nd1*, *D-loop* and *mt-Cytb*) level. WB analysis of the phosphorylation levels of key proteins in the cGAS‐STING pathway and their relative quantification in colonic tissue-derived macrophages (P-Q) and peritoneal macrophages (R-S) co‐treated with SEI and TNF-α plus IFN-γ for 24 h. Values were expressed as mean ± SD (n = 3). **P* < 0.05, ***P* < 0.01, ****P* < 0.001.

**Figure S5. Pharmacological inhibition of STING recapitulates the protective effect of SEI in DSS-induced colitis.** (A) Daily assessments of body weight change and (B) DAI were conducted (n = 6). (C) Gross morphology images of the colon were captured on day 9 after DSS treatment, and (D) colon length was measured (n = 6). (E) The spleen index of mice after DSS treatment was calculated. (F) Colonic sections from mice were subjected to H&E staining (U-shaped curve: U-shaped crypt; arrow: goblet cell; circle: inflammatory cells), and (G) a semiquantitative histological score was assessed (n = 6). Values were expressed as mean ± SD (n = 6). **P* < 0.05, ***P* < 0.01, ****P* < 0.001.

**Figure S6.** **VDAC1 overexpression is constructed in iBMDM cells.** (A) WB and quantitative analysis confirmed that VDAC1 protein levels were significantly increased in VDAC1-overexpressing iBMDM cells compared with empty vector (EV)-transfected cells. Values were expressed as mean ± SD (n = 3). ****P* < 0.001.

**Figure S7.** **SEI directly binds to K12 on VDAC1.** (A) The iBMDM cells were transfected with K174A, K12A and G172A mutant plasmids and then treated with DMSO or SEI (100 μM) for 1 h. The interaction between SEI and VDAC1 was detected using CETSA assay. Values were expressed as mean ± SD (n = 3). **P* < 0.05, ***P* < 0.01, ****P* < 0.001 vs. Ctrl group. (B) WB analysis of VDAC1 expression in WT and VDAC1^-/-^ iBMDM cells. Values were expressed as mean ± SD (n = 3). ****P* < 0.001. VDAC1^-/-^ iBMDM cells were transfected with an empty vector, Flag-VDAC1-WT, Flag-VDAC1(K174A), Flag-VDAC1(K12A), or Flag-VDAC1(G172A). (C) WB analyses of the expression levels of p-TBK1 and p-IRF3 and (D) qRT-PCR analyses of the mRNA expression levels of *CCL5* and *CXCL10* after stimulation with TNF-α plus IFN-γ and treatment with or without SEI (100 μM) for 24 h. Values were expressed as mean ± SD (n = 3). **P* < 0.05, ***P* < 0.01, ****P* < 0.001.

**Figure S8.** **SEI alleviates mitochondrial damage-induced oxidative stress and reduces cell pyroptosis in macrophages.** (A) Representative confocal microscopic images of MitoTracker Red. (B) IF detection of JC-1 in iBMDM cells. (C) WB analysis of the protein levels of NLRP3, CASP1, GSDMD and IL-18 in iBMDM cells primed with LPS (100 ng/mL) for 8 h followed by stimulation with 5 mM ATP for 30 min. Values were expressed as mean ± SD (n = 3). (D-E) Production of inflammatory cytokines, including IL-1β (D) and IL-18 (E), was detected by ELISA in iBMDM cells primed with LPS (100 ng/mL) for 8 h followed by stimulation with 5 mM ATP for 30 min. (F) Protective effect of SEI on TNF-α plus IFN-γ-induced cell death in iBMDM cells. (G-J) MDA content, SOD antioxidant enzyme activity, GSH content, and iron levels were measured in iBMDM cells. Values were expressed as mean ± SD (n = 3). **P* < 0.05, ***P* < 0.01, ****P* < 0.001.

**Figure S9. Pharmacological activation of ferroptosis by Erastin reverses the protective effect of SEI in DSS-induced colitis.** (A) Daily assessments of body weight change and (B) DAI were conducted (n = 6). (C) Gross morphology images of the colon were captured on day 9 after DSS treatment, and (D) colon length was measured (n = 6). (E) The spleen index of mice after DSS treatment. (F) Colonic sections from mice were subjected to H&E staining (U-shaped curve: U-shaped crypt; arrow: goblet cell; circle: inflammatory cells), and (G) a semiquantitative histological score was assessed (n = 6). Values were expressed as mean ± SD (n = 6). **P* < 0.05, ***P* < 0.01, ****P* < 0.001.

**Figure S10.** **The VDAC1 K12 site is required for the protective roles of SEI in UC mice.** (A) Representative fluorescence pictures show the infection efficiency of AAV9 in mouse colon detected by the green fluorescence of co-expressed EGFP at four weeks after intraperitoneal viral injection. (B) Representative WB and quantitative data for VDAC1 protein level at four weeks after intraperitoneal viral injection (n = 6). (C) Quantitative analysis of MUC2, ZO1, Claudin1, Occludin and TFF3 protein (n = 4). (D) Quantitative analysis of Ki67 and E-cadherin protein (n = 4). (E) The mRNA expression levels of *IL-1β*, *IL-6*, *TNF-α* and *IL-23* were measured by qRT-PCR (n = 6). Values were expressed as mean ± SD. **P* < 0.05, ***P* < 0.01, ****P* < 0.001.

**Figure S11.** **SEI exerts little toxicity on the mice.** (A) The body weight changes (n = 6). (B) The colon length (n = 6). (C) The pathological changes of heart, liver, spleen, kidney and colon were detected by H&E staining.

**Table S1 The potential “on-target” signaling pathways of Senkyunolide I predicted by GDOP.**

|  | pathway | score |
| --- | --- | --- |
| 1 | REACTOME_SIGNALING_BY_FGFR_IN_DISEASE | 0.922 |
| 2 | REACTOME_PI_3K_CASCADE_FGFR2 | 0.886 |
| 3 | REACTOME_PI_3K_CASCADE_FGFR1 | 0.829 |
| 4 | REACTOME_CYTOSOLIC_SENSORS_OF_PATHOGEN_ASSOCIATED_DNA | 0.773 |
| 5 | REACTOME_FGFR1_MUTANT_RECEPTOR_ACTIVATION | 0.765 |
| 6 | REACTOME_SUMOYLATION_OF_DNA_REPLICATION_PROTEINS | 0.747 |
| 7 | REACTOME_PROLONGED_ERK_ACTIVATION_EVENTS | 0.725 |
| 8 | REACTOME_MTORC1_MEDIATED_SIGNALLING | 0.716 |
| 9 | REACTOME_STING_MEDIATED_INDUCTION_OF_HOST_IMMUNE_RESPONSES | 0.711 |
| 10 | REACTOME_ERBB2_REGULATES_CELL_MOTILITY | 0.707 |
| 11 | REACTOME_SIGNALING_BY_FGFR1_IN_DISEASE | 0.707 |
| 12 | REACTOME_SIGNALING_BY_FGFR2_IN_DISEASE | 0.698 |
| 13 | REACTOME_PI_3K_CASCADE_FGFR4 | 0.691 |
| 14 | REACTOME_FGFR2_MUTANT_RECEPTOR_ACTIVATION | 0.671 |
| 15 | REACTOME_DOWNSTREAM_SIGNALING_OF_ACTIVATED_FGFR1 | 0.664 |
| 16 | REACTOME_NEGATIVE_REGULATION_OF_MAPK_PATHWAY | 0.663 |
| 17 | REACTOME_DOWNSTREAM_SIGNALING_OF_ACTIVATED_FGFR2 | 0.644 |
| 18 | REACTOME_RND3_GTPASE_CYCLE | 0.626 |
| 19 | REACTOME_NON_INTEGRIN_MEMBRANE_ECM_INTERACTIONS | 0.623 |
| 20 | REACTOME_ACTIVATION_OF_THE_AP_1_FAMILY_OF_TRANSCRIPTION_FACTORS | 0.595 |
| 21 | REACTOME_NEGATIVE_REGULATION_OF_FGFR2_SIGNALING | 0.593 |
| 22 | REACTOME_NOD1_2_SIGNALING_PATHWAY | 0.593 |
| 23 | REACTOME_RND2_GTPASE_CYCLE | 0.589 |
| 24 | REACTOME_AMINO_ACIDS_REGULATE_MTORC1 | 0.589 |
| 25 | REACTOME_PHOSPHOLIPASE_C_MEDIATED_CASCADE_FGFR2 | 0.583 |
| 26 | REACTOME_OTHER_INTERLEUKIN_SIGNALING | 0.573 |
| 27 | REACTOME_BILE_ACID_AND_BILE_SALT_METABOLISM | 0.569 |
| 28 | REACTOME_MTOR_SIGNALLING | 0.568 |
| 29 | REACTOME_SIGNALING_BY_FGFR2_IIIA_TM | 0.563 |
| 30 | REACTOME_INTERLEUKIN_7_SIGNALING | 0.562 |
| 31 | REACTOME_DISEASES_OF_IMMUNE_SYSTEM | 0.561 |
| 32 | REACTOME_DOWNSTREAM_SIGNALING_OF_ACTIVATED_FGFR3 | 0.557 |
| 33 | REACTOME_NEGATIVE_REGULATION_OF_FGFR4_SIGNALING | 0.553 |
| 34 | REACTOME_FGFR1_LIGAND_BINDING_AND_ACTIVATION | 0.553 |
| 35 | REACTOME_NEGATIVE_REGULATION_OF_FGFR1_SIGNALING | 0.552 |
| 36 | REACTOME_SIGNALING_BY_ALK_IN_CANCER | 0.548 |
| 37 | REACTOME_RHO_GTPASES_ACTIVATE_NADPH_OXIDASES | 0.541 |
| 38 | REACTOME_PI3K_AKT_ACTIVATION | 0.535 |
| 39 | REACTOME_IRF3_MEDIATED_INDUCTION_OF_TYPE_I_IFN | 0.529 |
| 40 | REACTOME_SIGNALLING_TO_P38_VIA_RIT_AND_RIN | 0.528 |
| 41 | REACTOME_FGFR2B_LIGAND_BINDING_AND_ACTIVATION | 0.527 |
| 42 | REACTOME_SIGNALING_BY_PDGFRA_TRANSMEMBRANE_JUXTAMEMBRANE_AND_KINASE_DOMAIN_MUTANTS | 0.518 |
| 43 | REACTOME_HEME_SIGNALING | 0.512 |
| 44 | REACTOME_CYTOPROTECTION_BY_HMOX1 | 0.502 |
| 45 | REACTOME_FGFR1B_LIGAND_BINDING_AND_ACTIVATION | 0.501 |
| 46 | REACTOME_FLT3_SIGNALING_IN_DISEASE | 0.499 |
| 47 | REACTOME_NEGATIVE_REGULATION_OF_FGFR3_SIGNALING | 0.499 |
| 48 | REACTOME_SYNTHESIS_OF_BILE_ACIDS_AND_BILE_SALTS_VIA_7ALPHA_HYDROXYCHOLESTEROL | 0.492 |
| 49 | REACTOME_MAPK_TARGETS_NUCLEAR_EVENTS_MEDIATED_BY_MAP_KINASES | 0.489 |
| 50 | REACTOME_SEMA4D_INDUCED_CELL_MIGRATION_AND_GROWTH_CONE_COLLAPSE | 0.488 |
| 51 | REACTOME_FGFR1C_LIGAND_BINDING_AND_ACTIVATION | 0.486 |
| 52 | REACTOME_FGFR2_LIGAND_BINDING_AND_ACTIVATION | 0.485 |
| 53 | REACTOME_TRANSCRIPTIONAL_REGULATION_BY_VENTX | 0.485 |
| 54 | REACTOME_GAMMA_CARBOXYLATION_HYPUSINE_FORMATION_AND_ARYLSULFATASE_ACTIVATION | 0.480 |
| 55 | REACTOME_SIGNALING_BY_ERYTHROPOIETIN | 0.479 |
| 56 | REACTOME_FRUCTOSE_METABOLISM | 0.478 |
| 57 | REACTOME_NEGATIVE_FEEDBACK_REGULATION_OF_MAPK_PATHWAY | 0.473 |
| 58 | REACTOME_RAP1_SIGNALLING | 0.473 |
| 59 | REACTOME_REGULATION_OF_KIT_SIGNALING | 0.472 |
| 60 | REACTOME_SYNTHESIS_OF_BILE_ACIDS_AND_BILE_SALTS_VIA_27_HYDROXYCHOLESTEROL | 0.471 |
| 61 | REACTOME_ATORVASTATIN_ADME | 0.467 |
| 62 | REACTOME_SIGNALING_BY_FGFR4_IN_DISEASE | 0.465 |
| 63 | REACTOME_FGFR2C_LIGAND_BINDING_AND_ACTIVATION | 0.462 |
| 64 | REACTOME_ACTIVATION_OF_GENE_EXPRESSION_BY_SREBF_SREBP | 0.456 |
| 65 | REACTOME_INTERLEUKIN_RECEPTOR_SHC_SIGNALING | 0.452 |
| 66 | REACTOME_EGFR_TRANSACTIVATION_BY_GASTRIN | 0.452 |
| 67 | REACTOME_MECP2_REGULATES_TRANSCRIPTION_FACTORS | 0.450 |
| 68 | REACTOME_ACYL_CHAIN_REMODELLING_OF_PC | 0.436 |
| 69 | REACTOME_SHC_MEDIATED_CASCADE_FGFR1 | 0.436 |
| 70 | REACTOME_ENERGY_DEPENDENT_REGULATION_OF_MTOR_BY_LKB1_AMPK | 0.436 |
| 71 | REACTOME_SMOOTH_MUSCLE_CONTRACTION | 0.430 |
| 72 | REACTOME_NOTCH3_ACTIVATION_AND_TRANSMISSION_OF_SIGNAL_TO_THE_NUCLEUS | 0.429 |
| 73 | REACTOME_ACTIVATION_OF_IRF3_IRF7_MEDIATED_BY_TBK1_IKK_EPSILON | 0.425 |
| 74 | REACTOME_TIE2_SIGNALING | 0.421 |
| 75 | REACTOME_SYNTHESIS_OF_BILE_ACIDS_AND_BILE_SALTS | 0.418 |
| 76 | REACTOME_HSF1_DEPENDENT_TRANSACTIVATION | 0.418 |
| 77 | REACTOME_VLDLR_INTERNALISATION_AND_DEGRADATION | 0.414 |
| 78 | REACTOME_ADVANCED_GLYCOSYLATION_ENDPRODUCT_RECEPTOR_SIGNALING | 0.407 |
| 79 | REACTOME_EICOSANOID_LIGAND_BINDING_RECEPTORS | 0.405 |
| 80 | REACTOME_SYNTHESIS_OF_BILE_ACIDS_AND_BILE_SALTS_VIA_24_HYDROXYCHOLESTEROL | 0.403 |
| 81 | REACTOME_CARNITINE_METABOLISM | 0.400 |
| 82 | REACTOME_METABOLISM_OF_FAT_SOLUBLE_VITAMINS | 0.400 |
| 83 | REACTOME_SARS_COV_1_INFECTION | 0.400 |
| 84 | REACTOME_REGULATION_OF_PYRUVATE_DEHYDROGENASE_PDH_COMPLEX | 0.399 |
| 85 | REACTOME_INTERLEUKIN_6_FAMILY_SIGNALING | 0.399 |
| 86 | REACTOME_TRAF6_MEDIATED_NF_KB_ACTIVATION | 0.398 |
| 87 | REACTOME_HUR_ELAVL1_BINDS_AND_STABILIZES_MRNA | 0.397 |
| 88 | REACTOME_TRANSPORT_OF_ORGANIC_ANIONS | 0.395 |
| 89 | REACTOME_TRANSCRIPTIONAL_REGULATION_OF_PLURIPOTENT_STEM_CELLS | 0.394 |
| 90 | REACTOME_GROWTH_HORMONE_RECEPTOR_SIGNALING | 0.393 |
| 91 | REACTOME_ARMS_MEDIATED_ACTIVATION | 0.393 |
| 92 | REACTOME_DSCAM_INTERACTIONS | 0.391 |
| 93 | REACTOME_SIGNALING_BY_MRAS_COMPLEX_MUTANTS | 0.390 |
| 94 | REACTOME_ACTIVATION_OF_PPARGC1A_PGC_1ALPHA_BY_PHOSPHORYLATION | 0.388 |
| 95 | REACTOME_SIGNALING_BY_CYTOSOLIC_FGFR1_FUSION_MUTANTS | 0.387 |
| 96 | REACTOME_PYROPTOSIS | 0.386 |
| 97 | REACTOME_PLATELET_SENSITIZATION_BY_LDL | 0.382 |
| 98 | REACTOME_SPRY_REGULATION_OF_FGF_SIGNALING | 0.380 |
| 99 | REACTOME_FRS_MEDIATED_FGFR2_SIGNALING | 0.380 |
| 100 | REACTOME_INTERLEUKIN_37_SIGNALING | 0.379 |
| 101 | REACTOME_CD209_DC_SIGN_SIGNALING | 0.378 |
| 102 | REACTOME_MAPK3_ERK1_ACTIVATION | 0.367 |
| 103 | REACTOME_EPHB_MEDIATED_FORWARD_SIGNALING | 0.365 |
| 104 | REACTOME_TRANSPORT_OF_VITAMINS_NUCLEOSIDES_AND_RELATED_MOLECULES | 0.364 |
| 105 | REACTOME_FRS_MEDIATED_FGFR4_SIGNALING | 0.364 |
| 106 | REACTOME_RECYCLING_OF_BILE_ACIDS_AND_SALTS | 0.363 |
| 107 | REACTOME_REGULATION_OF_IFNA_IFNB_SIGNALING | 0.361 |
| 108 | REACTOME_TAK1_DEPENDENT_IKK_AND_NF_KAPPA_B_ACTIVATION | 0.357 |
| 109 | REACTOME_HEME_DEGRADATION | 0.356 |
| 110 | REACTOME_TRP_CHANNELS | 0.353 |
| 111 | REACTOME_DOWNSTREAM_SIGNALING_OF_ACTIVATED_FGFR4 | 0.352 |
| 112 | REACTOME_RORA_ACTIVATES_GENE_EXPRESSION | 0.347 |
| 113 | REACTOME_NRIF_SIGNALS_CELL_DEATH_FROM_THE_NUCLEUS | 0.345 |
| 114 | REACTOME_REMOVAL_OF_AMINOTERMINAL_PROPEPTIDES_FROM_GAMMA_CARBOXYLATED_PROTEINS | 0.345 |
| 115 | REACTOME_FRS_MEDIATED_FGFR1_SIGNALING | 0.344 |
| 116 | REACTOME_SIGNALING_BY_FLT3_ITD_AND_TKD_MUTANTS | 0.343 |
| 117 | REACTOME_REGULATION_OF_CHOLESTEROL_BIOSYNTHESIS_BY_SREBP_SREBF | 0.341 |
| 118 | REACTOME_INTERLEUKIN_1_PROCESSING | 0.341 |
| 119 | REACTOME_FLT3_SIGNALING_BY_CBL_MUTANTS | 0.338 |
| 120 | REACTOME_METABOLISM_OF_STEROID_HORMONES | 0.338 |
| 121 | REACTOME_REGULATED_PROTEOLYSIS_OF_P75NTR | 0.336 |
| 122 | REACTOME_NR1H2_NR1H3_REGULATE_GENE_EXPRESSION_LINKED_TO_LIPOGENESIS | 0.335 |
| 123 | REACTOME_EGFR_INTERACTS_WITH_PHOSPHOLIPASE_C_GAMMA | 0.334 |
| 124 | REACTOME_IRF3_MEDIATED_ACTIVATION_OF_TYPE_1_IFN | 0.334 |
| 125 | REACTOME_MAPK1_ERK2_ACTIVATION | 0.333 |
| 126 | REACTOME_APOPTOTIC_EXECUTION_PHASE | 0.332 |
| 127 | REACTOME_METABOLISM_OF_PORPHYRINS | 0.330 |
| 128 | REACTOME_BH3_ONLY_PROTEINS_ASSOCIATE_WITH_AND_INACTIVATE_ANTI_APOPTOTIC_BCL_2_MEMBERS | 0.329 |
| 129 | REACTOME_PROSTANOID_LIGAND_RECEPTORS | 0.327 |
| 130 | REACTOME_RUNX3_REGULATES_IMMUNE_RESPONSE_AND_CELL_MIGRATION | 0.324 |
| 131 | REACTOME_SIGNALING_BY_MEMBRANE_TETHERED_FUSIONS_OF_PDGFRA_OR_PDGFRB | 0.322 |
| 132 | REACTOME_CHOLESTEROL_BIOSYNTHESIS | 0.321 |
| 133 | REACTOME_ADP_SIGNALLING_THROUGH_P2Y_PURINOCEPTOR_1 | 0.320 |
| 134 | REACTOME_ERK_MAPK_TARGETS | 0.318 |
| 135 | REACTOME_SIGNAL_ATTENUATION | 0.317 |
| 136 | REACTOME_NF_KB_IS_ACTIVATED_AND_SIGNALS_SURVIVAL | 0.316 |
| 137 | REACTOME_PI_3K_CASCADE_FGFR3 | 0.316 |
| 138 | REACTOME_ABACAVIR_TRANSMEMBRANE_TRANSPORT | 0.316 |
| 139 | REACTOME_SYNTHESIS_OF_PA | 0.315 |
| 140 | REACTOME_GLUCOCORTICOID_BIOSYNTHESIS | 0.310 |
| 141 | REACTOME_NETRIN_1_SIGNALING | 0.310 |
| 142 | REACTOME_REGULATION_OF_IFNG_SIGNALING | 0.308 |
| 143 | REACTOME_EARLY_PHASE_OF_HIV_LIFE_CYCLE | 0.300 |
| 144 | REACTOME_G_PROTEIN_BETA_GAMMA_SIGNALLING | 0.299 |
| 145 | REACTOME_ACYL_CHAIN_REMODELLING_OF_PS | 0.297 |
| 146 | REACTOME_TRANSCRIPTION_OF_E2F_TARGETS_UNDER_NEGATIVE_CONTROL_BY_DREAM_COMPLEX | 0.296 |
| 147 | REACTOME_P38MAPK_EVENTS | 0.293 |
| 148 | REACTOME_HYDROLYSIS_OF_LPC | 0.293 |
| 149 | REACTOME_NR1H2_NR1H3_REGULATE_GENE_EXPRESSION_TO_CONTROL_BILE_ACID_HOMEOSTASIS | 0.292 |
| 150 | REACTOME_SEMA4D_IN_SEMAPHORIN_SIGNALING | 0.292 |
| 151 | REACTOME_ACYL_CHAIN_REMODELLING_OF_PG | 0.290 |
| 152 | REACTOME_TGF_BETA_RECEPTOR_SIGNALING_ACTIVATES_SMADS | 0.288 |
| 153 | REACTOME_FGFR2_ALTERNATIVE_SPLICING | 0.285 |
| 154 | REACTOME_CYTOCHROME_C_MEDIATED_APOPTOTIC_RESPONSE | 0.284 |
| 155 | REACTOME_FATTY_ACIDS | 0.282 |
| 156 | REACTOME_ACTIVATED_TAK1_MEDIATES_P38_MAPK_ACTIVATION | 0.282 |
| 157 | REACTOME_VITAMIN_D_CALCIFEROL_METABOLISM | 0.282 |
| 158 | REACTOME_PTK6_PROMOTES_HIF1A_STABILIZATION | 0.281 |
| 159 | REACTOME_CARGO_TRAFFICKING_TO_THE_PERICILIARY_MEMBRANE | 0.281 |
| 160 | REACTOME_SYNTHESIS_SECRETION_AND_INACTIVATION_OF_GLUCOSE_DEPENDENT_INSULINOTROPIC_POLYPEPTIDE_GIP | 0.280 |
| 161 | REACTOME_ZBP1_DAI_MEDIATED_INDUCTION_OF_TYPE_I_IFNS | 0.278 |
| 162 | REACTOME_POU5F1_OCT4_SOX2_NANOG_ACTIVATE_GENES_RELATED_TO_PROLIFERATION | 0.278 |
| 163 | REACTOME_BBSOME_MEDIATED_CARGO_TARGETING_TO_CILIUM | 0.276 |
| 164 | REACTOME_COMMON_PATHWAY_OF_FIBRIN_CLOT_FORMATION | 0.274 |
| 165 | REACTOME_SIGNAL_AMPLIFICATION | 0.272 |
| 166 | REACTOME_RUNX1_REGULATES_TRANSCRIPTION_OF_GENES_INVOLVED_IN_WNT_SIGNALING | 0.271 |
| 167 | REACTOME_ERBB2_ACTIVATES_PTK6_SIGNALING | 0.270 |
| 168 | REACTOME_VEGF_LIGAND_RECEPTOR_INTERACTIONS | 0.269 |
| 169 | REACTOME_RHOBTB1_GTPASE_CYCLE | 0.269 |
| 170 | REACTOME_RETROGRADE_NEUROTROPHIN_SIGNALLING | 0.268 |
| 171 | REACTOME_SYNDECAN_INTERACTIONS | 0.267 |
| 172 | REACTOME_SHC1_EVENTS_IN_EGFR_SIGNALING | 0.266 |
| 173 | REACTOME_PTK6_EXPRESSION | 0.265 |
| 174 | REACTOME_INTERLEUKIN_12_SIGNALING | 0.264 |
| 175 | REACTOME_ACYL_CHAIN_REMODELLING_OF_PI | 0.263 |
| 176 | REACTOME_RESPONSE_OF_MTB_TO_PHAGOCYTOSIS | 0.262 |
| 177 | REACTOME_ACTIVATION_OF_BH3_ONLY_PROTEINS | 0.260 |
| 178 | REACTOME_RAF_INDEPENDENT_MAPK1_3_ACTIVATION | 0.258 |
| 179 | REACTOME_ERYTHROPOIETIN_ACTIVATES_PHOSPHOINOSITIDE_3_KINASE_PI3K | 0.255 |
| 180 | REACTOME_NR1H2_NR1H3_REGULATE_GENE_EXPRESSION_TO_LIMIT_CHOLESTEROL_UPTAKE | 0.250 |
| 181 | REACTOME_P75NTR_REGULATES_AXONOGENESIS | 0.250 |
| 182 | REACTOME_WNT5A_DEPENDENT_INTERNALIZATION_OF_FZD4 | 0.249 |
| 183 | REACTOME_GRB2_EVENTS_IN_ERBB2_SIGNALING | 0.248 |
| 184 | REACTOME_INTERLEUKIN_6_SIGNALING | 0.248 |
| 185 | REACTOME_CHEMOKINE_RECEPTORS_BIND_CHEMOKINES | 0.248 |
| 186 | REACTOME_RHO_GTPASES_ACTIVATE_WASPS_AND_WAVES | 0.247 |
| 187 | REACTOME_NR1H2_AND_NR1H3_MEDIATED_SIGNALING | 0.246 |
| 188 | REACTOME_ACTIVATION_OF_BAD_AND_TRANSLOCATION_TO_MITOCHONDRIA | 0.246 |
| 189 | REACTOME_TGF_BETA_RECEPTOR_SIGNALING_IN_EMT_EPITHELIAL_TO_MESENCHYMAL_TRANSITION | 0.242 |
| 190 | REACTOME_SIGNALING_BY_RETINOIC_ACID | 0.239 |
| 191 | REACTOME_ACTIVATION_OF_TRKA_RECEPTORS | 0.238 |
| 192 | REACTOME_HDR_THROUGH_SINGLE_STRAND_ANNEALING_SSA | 0.238 |
| 193 | REACTOME_INWARDLY_RECTIFYING_K_CHANNELS | 0.238 |
| 194 | REACTOME_TICAM1_DEPENDENT_ACTIVATION_OF_IRF3_IRF7 | 0.238 |
| 195 | REACTOME_IRAK4_DEFICIENCY_TLR2_4 | 0.237 |
| 196 | REACTOME_TNFR1_INDUCED_PROAPOPTOTIC_SIGNALING | 0.236 |
| 197 | REACTOME_TRANSCRIPTIONAL_ACTIVITY_OF_SMAD2_SMAD3_SMAD4_HETEROTRIMER | 0.235 |
| 198 | REACTOME_SIGNALING_BY_TGF_BETA_RECEPTOR_COMPLEX_IN_CANCER | 0.235 |
| 199 | REACTOME_RHO_GTPASES_ACTIVATE_ROCKS | 0.234 |
| 200 | REACTOME_ORGANIC_ANION_TRANSPORT | 0.232 |
| 201 | REACTOME_REGULATED_NECROSIS | 0.232 |
| 202 | REACTOME_G_BETA_GAMMA_SIGNALLING_THROUGH_PI3KGAMMA | 0.230 |
| 203 | REACTOME_SIGNALLING_TO_RAS | 0.230 |
| 204 | REACTOME_SENSORY_PERCEPTION_OF_TASTE | 0.230 |
| 205 | REACTOME_DISASSEMBLY_OF_THE_DESTRUCTION_COMPLEX_AND_RECRUITMENT_OF_AXIN_TO_THE_MEMBRANE | 0.229 |
| 206 | REACTOME_ACTIVATION_OF_SMO | 0.229 |
| 207 | REACTOME_ACYL_CHAIN_REMODELLING_OF_PE | 0.228 |
| 208 | REACTOME_RIP_MEDIATED_NFKB_ACTIVATION_VIA_ZBP1 | 0.227 |
| 209 | REACTOME_IKBA_VARIANT_LEADS_TO_EDA_ID | 0.226 |
| 210 | REACTOME_SHC_MEDIATED_CASCADE_FGFR4 | 0.225 |
| 211 | REACTOME_ACTIVATED_NTRK3_SIGNALS_THROUGH_PI3K | 0.224 |
| 212 | REACTOME_OREXIN_AND_NEUROPEPTIDES_FF_AND_QRFP_BIND_TO_THEIR_RESPECTIVE_RECEPTORS | 0.220 |
| 213 | REACTOME_SMAC_XIAP_REGULATED_APOPTOTIC_RESPONSE | 0.219 |
| 214 | REACTOME_KILLING_MECHANISMS | 0.219 |
| 215 | REACTOME_NUCLEOTIDE_BIOSYNTHESIS | 0.219 |
| 216 | REACTOME_PYRUVATE_METABOLISM | 0.219 |
| 217 | REACTOME_GP1B_IX_V_ACTIVATION_SIGNALLING | 0.218 |
| 218 | REACTOME_REGULATION_OF_TNFR1_SIGNALING | 0.217 |
| 219 | REACTOME_EPHA_MEDIATED_GROWTH_CONE_COLLAPSE | 0.216 |
| 220 | REACTOME_GABA_RECEPTOR_ACTIVATION | 0.216 |
| 221 | REACTOME_PROSTACYCLIN_SIGNALLING_THROUGH_PROSTACYCLIN_RECEPTOR | 0.215 |
| 222 | REACTOME_SYNTHESIS_OF_PIPS_AT_THE_PLASMA_MEMBRANE | 0.215 |
| 223 | REACTOME_MELANIN_BIOSYNTHESIS | 0.215 |
| 224 | REACTOME_TRANSCRIPTIONAL_REGULATION_BY_E2F6 | 0.212 |
| 225 | REACTOME_VITAMIN_B5_PANTOTHENATE_METABOLISM | 0.212 |
| 226 | REACTOME_DEFECTIVE_FACTOR_VIII_CAUSES_HEMOPHILIA_A | 0.211 |
| 227 | REACTOME_SIGNALING_BY_NTRK3_TRKC | 0.210 |
| 228 | REACTOME_APOPTOTIC_FACTOR_MEDIATED_RESPONSE | 0.209 |
| 229 | REACTOME_NR1H2_NR1H3_REGULATE_GENE_EXPRESSION_LINKED_TO_GLUCONEOGENESIS | 0.209 |
| 230 | REACTOME_RHOU_GTPASE_CYCLE | 0.208 |
| 231 | REACTOME_NEGATIVE_REGULATION_OF_FLT3 | 0.207 |
| 232 | REACTOME_HS_GAG_DEGRADATION | 0.207 |
| 233 | REACTOME_STAT5_ACTIVATION | 0.204 |
| 234 | REACTOME_APOPTOTIC_CLEAVAGE_OF_CELLULAR_PROTEINS | 0.203 |
| 235 | REACTOME_MAP2K_AND_MAPK_ACTIVATION | 0.203 |
| 236 | REACTOME_NUCLEAR_EVENTS_KINASE_AND_TRANSCRIPTION_FACTOR_ACTIVATION | 0.200 |
| 237 | REACTOME_REGULATION_OF_INNATE_IMMUNE_RESPONSES_TO_CYTOSOLIC_DNA | 0.199 |
| 238 | REACTOME_IKK_COMPLEX_RECRUITMENT_MEDIATED_BY_RIP1 | 0.198 |
| 239 | REACTOME_DEGRADATION_OF_AXIN | 0.198 |
| 240 | REACTOME_FATTY_ACYL_COA_BIOSYNTHESIS | 0.197 |
| 241 | REACTOME_DEFECTIVE_FACTOR_IX_CAUSES_HEMOPHILIA_B | 0.196 |
| 242 | REACTOME_RA_BIOSYNTHESIS_PATHWAY | 0.196 |
| 243 | REACTOME_PYRUVATE_METABOLISM_AND_CITRIC_ACID_TCA_CYCLE | 0.195 |
| 244 | REACTOME_DAP12_SIGNALING | 0.194 |
| 245 | REACTOME_FLT3_SIGNALING_THROUGH_SRC_FAMILY_KINASES | 0.194 |
| 246 | REACTOME_NGF_INDEPENDANT_TRKA_ACTIVATION | 0.193 |
| 247 | REACTOME_INTERLEUKIN_20_FAMILY_SIGNALING | 0.193 |
| 248 | REACTOME_ELECTRIC_TRANSMISSION_ACROSS_GAP_JUNCTIONS | 0.192 |
| 249 | REACTOME_EGFR_DOWNREGULATION | 0.192 |
| 250 | REACTOME_PI3K_EVENTS_IN_ERBB4_SIGNALING | 0.191 |
| 251 | REACTOME_THROMBIN_SIGNALLING_THROUGH_PROTEINASE_ACTIVATED_RECEPTORS_PARS | 0.190 |
| 252 | REACTOME_DEACTIVATION_OF_THE_BETA_CATENIN_TRANSACTIVATING_COMPLEX | 0.189 |
| 253 | REACTOME_RUNX1_REGULATES_TRANSCRIPTION_OF_GENES_INVOLVED_IN_BCR_SIGNALING | 0.188 |
| 254 | REACTOME_APOPTOTIC_CLEAVAGE_OF_CELL_ADHESION_PROTEINS | 0.187 |
| 255 | REACTOME_PD_1_SIGNALING | 0.185 |
| 256 | REACTOME_RUNX1_REGULATES_ESTROGEN_RECEPTOR_MEDIATED_TRANSCRIPTION | 0.183 |
| 257 | REACTOME_CLASS_C_3_METABOTROPIC_GLUTAMATE_PHEROMONE_RECEPTORS | 0.183 |
| 258 | REACTOME_P75NTR_SIGNALS_VIA_NF_KB | 0.182 |
| 259 | REACTOME_RHOF_GTPASE_CYCLE | 0.180 |
| 260 | REACTOME_CASPASE_ACTIVATION_VIA_DEPENDENCE_RECEPTORS_IN_THE_ABSENCE_OF_LIGAND | 0.179 |
| 261 | REACTOME_NEGATIVE_REGULATION_OF_MET_ACTIVITY | 0.179 |
| 262 | REACTOME_TP53_REGULATES_TRANSCRIPTION_OF_DNA_REPAIR_GENES | 0.178 |
| 263 | REACTOME_MAP3K8_TPL2_DEPENDENT_MAPK1_3_ACTIVATION | 0.177 |
| 264 | REACTOME_INTERACTIONS_OF_VPR_WITH_HOST_CELLULAR_PROTEINS | 0.176 |
| 265 | REACTOME_DAP12_INTERACTIONS | 0.176 |
| 266 | REACTOME_SIGNALING_BY_LEPTIN | 0.174 |
| 267 | REACTOME_CASPASE_ACTIVATION_VIA_EXTRINSIC_APOPTOTIC_SIGNALLING_PATHWAY | 0.174 |
| 268 | REACTOME_HEPARAN_SULFATE_HEPARIN_HS_GAG_METABOLISM | 0.172 |
| 269 | REACTOME_ERKS_ARE_INACTIVATED | 0.172 |
| 270 | REACTOME_TNF_RECEPTOR_SUPERFAMILY_TNFSF_MEMBERS_MEDIATING_NON_CANONICAL_NF_KB_PATHWAY | 0.171 |
| 271 | REACTOME_TRAF6_MEDIATED_IRF7_ACTIVATION | 0.170 |
| 272 | REACTOME_STAT5_ACTIVATION_DOWNSTREAM_OF_FLT3_ITD_MUTANTS | 0.170 |
| 273 | REACTOME_FORMATION_OF_APOPTOSOME | 0.170 |
| 274 | REACTOME_CASPASE_ACTIVATION_VIA_DEATH_RECEPTORS_IN_THE_PRESENCE_OF_LIGAND | 0.169 |
| 275 | REACTOME_GAP_JUNCTION_ASSEMBLY | 0.168 |
| 276 | REACTOME_VOLTAGE_GATED_POTASSIUM_CHANNELS | 0.168 |
| 277 | REACTOME_TRAF3_DEPENDENT_IRF_ACTIVATION_PATHWAY | 0.167 |
| 278 | REACTOME_CLEC7A_INFLAMMASOME_PATHWAY | 0.166 |
| 279 | REACTOME_FORMYL_PEPTIDE_RECEPTORS_BIND_FORMYL_PEPTIDES_AND_MANY_OTHER_LIGANDS | 0.166 |
| 280 | REACTOME_PARACETAMOL_ADME | 0.165 |
| 281 | REACTOME_GENERATION_OF_SECOND_MESSENGER_MOLECULES | 0.165 |
| 282 | REACTOME_PREGNENOLONE_BIOSYNTHESIS | 0.165 |
| 283 | REACTOME_RHOV_GTPASE_CYCLE | 0.165 |
| 284 | REACTOME_ENDOGENOUS_STEROLS | 0.165 |
| 285 | REACTOME_EGR2_AND_SOX10_MEDIATED_INITIATION_OF_SCHWANN_CELL_MYELINATION | 0.164 |
| 286 | REACTOME_ARYL_HYDROCARBON_RECEPTOR_SIGNALLING | 0.164 |
| 287 | REACTOME_REGULATION_OF_TLR_BY_ENDOGENOUS_LIGAND | 0.163 |
| 288 | REACTOME_NUCLEOTIDE_LIKE_PURINERGIC_RECEPTORS | 0.163 |
| 289 | REACTOME_FANCONI_ANEMIA_PATHWAY | 0.161 |
| 290 | REACTOME_ACTIVATION_OF_NIMA_KINASES_NEK9_NEK6_NEK7 | 0.161 |
| 291 | REACTOME_PHOSPHOLIPASE_C_MEDIATED_CASCADE_FGFR4 | 0.160 |
| 292 | REACTOME_NUCLEAR_EVENTS_STIMULATED_BY_ALK_SIGNALING_IN_CANCER | 0.158 |
| 293 | REACTOME_STAT3_NUCLEAR_EVENTS_DOWNSTREAM_OF_ALK_SIGNALING | 0.158 |
| 294 | REACTOME_DEFECTS_OF_CONTACT_ACTIVATION_SYSTEM_CAS_AND_KALLIKREIN_KININ_SYSTEM_KKS | 0.157 |
| 295 | REACTOME_TICAM1_RIP1_MEDIATED_IKK_COMPLEX_RECRUITMENT | 0.157 |
| 296 | REACTOME_RND1_GTPASE_CYCLE | 0.156 |
| 297 | REACTOME_ABACAVIR_ADME | 0.156 |
| 298 | REACTOME_NADE_MODULATES_DEATH_SIGNALLING | 0.156 |
| 299 | REACTOME_CGMP_EFFECTS | 0.155 |
| 300 | REACTOME_GABA_B_RECEPTOR_ACTIVATION | 0.155 |
| 301 | REACTOME_NR1H3_NR1H2_REGULATE_GENE_EXPRESSION_LINKED_TO_CHOLESTEROL_TRANSPORT_AND_EFFLUX | 0.154 |
| 302 | REACTOME_FREE_FATTY_ACIDS_REGULATE_INSULIN_SECRETION | 0.152 |
| 303 | REACTOME_MITOCHONDRIAL_PROTEIN_IMPORT | 0.151 |
| 304 | REACTOME_INCRETIN_SYNTHESIS_SECRETION_AND_INACTIVATION | 0.150 |
| 305 | REACTOME_PLASMA_LIPOPROTEIN_CLEARANCE | 0.150 |
| 306 | REACTOME_CHAPERONE_MEDIATED_AUTOPHAGY | 0.149 |
| 307 | REACTOME_AKT_PHOSPHORYLATES_TARGETS_IN_THE_CYTOSOL | 0.147 |
| 308 | REACTOME_INFLAMMASOMES | 0.147 |
| 309 | REACTOME_SYNTHESIS_OF_KETONE_BODIES | 0.147 |
| 310 | REACTOME_SUMOYLATION_OF_TRANSCRIPTION_COFACTORS | 0.147 |
| 311 | REACTOME_GAMMA_CARBOXYLATION_TRANSPORT_AND_AMINO_TERMINAL_CLEAVAGE_OF_PROTEINS | 0.147 |
| 312 | REACTOME_GOLGI_CISTERNAE_PERICENTRIOLAR_STACK_REORGANIZATION | 0.146 |
| 313 | REACTOME_LOSS_OF_FUNCTION_OF_SMAD2_3_IN_CANCER | 0.146 |
| 314 | REACTOME_SIGNALING_BY_CTNNB1_PHOSPHO_SITE_MUTANTS | 0.145 |
| 315 | REACTOME_EFFECTS_OF_PIP2_HYDROLYSIS | 0.145 |
| 316 | REACTOME_INTERLEUKIN_35_SIGNALLING | 0.144 |
| 317 | REACTOME_PHASE_3_RAPID_REPOLARISATION | 0.144 |
| 318 | REACTOME_THE_PHOTOTRANSDUCTION_CASCADE | 0.144 |
| 319 | REACTOME_CITRIC_ACID_CYCLE_TCA_CYCLE | 0.144 |
| 320 | REACTOME_P75NTR_RECRUITS_SIGNALLING_COMPLEXES | 0.144 |
| 321 | REACTOME_EXPORT_OF_VIRAL_RIBONUCLEOPROTEINS_FROM_NUCLEUS | 0.143 |
| 322 | REACTOME_TRIF_MEDIATED_PROGRAMMED_CELL_DEATH | 0.143 |
| 323 | REACTOME_RHOQ_GTPASE_CYCLE | 0.143 |
| 324 | REACTOME_INTRINSIC_PATHWAY_OF_FIBRIN_CLOT_FORMATION | 0.142 |
| 325 | REACTOME_NITRIC_OXIDE_STIMULATES_GUANYLATE_CYCLASE | 0.141 |
| 326 | REACTOME_NR1H2_NR1H3_REGULATE_GENE_EXPRESSION_LINKED_TO_TRIGLYCERIDE_LIPOLYSIS_IN_ADIPOSE | 0.140 |
| 327 | REACTOME_ONCOGENE_INDUCED_SENESCENCE | 0.140 |
| 328 | REACTOME_SIGNALING_BY_CSF3_G_CSF | 0.139 |
| 329 | REACTOME_TP53_REGULATES_TRANSCRIPTION_OF_CELL_DEATH_GENES | 0.139 |
| 330 | REACTOME_REGULATION_OF_TP53_ACTIVITY_THROUGH_METHYLATION | 0.139 |
| 331 | REACTOME_CONSTITUTIVE_SIGNALING_BY_OVEREXPRESSED_ERBB2 | 0.139 |
| 332 | REACTOME_POLO_LIKE_KINASE_MEDIATED_EVENTS | 0.138 |
| 333 | REACTOME_SUPPRESSION_OF_PHAGOSOMAL_MATURATION | 0.138 |
| 334 | REACTOME_AZATHIOPRINE_ADME | 0.138 |
| 335 | REACTOME_SIGNAL_REGULATORY_PROTEIN_FAMILY_INTERACTIONS | 0.138 |
| 336 | REACTOME_INTERACTION_BETWEEN_L1_AND_ANKYRINS | 0.138 |
| 337 | REACTOME_TNFS_BIND_THEIR_PHYSIOLOGICAL_RECEPTORS | 0.137 |
| 338 | REACTOME_SIGNALING_BY_WNT_IN_CANCER | 0.137 |
| 339 | REACTOME_E3_UBIQUITIN_LIGASES_UBIQUITINATE_TARGET_PROTEINS | 0.137 |
| 340 | REACTOME_STABILIZATION_OF_P53 | 0.137 |
| 341 | REACTOME_INTEGRIN_SIGNALING | 0.137 |
| 342 | REACTOME_ACYL_CHAIN_REMODELING_OF_CL | 0.136 |
| 343 | REACTOME_CHREBP_ACTIVATES_METABOLIC_GENE_EXPRESSION | 0.135 |
| 344 | REACTOME_HORMONE_LIGAND_BINDING_RECEPTORS | 0.134 |
| 345 | REACTOME_NICOTINATE_METABOLISM | 0.134 |
| 346 | REACTOME_TRANSCRIPTIONAL_ACTIVATION_OF_MITOCHONDRIAL_BIOGENESIS | 0.134 |
| 347 | REACTOME_SYNTHESIS_SECRETION_AND_DEACYLATION_OF_GHRELIN | 0.134 |
| 348 | REACTOME_SMAD2_SMAD3_SMAD4_HETEROTRIMER_REGULATES_TRANSCRIPTION | 0.133 |
| 349 | REACTOME_TRAF6_MEDIATED_INDUCTION_OF_TAK1_COMPLEX_WITHIN_TLR4_COMPLEX | 0.132 |
| 350 | REACTOME_RUNX2_REGULATES_CHONDROCYTE_MATURATION | 0.132 |
| 351 | REACTOME_MYOGENESIS | 0.132 |
| 352 | REACTOME_CD163_MEDIATING_AN_ANTI_INFLAMMATORY_RESPONSE | 0.132 |
| 353 | REACTOME_TNFR1_MEDIATED_CERAMIDE_PRODUCTION | 0.131 |
| 354 | REACTOME_INTERLEUKIN_12_FAMILY_SIGNALING | 0.131 |
| 355 | REACTOME_SHC_MEDIATED_CASCADE_FGFR3 | 0.130 |
| 356 | REACTOME_RHOBTB_GTPASE_CYCLE | 0.130 |
| 357 | REACTOME_NOTCH4_INTRACELLULAR_DOMAIN_REGULATES_TRANSCRIPTION | 0.130 |
| 358 | REACTOME_IRAK1_RECRUITS_IKK_COMPLEX | 0.129 |
| 359 | REACTOME_RUNX1_REGULATES_TRANSCRIPTION_OF_GENES_INVOLVED_IN_DIFFERENTIATION_OF_KERATINOCYTES | 0.128 |
| 360 | REACTOME_NF_KB_ACTIVATION_THROUGH_FADD_RIP_1_PATHWAY_MEDIATED_BY_CASPASE_8_AND_10 | 0.128 |
| 361 | REACTOME_SIGNALING_BY_NODAL | 0.128 |
| 362 | REACTOME_PLASMA_LIPOPROTEIN_ASSEMBLY | 0.128 |
| 363 | REACTOME_NS1_MEDIATED_EFFECTS_ON_HOST_PATHWAYS | 0.128 |
| 364 | REACTOME_SIGNALING_BY_HIPPO | 0.127 |
| 365 | REACTOME_SYNTHESIS_OF_LEUKOTRIENES_LT_AND_EOXINS_EX | 0.127 |
| 366 | REACTOME_REGULATION_OF_NF_KAPPA_B_SIGNALING | 0.127 |
| 367 | REACTOME_RHOJ_GTPASE_CYCLE | 0.127 |
| 368 | REACTOME_FRS_MEDIATED_FGFR3_SIGNALING | 0.127 |
| 369 | REACTOME_CONDENSATION_OF_PROMETAPHASE_CHROMOSOMES | 0.126 |
| 370 | REACTOME_SIGNALING_BY_ACTIVIN | 0.126 |
| 371 | REACTOME_ESTROGEN_STIMULATED_SIGNALING_THROUGH_PRKCZ | 0.126 |
| 372 | REACTOME_THE_ROLE_OF_NEF_IN_HIV_1_REPLICATION_AND_DISEASE_PATHOGENESIS | 0.125 |
| 373 | REACTOME_TRAFFICKING_OF_AMPA_RECEPTORS | 0.124 |
| 374 | REACTOME_APOPTOSIS_INDUCED_DNA_FRAGMENTATION | 0.124 |
| 375 | REACTOME_SIGNALING_BY_FLT3_FUSION_PROTEINS | 0.124 |
| 376 | REACTOME_AUF1_HNRNP_D0_BINDS_AND_DESTABILIZES_MRNA | 0.124 |
| 377 | REACTOME_NICOTINAMIDE_SALVAGING | 0.124 |
| 378 | REACTOME_ERYTHROPOIETIN_ACTIVATES_STAT5 | 0.124 |
| 379 | REACTOME_METHYLATION | 0.124 |
| 380 | REACTOME_DEGRADATION_OF_DVL | 0.123 |
| 381 | REACTOME_INTERLEUKIN_23_SIGNALING | 0.123 |
| 382 | REACTOME_GPER1_SIGNALING | 0.123 |
| 383 | REACTOME_MISCELLANEOUS_SUBSTRATES | 0.123 |
| 384 | REACTOME_NOTCH1_INTRACELLULAR_DOMAIN_REGULATES_TRANSCRIPTION | 0.123 |
| 385 | REACTOME_EXTRINSIC_PATHWAY_OF_FIBRIN_CLOT_FORMATION | 0.122 |
| 386 | REACTOME_TP53_REGULATES_TRANSCRIPTION_OF_CELL_CYCLE_GENES | 0.121 |
| 387 | REACTOME_CA2_PATHWAY | 0.121 |
| 388 | REACTOME_FORMATION_OF_FIBRIN_CLOT_CLOTTING_CASCADE | 0.121 |
| 389 | REACTOME_GLUCAGON_LIKE_PEPTIDE_1_GLP1_REGULATES_INSULIN_SECRETION | 0.119 |
| 390 | REACTOME_TRNA_MODIFICATION_IN_THE_NUCLEUS_AND_CYTOSOL | 0.119 |
| 391 | REACTOME_INTERLEUKIN_9_SIGNALING | 0.119 |
| 392 | REACTOME_TP53_REGULATES_TRANSCRIPTION_OF_GENES_INVOLVED_IN_G2_CELL_CYCLE_ARREST | 0.118 |
| 393 | REACTOME_COLLAGEN_DEGRADATION | 0.118 |
| 394 | REACTOME_INFECTION_WITH_MYCOBACTERIUM_TUBERCULOSIS | 0.117 |
| 395 | REACTOME_STRIATED_MUSCLE_CONTRACTION | 0.117 |
| 396 | REACTOME_PURINERGIC_SIGNALING_IN_LEISHMANIASIS_INFECTION | 0.117 |
| 397 | REACTOME_NEUREXINS_AND_NEUROLIGINS | 0.117 |
| 398 | REACTOME_REGULATION_OF_HMOX1_EXPRESSION_AND_ACTIVITY | 0.116 |
| 399 | REACTOME_PURINE_RIBONUCLEOSIDE_MONOPHOSPHATE_BIOSYNTHESIS | 0.116 |
| 400 | REACTOME_SYNTHESIS_OF_PC | 0.116 |
| 401 | REACTOME_HISTIDINE_CATABOLISM | 0.115 |
| 402 | REACTOME_DEPOLYMERISATION_OF_THE_NUCLEAR_LAMINA | 0.115 |
| 403 | REACTOME_TRIGLYCERIDE_METABOLISM | 0.114 |
| 404 | REACTOME_HOMOLOGOUS_DNA_PAIRING_AND_STRAND_EXCHANGE | 0.114 |
| 405 | REACTOME_ACTIVATION_OF_CASPASES_THROUGH_APOPTOSOME_MEDIATED_CLEAVAGE | 0.113 |
| 406 | REACTOME_ACTIVATION_OF_MATRIX_METALLOPROTEINASES | 0.113 |
| 407 | REACTOME_ATTACHMENT_AND_ENTRY | 0.112 |
| 408 | REACTOME_DISEASES_ASSOCIATED_WITH_SURFACTANT_METABOLISM | 0.111 |
| 409 | REACTOME_ORGANIC_CATION_ANION_ZWITTERION_TRANSPORT | 0.111 |
| 410 | REACTOME_DISEASES_OF_DNA_REPAIR | 0.110 |
| 411 | REACTOME_CHYLOMICRON_ASSEMBLY | 0.110 |
| 412 | REACTOME_SIGNALING_BY_MAPK_MUTANTS | 0.110 |
| 413 | REACTOME_SYNTHESIS_OF_DOLICHYL_PHOSPHATE | 0.109 |
| 414 | REACTOME_NEF_MEDIATES_DOWN_MODULATION_OF_CELL_SURFACE_RECEPTORS_BY_RECRUITING_THEM_TO_CLATHRIN_ADAPTERS | 0.109 |
| 415 | REACTOME_TRANSLATION_OF_SARSCOV1_STRUCTURAL_PROTEINS | 0.109 |
| 416 | REACTOME_NOTCH4_ACTIVATION_AND_TRANSMISSION_OF_SIGNAL_TO_THE_NUCLEUS | 0.109 |
| 417 | REACTOME_SIGNALING_BY_NOTCH3 | 0.109 |
| 418 | REACTOME_EICOSANOIDS | 0.109 |
| 419 | REACTOME_ACTIVATED_NTRK2_SIGNALS_THROUGH_PI3K | 0.108 |
| 420 | REACTOME_FGFRL1_MODULATION_OF_FGFR1_SIGNALING | 0.108 |
| 421 | REACTOME_RETINOID_CYCLE_DISEASE_EVENTS | 0.108 |
| 422 | REACTOME_PYRIMIDINE_CATABOLISM | 0.108 |
| 423 | REACTOME_SYNTHESIS_OF_SUBSTRATES_IN_N_GLYCAN_BIOSYTHESIS | 0.107 |
| 424 | REACTOME_DEFENSINS | 0.107 |
| 425 | REACTOME_REGULATION_OF_TP53_ACTIVITY_THROUGH_ACETYLATION | 0.107 |
| 426 | REACTOME_DEFECTIVE_CFTR_CAUSES_CYSTIC_FIBROSIS | 0.106 |
| 427 | REACTOME_ASSEMBLY_OF_THE_HIV_VIRION | 0.106 |
| 428 | REACTOME_HDL_ASSEMBLY | 0.105 |
| 429 | REACTOME_ALPHA_DEFENSINS | 0.105 |
| 430 | REACTOME_DEX_H_BOX_HELICASES_ACTIVATE_TYPE_I_IFN_AND_INFLAMMATORY_CYTOKINES_PRODUCTION | 0.105 |
| 431 | REACTOME_PHOSPHORYLATION_OF_EMI1 | 0.104 |
| 432 | REACTOME_WNT_MEDIATED_ACTIVATION_OF_DVL | 0.104 |
| 433 | REACTOME_RHOD_GTPASE_CYCLE | 0.104 |
| 434 | REACTOME_MATURATION_OF_SARSCOV1_NUCLEOPROTEIN | 0.104 |
| 435 | REACTOME_ADHERENS_JUNCTIONS_INTERACTIONS | 0.104 |
| 436 | REACTOME_INITIATION_OF_NUCLEAR_ENVELOPE_NE_REFORMATION | 0.103 |
| 437 | REACTOME_FRUCTOSE_CATABOLISM | 0.103 |
| 438 | REACTOME_INSULIN_RECEPTOR_RECYCLING | 0.103 |
| 439 | REACTOME_GENE_AND_PROTEIN_EXPRESSION_BY_JAK_STAT_SIGNALING_AFTER_INTERLEUKIN_12_STIMULATION | 0.102 |
| 440 | REACTOME_INACTIVATION_OF_CSF3_G_CSF_SIGNALING | 0.102 |
| 441 | REACTOME_SYNTHESIS_OF_15_EICOSATETRAENOIC_ACID_DERIVATIVES | 0.102 |
| 442 | REACTOME_NEGATIVE_REGULATORS_OF_DDX58_IFIH1_SIGNALING | 0.102 |
| 443 | REACTOME_TRAF6_MEDIATED_IRF7_ACTIVATION_IN_TLR7_8_OR_9_SIGNALING | 0.101 |
| 444 | REACTOME_PHASE_1_INACTIVATION_OF_FAST_NA_CHANNELS | 0.101 |
| 445 | REACTOME_LYSOSPHINGOLIPID_AND_LPA_RECEPTORS | 0.101 |
| 446 | REACTOME_REGULATION_OF_PTEN_LOCALIZATION | 0.100 |
| 447 | REACTOME_EARLY_SARS_COV_2_INFECTION_EVENTS | 0.100 |
| 448 | REACTOME_LTC4_CYSLTR_MEDIATED_IL4_PRODUCTION | 0.100 |
| 449 | REACTOME_RESOLUTION_OF_D_LOOP_STRUCTURES | 0.100 |
| 450 | REACTOME_METABOLISM_OF_ANGIOTENSINOGEN_TO_ANGIOTENSINS | 0.100 |
| 451 | REACTOME_ACTIVATED_NTRK2_SIGNALS_THROUGH_FRS2_AND_FRS3 | 0.100 |
| 452 | REACTOME_DOWNREGULATION_OF_SMAD2_3_SMAD4_TRANSCRIPTIONAL_ACTIVITY | 0.099 |
| 453 | REACTOME_NUCLEAR_ENVELOPE_BREAKDOWN | 0.099 |
| 454 | REACTOME_RECEPTOR_MEDIATED_MITOPHAGY | 0.099 |
| 455 | REACTOME_JNK_C_JUN_KINASES_PHOSPHORYLATION_AND_ACTIVATION_MEDIATED_BY_ACTIVATED_HUMAN_TAK1 | 0.099 |
| 456 | REACTOME_BETA_CATENIN_PHOSPHORYLATION_CASCADE | 0.098 |
| 457 | REACTOME_SHC1_EVENTS_IN_ERBB4_SIGNALING | 0.098 |
| 458 | REACTOME_DOWNREGULATION_OF_ERBB2_ERBB3_SIGNALING | 0.098 |
| 459 | REACTOME_TRIGLYCERIDE_CATABOLISM | 0.097 |
| 460 | REACTOME_HYALURONAN_UPTAKE_AND_DEGRADATION | 0.097 |
| 461 | REACTOME_THE_CANONICAL_RETINOID_CYCLE_IN_RODS_TWILIGHT_VISION | 0.097 |
| 462 | REACTOME_GLUCAGON_TYPE_LIGAND_RECEPTORS | 0.097 |
| 463 | REACTOME_RESOLUTION_OF_D_LOOP_STRUCTURES_THROUGH_SYNTHESIS_DEPENDENT_STRAND_ANNEALING_SDSA | 0.096 |
| 464 | REACTOME_ALK_MUTANTS_BIND_TKIS | 0.096 |
| 465 | REACTOME_NCAM1_INTERACTIONS | 0.096 |
| 466 | REACTOME_IRS_ACTIVATION | 0.096 |
| 467 | REACTOME_METABOLIC_DISORDERS_OF_BIOLOGICAL_OXIDATION_ENZYMES | 0.096 |
| 468 | REACTOME_GABA_SYNTHESIS_RELEASE_REUPTAKE_AND_DEGRADATION | 0.094 |
| 469 | REACTOME_ACTIVATION_OF_ATR_IN_RESPONSE_TO_REPLICATION_STRESS | 0.094 |
| 470 | REACTOME_ACTIVATION_OF_THE_PHOTOTRANSDUCTION_CASCADE | 0.094 |
| 471 | REACTOME_TRAFFICKING_OF_GLUR2_CONTAINING_AMPA_RECEPTORS | 0.094 |
| 472 | REACTOME_DISINHIBITION_OF_SNARE_FORMATION | 0.094 |
| 473 | REACTOME_KSRP_KHSRP_BINDS_AND_DESTABILIZES_MRNA | 0.093 |
| 474 | REACTOME_PEXOPHAGY | 0.092 |
| 475 | REACTOME_INTERLEUKIN_21_SIGNALING | 0.092 |
| 476 | REACTOME_COPI_INDEPENDENT_GOLGI_TO_ER_RETROGRADE_TRAFFIC | 0.091 |
| 477 | REACTOME_AGGREPHAGY | 0.091 |
| 478 | REACTOME_GLYCOSPHINGOLIPID_METABOLISM | 0.091 |
| 479 | REACTOME_NEF_MEDIATED_CD4_DOWN_REGULATION | 0.090 |
| 480 | REACTOME_INTERLEUKIN_15_SIGNALING | 0.090 |
| 481 | REACTOME_RECEPTOR_TYPE_TYROSINE_PROTEIN_PHOSPHATASES | 0.089 |
| 482 | REACTOME_RELEASE_OF_HH_NP_FROM_THE_SECRETING_CELL | 0.089 |
| 483 | REACTOME_BASIGIN_INTERACTIONS | 0.089 |
| 484 | REACTOME_CASPASE_MEDIATED_CLEAVAGE_OF_CYTOSKELETAL_PROTEINS | 0.088 |
| 485 | REACTOME_BMAL1_CLOCK_NPAS2_ACTIVATES_CIRCADIAN_GENE_EXPRESSION | 0.088 |
| 486 | REACTOME_REGULATION_BY_C_FLIP | 0.088 |
| 487 | REACTOME_JOSEPHIN_DOMAIN_DUBS | 0.087 |
| 488 | REACTOME_SUMOYLATION_OF_IMMUNE_RESPONSE_PROTEINS | 0.087 |
| 489 | REACTOME_PRESYNAPTIC_FUNCTION_OF_KAINATE_RECEPTORS | 0.087 |
| 490 | REACTOME_UPTAKE_AND_FUNCTION_OF_ANTHRAX_TOXINS | 0.087 |
| 491 | REACTOME_REGULATION_OF_SIGNALING_BY_CBL | 0.087 |
| 492 | REACTOME_DISEASES_ASSOCIATED_WITH_GLYCOSYLATION_PRECURSOR_BIOSYNTHESIS | 0.086 |
| 493 | REACTOME_PLASMA_LIPOPROTEIN_REMODELING | 0.085 |
| 494 | REACTOME_LIGAND_RECEPTOR_INTERACTIONS | 0.085 |
| 495 | REACTOME_CYCLIN_A_B1_B2_ASSOCIATED_EVENTS_DURING_G2_M_TRANSITION | 0.084 |
| 496 | REACTOME_P2Y_RECEPTORS | 0.084 |
| 497 | REACTOME_TRANSFERRIN_ENDOCYTOSIS_AND_RECYCLING | 0.084 |
| 498 | REACTOME_MITOTIC_TELOPHASE_CYTOKINESIS | 0.084 |
| 499 | REACTOME_GAP_JUNCTION_DEGRADATION | 0.084 |
| 500 | REACTOME_TNF_SIGNALING | 0.084 |
| 501 | REACTOME_TNFR1_INDUCED_NFKAPPAB_SIGNALING_PATHWAY | 0.084 |
| 502 | REACTOME_ERYTHROCYTES_TAKE_UP_OXYGEN_AND_RELEASE_CARBON_DIOXIDE | 0.083 |
| 503 | REACTOME_TRANSLATION_OF_SARSCOV2_STRUCTURAL_PROTEINS | 0.083 |
| 504 | REACTOME_ELASTIC_FIBRE_FORMATION | 0.083 |
| 505 | REACTOME_DECTIN_1_MEDIATED_NONCANONICAL_NF_KB_SIGNALING | 0.083 |
| 506 | REACTOME_DEFECTIVE_CSF2RB_CAUSES_SMDP5 | 0.082 |
| 507 | REACTOME_DNA_DAMAGE_BYPASS | 0.082 |
| 508 | REACTOME_THE_ACTIVATION_OF_ARYLSULFATASES | 0.081 |
| 509 | REACTOME_CHK1_CHK2_CDS1_MEDIATED_INACTIVATION_OF_CYCLIN_B_CDK1_COMPLEX | 0.081 |
| 510 | REACTOME_CATION_COUPLED_CHLORIDE_COTRANSPORTERS | 0.081 |
| 511 | REACTOME_SYNTHESIS_OF_PIPS_AT_THE_ER_MEMBRANE | 0.081 |
| 512 | REACTOME_ASSEMBLY_OF_ACTIVE_LPL_AND_LIPC_LIPASE_COMPLEXES | 0.080 |
| 513 | REACTOME_TP53_REGULATES_TRANSCRIPTION_OF_GENES_INVOLVED_IN_CYTOCHROME_C_RELEASE | 0.080 |
| 514 | REACTOME_MECP2_REGULATES_TRANSCRIPTION_OF_NEURONAL_LIGANDS | 0.080 |
| 515 | REACTOME_CRISTAE_FORMATION | 0.080 |
| 516 | REACTOME_DOWNREGULATION_OF_TGF_BETA_RECEPTOR_SIGNALING | 0.080 |
| 517 | REACTOME_INTERLEUKIN_27_SIGNALING | 0.080 |
| 518 | REACTOME_METABOLISM_OF_INGESTED_SEMET_SEC_MESEC_INTO_H2SE | 0.080 |
| 519 | REACTOME_CELL_CELL_JUNCTION_ORGANIZATION | 0.080 |
| 520 | REACTOME_GLUCAGON_SIGNALING_IN_METABOLIC_REGULATION | 0.079 |
| 521 | REACTOME_CALCITONIN_LIKE_LIGAND_RECEPTORS | 0.079 |
| 522 | REACTOME_COOPERATION_OF_PDCL_PHLP1_AND_TRIC_CCT_IN_G_PROTEIN_BETA_FOLDING | 0.078 |
| 523 | REACTOME_PRE_NOTCH_PROCESSING_IN_GOLGI | 0.078 |
| 524 | REACTOME_INTERACTIONS_OF_REV_WITH_HOST_CELLULAR_PROTEINS | 0.078 |
| 525 | REACTOME_HEDGEHOG_LIGAND_BIOGENESIS | 0.078 |
| 526 | REACTOME_REGULATION_OF_GLUCOKINASE_BY_GLUCOKINASE_REGULATORY_PROTEIN | 0.077 |
| 527 | REACTOME_P75NTR_NEGATIVELY_REGULATES_CELL_CYCLE_VIA_SC1 | 0.076 |
| 528 | REACTOME_RECYCLING_PATHWAY_OF_L1 | 0.076 |
| 529 | REACTOME_CHOLINE_CATABOLISM | 0.076 |
| 530 | REACTOME_FASL_CD95L_SIGNALING | 0.075 |
| 531 | REACTOME_MET_ACTIVATES_PI3K_AKT_SIGNALING | 0.075 |
| 532 | REACTOME_NRAGE_SIGNALS_DEATH_THROUGH_JNK | 0.075 |
| 533 | REACTOME_CTLA4_INHIBITORY_SIGNALING | 0.075 |
| 534 | REACTOME_DEFECTIVE_INTRINSIC_PATHWAY_FOR_APOPTOSIS | 0.075 |
| 535 | REACTOME_ROBO_RECEPTORS_BIND_AKAP5 | 0.075 |
| 536 | REACTOME_RUNX1_REGULATES_TRANSCRIPTION_OF_GENES_INVOLVED_IN_INTERLEUKIN_SIGNALING | 0.074 |
| 537 | REACTOME_NOTCH2_ACTIVATION_AND_TRANSMISSION_OF_SIGNAL_TO_THE_NUCLEUS | 0.074 |
| 538 | REACTOME_SIGNALING_BY_NOTCH2 | 0.073 |
| 539 | REACTOME_UPTAKE_AND_ACTIONS_OF_BACTERIAL_TOXINS | 0.073 |
| 540 | REACTOME_INTERLEUKIN_2_SIGNALING | 0.073 |
| 541 | REACTOME_HSF1_ACTIVATION | 0.073 |
| 542 | REACTOME_ROLE_OF_SECOND_MESSENGERS_IN_NETRIN_1_SIGNALING | 0.073 |
| 543 | REACTOME_RUNX1_REGULATES_TRANSCRIPTION_OF_GENES_INVOLVED_IN_DIFFERENTIATION_OF_MYELOID_CELLS | 0.073 |
| 544 | REACTOME_TACHYKININ_RECEPTORS_BIND_TACHYKININS | 0.072 |
| 545 | REACTOME_NUCLEOTIDE_SALVAGE | 0.072 |
| 546 | REACTOME_RSK_ACTIVATION | 0.072 |
| 547 | REACTOME_CA2_ACTIVATED_K_CHANNELS | 0.072 |
| 548 | REACTOME_AMPK_INHIBITS_CHREBP_TRANSCRIPTIONAL_ACTIVATION_ACTIVITY | 0.072 |
| 549 | REACTOME_SHC_RELATED_EVENTS_TRIGGERED_BY_IGF1R | 0.071 |
| 550 | REACTOME_EPHRIN_SIGNALING | 0.071 |
| 551 | REACTOME_TRANSPORT_OF_NUCLEOSIDES_AND_FREE_PURINE_AND_PYRIMIDINE_BASES_ACROSS_THE_PLASMA_MEMBRANE | 0.071 |
| 552 | REACTOME_PTK6_REGULATES_RHO_GTPASES_RAS_GTPASE_AND_MAP_KINASES | 0.071 |
| 553 | REACTOME_DAG_AND_IP3_SIGNALING | 0.071 |
| 554 | REACTOME_SYNTHESIS_SECRETION_AND_INACTIVATION_OF_GLUCAGON_LIKE_PEPTIDE_1_GLP_1 | 0.071 |
| 555 | REACTOME_PHASE_2_PLATEAU_PHASE | 0.071 |
| 556 | REACTOME_LDL_CLEARANCE | 0.070 |
| 557 | REACTOME_TRANSPORT_OF_CONNEXONS_TO_THE_PLASMA_MEMBRANE | 0.070 |
| 558 | REACTOME_FGFR3B_LIGAND_BINDING_AND_ACTIVATION | 0.070 |
| 559 | REACTOME_TP53_REGULATES_TRANSCRIPTION_OF_CASPASE_ACTIVATORS_AND_CASPASES | 0.069 |
| 560 | REACTOME_REGULATION_OF_GENE_EXPRESSION_BY_HYPOXIA_INDUCIBLE_FACTOR | 0.069 |
| 561 | REACTOME_ACTIVATED_NOTCH1_TRANSMITS_SIGNAL_TO_THE_NUCLEUS | 0.068 |
| 562 | REACTOME_SUMOYLATION_OF_TRANSCRIPTION_FACTORS | 0.068 |
| 563 | REACTOME_GLI_PROTEINS_BIND_PROMOTERS_OF_HH_RESPONSIVE_GENES_TO_PROMOTE_TRANSCRIPTION | 0.068 |
| 564 | REACTOME_LYSOSOME_VESICLE_BIOGENESIS | 0.068 |
| 565 | REACTOME_RELEASE_OF_APOPTOTIC_FACTORS_FROM_THE_MITOCHONDRIA | 0.068 |
| 566 | REACTOME_TRANSCRIPTIONAL_REGULATION_OF_TESTIS_DIFFERENTIATION | 0.067 |
| 567 | REACTOME_REGULATION_OF_LOCALIZATION_OF_FOXO_TRANSCRIPTION_FACTORS | 0.067 |
| 568 | REACTOME_IRON_UPTAKE_AND_TRANSPORT | 0.066 |
| 569 | REACTOME_BRANCHED_CHAIN_AMINO_ACID_CATABOLISM | 0.066 |
| 570 | REACTOME_SEALING_OF_THE_NUCLEAR_ENVELOPE_NE_BY_ESCRT_III | 0.065 |
| 571 | REACTOME_SYNTHESIS_OF_5_EICOSATETRAENOIC_ACIDS | 0.065 |
| 572 | REACTOME_PHOSPHORYLATION_OF_THE_APC_C | 0.065 |
| 573 | REACTOME_NONCANONICAL_ACTIVATION_OF_NOTCH3 | 0.065 |
| 574 | REACTOME_THE_NLRP3_INFLAMMASOME | 0.065 |
| 575 | REACTOME_NEPHRIN_FAMILY_INTERACTIONS | 0.065 |
| 576 | REACTOME_NETRIN_MEDIATED_REPULSION_SIGNALS | 0.065 |
| 577 | REACTOME_ROLE_OF_ABL_IN_ROBO_SLIT_SIGNALING | 0.064 |
| 578 | REACTOME_CREB_PHOSPHORYLATION | 0.064 |
| 579 | REACTOME_SIGNALING_BY_NOTCH1_PEST_DOMAIN_MUTANTS_IN_CANCER | 0.064 |
| 580 | REACTOME_VASOPRESSIN_LIKE_RECEPTORS | 0.063 |
| 581 | REACTOME_SYNTHESIS_OF_12_EICOSATETRAENOIC_ACID_DERIVATIVES | 0.063 |
| 582 | REACTOME_PECAM1_INTERACTIONS | 0.063 |
| 583 | REACTOME_SENSORY_PROCESSING_OF_SOUND_BY_OUTER_HAIR_CELLS_OF_THE_COCHLEA | 0.063 |
| 584 | REACTOME_GLUTAMATE_AND_GLUTAMINE_METABOLISM | 0.063 |
| 585 | REACTOME_REGULATION_OF_BETA_CELL_DEVELOPMENT | 0.063 |
| 586 | REACTOME_GOLGI_ASSOCIATED_VESICLE_BIOGENESIS | 0.063 |
| 587 | REACTOME_IONOTROPIC_ACTIVITY_OF_KAINATE_RECEPTORS | 0.062 |
| 588 | REACTOME_SENSING_OF_DNA_DOUBLE_STRAND_BREAKS | 0.061 |
| 589 | REACTOME_DIGESTION_OF_DIETARY_LIPID | 0.061 |
| 590 | REACTOME_REGULATION_OF_PTEN_MRNA_TRANSLATION | 0.060 |
| 591 | REACTOME_VXPX_CARGO_TARGETING_TO_CILIUM | 0.060 |
| 592 | REACTOME_MINERALOCORTICOID_BIOSYNTHESIS | 0.060 |
| 593 | REACTOME_DECTIN_2_FAMILY | 0.059 |
| 594 | REACTOME_ETHANOL_OXIDATION | 0.059 |
| 595 | REACTOME_THE_RETINOID_CYCLE_IN_CONES_DAYLIGHT_VISION | 0.059 |
| 596 | REACTOME_NOREPINEPHRINE_NEUROTRANSMITTER_RELEASE_CYCLE | 0.059 |
| 597 | REACTOME_TRAFFICKING_AND_PROCESSING_OF_ENDOSOMAL_TLR | 0.059 |
| 598 | REACTOME_TRYPTOPHAN_CATABOLISM | 0.059 |
| 599 | REACTOME_KETONE_BODY_METABOLISM | 0.059 |
| 600 | REACTOME_KERATAN_SULFATE_DEGRADATION | 0.058 |
| 601 | REACTOME_VITAMINS | 0.058 |
| 602 | REACTOME_ACTIVATED_NTRK2_SIGNALS_THROUGH_FYN | 0.058 |
| 603 | REACTOME_FGFR3_LIGAND_BINDING_AND_ACTIVATION | 0.058 |
| 604 | REACTOME_ASSOCIATION_OF_TRIC_CCT_WITH_TARGET_PROTEINS_DURING_BIOSYNTHESIS | 0.058 |
| 605 | REACTOME_PERK_REGULATES_GENE_EXPRESSION | 0.058 |
| 606 | REACTOME_REGULATION_OF_SIGNALING_BY_NODAL | 0.058 |
| 607 | REACTOME_BINDING_OF_TCF_LEF_CTNNB1_TO_TARGET_GENE_PROMOTERS | 0.057 |
| 608 | REACTOME_SYNTHESIS_OF_PIPS_AT_THE_GOLGI_MEMBRANE | 0.057 |
| 609 | REACTOME_CHL1_INTERACTIONS | 0.057 |
| 610 | REACTOME_KERATAN_SULFATE_KERATIN_METABOLISM | 0.057 |
| 611 | REACTOME_EPH_EPHRIN_MEDIATED_REPULSION_OF_CELLS | 0.057 |
| 612 | REACTOME_CRMPS_IN_SEMA3A_SIGNALING | 0.056 |
| 613 | REACTOME_FATTY_ACIDS_BOUND_TO_GPR40_FFAR1_REGULATE_INSULIN_SECRETION | 0.056 |
| 614 | REACTOME_GLUTATHIONE_CONJUGATION | 0.056 |
| 615 | REACTOME_SCAVENGING_BY_CLASS_A_RECEPTORS | 0.056 |
| 616 | REACTOME_DARPP_32_EVENTS | 0.056 |
| 617 | REACTOME_VLDL_CLEARANCE | 0.055 |
| 618 | REACTOME_UREA_CYCLE | 0.055 |
| 619 | REACTOME_DISSOLUTION_OF_FIBRIN_CLOT | 0.055 |
| 620 | REACTOME_PHYSIOLOGICAL_FACTORS | 0.055 |
| 621 | REACTOME_NEUROTRANSMITTER_RELEASE_CYCLE | 0.054 |
| 622 | REACTOME_ELEVATION_OF_CYTOSOLIC_CA2_LEVELS | 0.054 |
| 623 | REACTOME_ESTROGEN_BIOSYNTHESIS | 0.054 |
| 624 | REACTOME_SCAVENGING_BY_CLASS_B_RECEPTORS | 0.054 |
| 625 | REACTOME_PRESYNAPTIC_DEPOLARIZATION_AND_CALCIUM_CHANNEL_OPENING | 0.054 |
| 626 | REACTOME_MICRORNA_MIRNA_BIOGENESIS | 0.053 |
| 627 | REACTOME_RUNX1_INTERACTS_WITH_CO_FACTORS_WHOSE_PRECISE_EFFECT_ON_RUNX1_TARGETS_IS_NOT_KNOWN | 0.053 |
| 628 | REACTOME_DOPAMINE_CLEARANCE_FROM_THE_SYNAPTIC_CLEFT | 0.053 |
| 629 | REACTOME_AQUAPORIN_MEDIATED_TRANSPORT | 0.053 |
| 630 | REACTOME_REGULATION_OF_GENE_EXPRESSION_IN_BETA_CELLS | 0.053 |
| 631 | REACTOME_DEGRADATION_OF_GLI1_BY_THE_PROTEASOME | 0.052 |
| 632 | REACTOME_RIPK1_MEDIATED_REGULATED_NECROSIS | 0.052 |
| 633 | REACTOME_TOXICITY_OF_BOTULINUM_TOXIN_TYPE_D_BOTD | 0.052 |
| 634 | REACTOME_LEUKOTRIENE_RECEPTORS | 0.052 |
| 635 | REACTOME_ORGANIC_ANION_TRANSPORTERS | 0.052 |
| 636 | REACTOME_TFAP2_AP_2_FAMILY_REGULATES_TRANSCRIPTION_OF_CELL_CYCLE_FACTORS | 0.052 |
| 637 | REACTOME_MATURATION_OF_SARSCOV2_NUCLEOPROTEIN | 0.052 |
| 638 | REACTOME_PEROXISOMAL_PROTEIN_IMPORT | 0.051 |
| 639 | REACTOME_HDL_REMODELING | 0.051 |
| 640 | REACTOME_ACETYLCHOLINE_BINDING_AND_DOWNSTREAM_EVENTS | 0.051 |
| 641 | REACTOME_SEMA3A_PAK_DEPENDENT_AXON_REPULSION | 0.051 |
| 642 | REACTOME_NUCLEOTIDE_CATABOLISM | 0.050 |
| 643 | REACTOME_PTK6_REGULATES_RTKS_AND_THEIR_EFFECTORS_AKT1_AND_DOK1 | 0.050 |
| 644 | REACTOME_PYRIMIDINE_SALVAGE | 0.050 |
| 645 | REACTOME_AKT_PHOSPHORYLATES_TARGETS_IN_THE_NUCLEUS | 0.050 |
| 646 | REACTOME_RAS_PROCESSING | 0.049 |
| 647 | REACTOME_REGULATION_OF_TP53_ACTIVITY_THROUGH_ASSOCIATION_WITH_CO_FACTORS | 0.049 |
| 648 | REACTOME_ION_TRANSPORT_BY_P_TYPE_ATPASES | 0.049 |
| 649 | REACTOME_RUNX3_REGULATES_CDKN1A_TRANSCRIPTION | 0.049 |
| 650 | REACTOME_FORMATION_OF_SENESCENCE_ASSOCIATED_HETEROCHROMATIN_FOCI_SAHF | 0.048 |
| 651 | REACTOME_GAP_JUNCTION_TRAFFICKING_AND_REGULATION | 0.048 |
| 652 | REACTOME_MET_INTERACTS_WITH_TNS_PROTEINS | 0.048 |
| 653 | REACTOME_HIGHLY_CALCIUM_PERMEABLE_POSTSYNAPTIC_NICOTINIC_ACETYLCHOLINE_RECEPTORS | 0.048 |
| 654 | REACTOME_CYCLIN_D_ASSOCIATED_EVENTS_IN_G1 | 0.048 |
| 655 | REACTOME_ANDROGEN_BIOSYNTHESIS | 0.048 |
| 656 | REACTOME_ORGANIC_CATION_TRANSPORT | 0.048 |
| 657 | REACTOME_DEFECTIVE_F9_ACTIVATION | 0.048 |
| 658 | REACTOME_MET_ACTIVATES_PTK2_SIGNALING | 0.047 |
| 659 | REACTOME_INTRA_GOLGI_TRAFFIC | 0.047 |
| 660 | REACTOME_SPHINGOLIPID_DE_NOVO_BIOSYNTHESIS | 0.047 |
| 661 | REACTOME_PURINE_CATABOLISM | 0.047 |
| 662 | REACTOME_THROMBOXANE_SIGNALLING_THROUGH_TP_RECEPTOR | 0.047 |
| 663 | REACTOME_INSULIN_PROCESSING | 0.046 |
| 664 | REACTOME_ATF4_ACTIVATES_GENES_IN_RESPONSE_TO_ENDOPLASMIC_RETICULUM_STRESS | 0.046 |
| 665 | REACTOME_ASSEMBLY_OF_COLLAGEN_FIBRILS_AND_OTHER_MULTIMERIC_STRUCTURES | 0.046 |
| 666 | REACTOME_LATE_ENDOSOMAL_MICROAUTOPHAGY | 0.046 |
| 667 | REACTOME_METABOLISM_OF_POLYAMINES | 0.045 |
| 668 | REACTOME_VITAMIN_B2_RIBOFLAVIN_METABOLISM | 0.045 |
| 669 | REACTOME_DCC_MEDIATED_ATTRACTIVE_SIGNALING | 0.045 |
| 670 | REACTOME_FORMATION_OF_TC_NER_PRE_INCISION_COMPLEX | 0.045 |
| 671 | REACTOME_REGULATION_OF_RUNX3_EXPRESSION_AND_ACTIVITY | 0.045 |
| 672 | REACTOME_SCAVENGING_BY_CLASS_F_RECEPTORS | 0.045 |
| 673 | REACTOME_LAMININ_INTERACTIONS | 0.044 |
| 674 | REACTOME_OPSINS | 0.044 |
| 675 | REACTOME_GLUCURONIDATION | 0.044 |
| 676 | REACTOME_TP53_REGULATES_TRANSCRIPTION_OF_ADDITIONAL_CELL_CYCLE_GENES_WHOSE_EXACT_ROLE_IN_THE_P53_PATHWAY_REMAIN_UNCERTAIN | 0.044 |
| 677 | REACTOME_PROLACTIN_RECEPTOR_SIGNALING | 0.044 |
| 678 | REACTOME_ERYTHROPOIETIN_ACTIVATES_RAS | 0.043 |
| 679 | REACTOME_INTERLEUKIN_18_SIGNALING | 0.043 |
| 680 | REACTOME_NOTCH_HLH_TRANSCRIPTION_PATHWAY | 0.043 |
| 681 | REACTOME_VLDL_ASSEMBLY | 0.042 |
| 682 | REACTOME_HYALURONAN_METABOLISM | 0.042 |
| 683 | REACTOME_NGF_STIMULATED_TRANSCRIPTION | 0.042 |
| 684 | REACTOME_FORMATION_OF_ATP_BY_CHEMIOSMOTIC_COUPLING | 0.042 |
| 685 | REACTOME_RUNX3_REGULATES_NOTCH_SIGNALING | 0.042 |
| 686 | REACTOME_SIGNALING_BY_BMP | 0.042 |
| 687 | REACTOME_EXTENSION_OF_TELOMERES | 0.041 |
| 688 | REACTOME_ERYTHROCYTES_TAKE_UP_CARBON_DIOXIDE_AND_RELEASE_OXYGEN | 0.041 |
| 689 | REACTOME_REGULATION_OF_FOXO_TRANSCRIPTIONAL_ACTIVITY_BY_ACETYLATION | 0.041 |
| 690 | REACTOME_SYNTHESIS_OF_LIPOXINS_LX | 0.041 |
| 691 | REACTOME_LIPOPHAGY | 0.041 |
| 692 | REACTOME_CHYLOMICRON_CLEARANCE | 0.041 |
| 693 | REACTOME_RRNA_MODIFICATION_IN_THE_NUCLEUS_AND_CYTOSOL | 0.040 |
| 694 | REACTOME_ACTIVATION_OF_NOXA_AND_TRANSLOCATION_TO_MITOCHONDRIA | 0.040 |
| 695 | REACTOME_PHASE_4_RESTING_MEMBRANE_POTENTIAL | 0.040 |
| 696 | REACTOME_CALCINEURIN_ACTIVATES_NFAT | 0.040 |
| 697 | REACTOME_RNA_POLYMERASE_I_TRANSCRIPTION_INITIATION | 0.040 |
| 698 | REACTOME_IL_6_TYPE_CYTOKINE_RECEPTOR_LIGAND_INTERACTIONS | 0.039 |
| 699 | REACTOME_SYNTHESIS_OF_PE | 0.039 |
| 700 | REACTOME_NEGATIVE_REGULATION_OF_NOTCH4_SIGNALING | 0.039 |
| 701 | REACTOME_CHYLOMICRON_REMODELING | 0.039 |
| 702 | REACTOME_INSERTION_OF_TAIL_ANCHORED_PROTEINS_INTO_THE_ENDOPLASMIC_RETICULUM_MEMBRANE | 0.039 |
| 703 | REACTOME_REVERSIBLE_HYDRATION_OF_CARBON_DIOXIDE | 0.039 |
| 704 | REACTOME_DNA_METHYLATION | 0.039 |
| 705 | REACTOME_TRIGLYCERIDE_BIOSYNTHESIS | 0.039 |
| 706 | REACTOME_CLEC7A_DECTIN_1_INDUCES_NFAT_ACTIVATION | 0.039 |
| 707 | REACTOME_REGULATION_OF_BACH1_ACTIVITY | 0.039 |
| 708 | REACTOME_POSTMITOTIC_NUCLEAR_PORE_COMPLEX_NPC_REFORMATION | 0.038 |
| 709 | REACTOME_TANDEM_PORE_DOMAIN_POTASSIUM_CHANNELS | 0.038 |
| 710 | REACTOME_COMPLEX_I_BIOGENESIS | 0.038 |
| 711 | REACTOME_LDL_REMODELING | 0.038 |
| 712 | REACTOME_NEF_AND_SIGNAL_TRANSDUCTION | 0.038 |
| 713 | REACTOME_SUPPRESSION_OF_APOPTOSIS | 0.038 |
| 714 | REACTOME_INTEGRATION_OF_PROVIRUS | 0.038 |
| 715 | REACTOME_NOTCH2_INTRACELLULAR_DOMAIN_REGULATES_TRANSCRIPTION | 0.038 |
| 716 | REACTOME_UPTAKE_AND_FUNCTION_OF_DIPHTHERIA_TOXIN | 0.038 |
| 717 | REACTOME_MITOPHAGY | 0.038 |
| 718 | REACTOME_PLATELET_ADHESION_TO_EXPOSED_COLLAGEN | 0.037 |
| 719 | REACTOME_TRANSPORT_OF_RCBL_WITHIN_THE_BODY | 0.037 |
| 720 | REACTOME_BUTYRATE_RESPONSE_FACTOR_1_BRF1_BINDS_AND_DESTABILIZES_MRNA | 0.037 |
| 721 | REACTOME_TRANSLESION_SYNTHESIS_BY_POLH | 0.037 |
| 722 | REACTOME_CELL_EXTRACELLULAR_MATRIX_INTERACTIONS | 0.037 |
| 723 | REACTOME_ACETYLCHOLINE_INHIBITS_CONTRACTION_OF_OUTER_HAIR_CELLS | 0.036 |
| 724 | REACTOME_ERYTHROPOIETIN_ACTIVATES_PHOSPHOLIPASE_C_GAMMA_PLCG | 0.036 |
| 725 | REACTOME_PKA_MEDIATED_PHOSPHORYLATION_OF_KEY_METABOLIC_FACTORS | 0.036 |
| 726 | REACTOME_ATF6_ATF6_ALPHA_ACTIVATES_CHAPERONE_GENES | 0.035 |
| 727 | REACTOME_CROSS_PRESENTATION_OF_SOLUBLE_EXOGENOUS_ANTIGENS_ENDOSOMES | 0.035 |
| 728 | REACTOME_GRB7_EVENTS_IN_ERBB2_SIGNALING | 0.035 |
| 729 | REACTOME_HDL_CLEARANCE | 0.035 |
| 730 | REACTOME_FREE_FATTY_ACID_RECEPTORS | 0.035 |
| 731 | REACTOME_HDMS_DEMETHYLATE_HISTONES | 0.035 |
| 732 | REACTOME_SARS_COV_2_TARGETS_PDZ_PROTEINS_IN_CELL_CELL_JUNCTION | 0.035 |
| 733 | REACTOME_SARS_COV_2_TARGETS_HOST_INTRACELLULAR_SIGNALLING_AND_REGULATORY_PATHWAYS | 0.035 |
| 734 | REACTOME_DETOXIFICATION_OF_REACTIVE_OXYGEN_SPECIES | 0.035 |
| 735 | REACTOME_DISEASES_ASSOCIATED_WITH_GLYCOSAMINOGLYCAN_METABOLISM | 0.035 |
| 736 | REACTOME_NUCLEAR_IMPORT_OF_REV_PROTEIN | 0.035 |
| 737 | REACTOME_DIGESTION_AND_ABSORPTION | 0.034 |
| 738 | REACTOME_INLA_MEDIATED_ENTRY_OF_LISTERIA_MONOCYTOGENES_INTO_HOST_CELLS | 0.033 |
| 739 | REACTOME_BUTYROPHILIN_BTN_FAMILY_INTERACTIONS | 0.033 |
| 740 | REACTOME_CD22_MEDIATED_BCR_REGULATION | 0.033 |
| 741 | REACTOME_MOLECULES_ASSOCIATED_WITH_ELASTIC_FIBRES | 0.032 |
| 742 | REACTOME_HIV_TRANSCRIPTION_INITIATION | 0.032 |
| 743 | REACTOME_PIWI_INTERACTING_RNA_PIRNA_BIOGENESIS | 0.032 |
| 744 | REACTOME_SUMOYLATION_OF_UBIQUITINYLATION_PROTEINS | 0.031 |
| 745 | REACTOME_ACTIVATED_NTRK2_SIGNALS_THROUGH_RAS | 0.031 |
| 746 | REACTOME_ACTIVATION_OF_THE_MRNA_UPON_BINDING_OF_THE_CAP_BINDING_COMPLEX_AND_EIFS_AND_SUBSEQUENT_BINDING_TO_43S | 0.031 |
| 747 | REACTOME_SEMA4D_MEDIATED_INHIBITION_OF_CELL_ATTACHMENT_AND_MIGRATION | 0.031 |
| 748 | REACTOME_RHOH_GTPASE_CYCLE | 0.030 |
| 749 | REACTOME_FERTILIZATION | 0.030 |
| 750 | REACTOME_REPRESSION_OF_WNT_TARGET_GENES | 0.030 |
| 751 | REACTOME_CELLULAR_HEXOSE_TRANSPORT | 0.030 |
| 752 | REACTOME_FOXO_MEDIATED_TRANSCRIPTION_OF_CELL_CYCLE_GENES | 0.030 |
| 753 | REACTOME_COOPERATION_OF_PREFOLDIN_AND_TRIC_CCT_IN_ACTIN_AND_TUBULIN_FOLDING | 0.030 |
| 754 | REACTOME_O_GLYCOSYLATION_OF_TSR_DOMAIN_CONTAINING_PROTEINS | 0.030 |
| 755 | REACTOME_METABOLISM_OF_COFACTORS | 0.030 |
| 756 | REACTOME_KINESINS | 0.030 |
| 757 | REACTOME_YAP1_AND_WWTR1_TAZ_STIMULATED_GENE_EXPRESSION | 0.030 |
| 758 | REACTOME_CD28_DEPENDENT_VAV1_PATHWAY | 0.029 |
| 759 | REACTOME_TP53_REGULATES_TRANSCRIPTION_OF_DEATH_RECEPTORS_AND_LIGANDS | 0.029 |
| 760 | REACTOME_TFAP2A_ACTS_AS_A_TRANSCRIPTIONAL_REPRESSOR_DURING_RETINOIC_ACID_INDUCED_CELL_DIFFERENTIATION | 0.029 |
| 761 | REACTOME_LYSINE_CATABOLISM | 0.028 |
| 762 | REACTOME_SARS_COV_2_MODULATES_HOST_TRANSLATION_MACHINERY | 0.028 |
| 763 | REACTOME_ROS_AND_RNS_PRODUCTION_IN_PHAGOCYTES | 0.028 |
| 764 | REACTOME_ACTIVATION_OF_AMPK_DOWNSTREAM_OF_NMDARS | 0.028 |
| 765 | REACTOME_DNA_DAMAGE_RECOGNITION_IN_GG_NER | 0.028 |
| 766 | REACTOME_RHO_GTPASES_ACTIVATE_RHOTEKIN_AND_RHOPHILINS | 0.027 |
| 767 | REACTOME_INTERCONVERSION_OF_NUCLEOTIDE_DI_AND_TRIPHOSPHATES | 0.027 |
| 768 | REACTOME_TP53_REGULATES_TRANSCRIPTION_OF_SEVERAL_ADDITIONAL_CELL_DEATH_GENES_WHOSE_SPECIFIC_ROLES_IN_P53_DEPENDENT_APOPTOSIS_REMAIN_UNCERTAIN | 0.027 |
| 769 | REACTOME_HIGHLY_CALCIUM_PERMEABLE_NICOTINIC_ACETYLCHOLINE_RECEPTORS | 0.027 |
| 770 | REACTOME_RHO_GTPASES_ACTIVATE_CIT | 0.027 |
| 771 | REACTOME_MRNA_SPLICING_MINOR_PATHWAY | 0.027 |
| 772 | REACTOME_NEUROTOXICITY_OF_CLOSTRIDIUM_TOXINS | 0.027 |
| 773 | REACTOME_ACTIVATION_OF_THE_TFAP2_AP_2_FAMILY_OF_TRANSCRIPTION_FACTORS | 0.027 |
| 774 | REACTOME_SYNAPTIC_ADHESION_LIKE_MOLECULES | 0.027 |
| 775 | REACTOME_SYNTHESIS_OF_IP3_AND_IP4_IN_THE_CYTOSOL | 0.027 |
| 776 | REACTOME_ACYL_CHAIN_REMODELING_OF_DAG_AND_TAG | 0.027 |
| 777 | REACTOME_BETA_DEFENSINS | 0.027 |
| 778 | REACTOME_GERM_LAYER_FORMATION_AT_GASTRULATION | 0.026 |
| 779 | REACTOME_ALPHA_LINOLENIC_OMEGA3_AND_LINOLEIC_OMEGA6_ACID_METABOLISM | 0.026 |
| 780 | REACTOME_HEME_BIOSYNTHESIS | 0.026 |
| 781 | REACTOME_MET_ACTIVATES_PTPN11 | 0.026 |
| 782 | REACTOME_ARACHIDONATE_PRODUCTION_FROM_DAG | 0.026 |
| 783 | REACTOME_APOBEC3G_MEDIATED_RESISTANCE_TO_HIV_1_INFECTION | 0.026 |
| 784 | REACTOME_SCF_BETA_TRCP_MEDIATED_DEGRADATION_OF_EMI1 | 0.025 |
| 785 | REACTOME_ACTIVATION_OF_KAINATE_RECEPTORS_UPON_GLUTAMATE_BINDING | 0.025 |
| 786 | REACTOME_PKA_ACTIVATION_IN_GLUCAGON_SIGNALLING | 0.025 |
| 787 | REACTOME_FIBRONECTIN_MATRIX_FORMATION | 0.025 |
| 788 | REACTOME_MITOCHONDRIAL_IRON_SULFUR_CLUSTER_BIOGENESIS | 0.025 |
| 789 | REACTOME_INOSITOL_PHOSPHATE_METABOLISM | 0.024 |
| 790 | REACTOME_BUDDING_AND_MATURATION_OF_HIV_VIRION | 0.024 |
| 791 | REACTOME_DIGESTION | 0.024 |
| 792 | REACTOME_SPERM_MOTILITY_AND_TAXES | 0.024 |
| 793 | REACTOME_ADP_SIGNALLING_THROUGH_P2Y_PURINOCEPTOR_12 | 0.024 |
| 794 | REACTOME_FOXO_MEDIATED_TRANSCRIPTION_OF_CELL_DEATH_GENES | 0.024 |
| 795 | REACTOME_IRE1ALPHA_ACTIVATES_CHAPERONES | 0.024 |
| 796 | REACTOME_RUNX2_REGULATES_GENES_INVOLVED_IN_CELL_MIGRATION | 0.024 |
| 797 | REACTOME_TP53_REGULATES_TRANSCRIPTION_OF_GENES_INVOLVED_IN_G1_CELL_CYCLE_ARREST | 0.024 |
| 798 | REACTOME_GRB2_SOS_PROVIDES_LINKAGE_TO_MAPK_SIGNALING_FOR_INTEGRINS | 0.023 |
| 799 | REACTOME_METABOLISM_OF_AMINE_DERIVED_HORMONES | 0.023 |
| 800 | REACTOME_CYTOSOLIC_SULFONATION_OF_SMALL_MOLECULES | 0.023 |
| 801 | REACTOME_LINOLEIC_ACID_LA_METABOLISM | 0.023 |
| 802 | REACTOME_MET_RECEPTOR_RECYCLING | 0.023 |
| 803 | REACTOME_SIGNALING_BY_MST1 | 0.022 |
| 804 | REACTOME_NTRK2_ACTIVATES_RAC1 | 0.022 |
| 805 | REACTOME_LRR_FLII_INTERACTING_PROTEIN_1_LRRFIP1_ACTIVATES_TYPE_I_IFN_PRODUCTION | 0.022 |
| 806 | REACTOME_RUNX3_REGULATES_P14_ARF | 0.022 |
| 807 | REACTOME_G1_S_SPECIFIC_TRANSCRIPTION | 0.022 |
| 808 | REACTOME_COBALAMIN_CBL_VITAMIN_B12_TRANSPORT_AND_METABOLISM | 0.022 |
| 809 | REACTOME_ENDOSOMAL_VACUOLAR_PATHWAY | 0.022 |
| 810 | REACTOME_G_PROTEIN_ACTIVATION | 0.022 |
| 811 | REACTOME_ACTIVATED_NTRK3_SIGNALS_THROUGH_RAS | 0.021 |
| 812 | REACTOME_SYNTHESIS_OF_IP2_IP_AND_INS_IN_THE_CYTOSOL | 0.021 |
| 813 | REACTOME_NUCLEAR_PORE_COMPLEX_NPC_DISASSEMBLY | 0.021 |
| 814 | REACTOME_RUNX3_REGULATES_WNT_SIGNALING | 0.021 |
| 815 | REACTOME_MET_ACTIVATES_RAS_SIGNALING | 0.021 |
| 816 | REACTOME_RUNX3_REGULATES_BCL2L11_BIM_TRANSCRIPTION | 0.021 |
| 817 | REACTOME_MRNA_CAPPING | 0.021 |
| 818 | REACTOME_P130CAS_LINKAGE_TO_MAPK_SIGNALING_FOR_INTEGRINS | 0.021 |
| 819 | REACTOME_REGULATION_OF_COMMISSURAL_AXON_PATHFINDING_BY_SLIT_AND_ROBO | 0.020 |
| 820 | REACTOME_VIRAL_MESSENGER_RNA_SYNTHESIS | 0.020 |
| 821 | REACTOME_OTHER_SEMAPHORIN_INTERACTIONS | 0.020 |
| 822 | REACTOME_RUNX1_AND_FOXP3_CONTROL_THE_DEVELOPMENT_OF_REGULATORY_T_LYMPHOCYTES_TREGS | 0.020 |
| 823 | REACTOME_ATTENUATION_PHASE | 0.020 |
| 824 | REACTOME_ABACAVIR_METABOLISM | 0.020 |
| 825 | REACTOME_ABC_TRANSPORTERS_IN_LIPID_HOMEOSTASIS | 0.020 |
| 826 | REACTOME_ACTIVATION_OF_PUMA_AND_TRANSLOCATION_TO_MITOCHONDRIA | 0.019 |
| 827 | REACTOME_TRAIL_SIGNALING | 0.019 |
| 828 | REACTOME_SUMOYLATION_OF_DNA_METHYLATION_PROTEINS | 0.019 |
| 829 | REACTOME_DIGESTION_OF_DIETARY_CARBOHYDRATE | 0.019 |
| 830 | REACTOME_DEFECTIVE_RIPK1_MEDIATED_REGULATED_NECROSIS | 0.019 |
| 831 | REACTOME_MET_RECEPTOR_ACTIVATION | 0.019 |
| 832 | REACTOME_DOPAMINE_NEUROTRANSMITTER_RELEASE_CYCLE | 0.019 |
| 833 | REACTOME_LOSS_OF_FUNCTION_OF_MECP2_IN_RETT_SYNDROME | 0.019 |
| 834 | REACTOME_REGULATION_OF_GENE_EXPRESSION_IN_LATE_STAGE_BRANCHING_MORPHOGENESIS_PANCREATIC_BUD_PRECURSOR_CELLS | 0.019 |
| 835 | REACTOME_PRESYNAPTIC_NICOTINIC_ACETYLCHOLINE_RECEPTORS | 0.019 |
| 836 | REACTOME_SYNTHESIS_OF_VERY_LONG_CHAIN_FATTY_ACYL_COAS | 0.018 |
| 837 | REACTOME_REGULATION_OF_GLYCOLYSIS_BY_FRUCTOSE_2_6_BISPHOSPHATE_METABOLISM | 0.018 |
| 838 | REACTOME_CS_DS_DEGRADATION | 0.018 |
| 839 | REACTOME_MITOCHONDRIAL_CALCIUM_ION_TRANSPORT | 0.018 |
| 840 | REACTOME_N_GLYCAN_TRIMMING_IN_THE_ER_AND_CALNEXIN_CALRETICULIN_CYCLE | 0.018 |
| 841 | REACTOME_PROTEIN_REPAIR | 0.018 |
| 842 | REACTOME_SYNTHESIS_OF_PG | 0.018 |
| 843 | REACTOME_TELOMERE_EXTENSION_BY_TELOMERASE | 0.018 |
| 844 | REACTOME_THYROXINE_BIOSYNTHESIS | 0.018 |
| 845 | REACTOME_FOLDING_OF_ACTIN_BY_CCT_TRIC | 0.017 |
| 846 | REACTOME_ACTIVATED_NTRK2_SIGNALS_THROUGH_CDK5 | 0.017 |
| 847 | REACTOME_TRANSLESION_SYNTHESIS_BY_Y_FAMILY_DNA_POLYMERASES_BYPASSES_LESIONS_ON_DNA_TEMPLATE | 0.017 |
| 848 | REACTOME_TIGHT_JUNCTION_INTERACTIONS | 0.017 |
| 849 | REACTOME_PENTOSE_PHOSPHATE_PATHWAY | 0.017 |
| 850 | REACTOME_ENOS_ACTIVATION | 0.017 |
| 851 | REACTOME_DEFECTIVE_CHST6_CAUSES_MCDC1 | 0.017 |
| 852 | REACTOME_RECOGNITION_OF_DNA_DAMAGE_BY_PCNA_CONTAINING_REPLICATION_COMPLEX | 0.017 |
| 853 | REACTOME_ACTIVATION_OF_THE_PRE_REPLICATIVE_COMPLEX | 0.017 |
| 854 | REACTOME_HDR_THROUGH_MMEJ_ALT_NHEJ | 0.017 |
| 855 | REACTOME_TRANSCRIPTION_OF_E2F_TARGETS_UNDER_NEGATIVE_CONTROL_BY_P107_RBL1_AND_P130_RBL2_IN_COMPLEX_WITH_HDAC1 | 0.017 |
| 856 | REACTOME_METAL_SEQUESTRATION_BY_ANTIMICROBIAL_PROTEINS | 0.017 |
| 857 | REACTOME_NOSTRIN_MEDIATED_ENOS_TRAFFICKING | 0.017 |
| 858 | REACTOME_LISTERIA_MONOCYTOGENES_ENTRY_INTO_HOST_CELLS | 0.016 |
| 859 | REACTOME_RHO_GTPASES_ACTIVATE_IQGAPS | 0.016 |
| 860 | REACTOME_INLB_MEDIATED_ENTRY_OF_LISTERIA_MONOCYTOGENES_INTO_HOST_CELL | 0.016 |
| 861 | REACTOME_DOWNREGULATION_OF_ERBB4_SIGNALING | 0.016 |
| 862 | REACTOME_ASYMMETRIC_LOCALIZATION_OF_PCP_PROTEINS | 0.016 |
| 863 | REACTOME_VITAMIN_C_ASCORBATE_METABOLISM | 0.016 |
| 864 | REACTOME_DISEASES_OF_CARBOHYDRATE_METABOLISM | 0.016 |
| 865 | REACTOME_SENSORY_PERCEPTION_OF_SALTY_TASTE | 0.016 |
| 866 | REACTOME_PROCESSING_AND_ACTIVATION_OF_SUMO | 0.016 |
| 867 | REACTOME_LONG_TERM_POTENTIATION | 0.016 |
| 868 | REACTOME_MASTL_FACILITATES_MITOTIC_PROGRESSION | 0.016 |
| 869 | REACTOME_VASOPRESSIN_REGULATES_RENAL_WATER_HOMEOSTASIS_VIA_AQUAPORINS | 0.016 |
| 870 | REACTOME_CHONDROITIN_SULFATE_DERMATAN_SULFATE_METABOLISM | 0.016 |
| 871 | REACTOME_ABORTIVE_ELONGATION_OF_HIV_1_TRANSCRIPT_IN_THE_ABSENCE_OF_TAT | 0.016 |
| 872 | REACTOME_PHENYLALANINE_METABOLISM | 0.015 |
| 873 | REACTOME_METALLOPROTEASE_DUBS | 0.015 |
| 874 | REACTOME_BIOTIN_TRANSPORT_AND_METABOLISM | 0.015 |
| 875 | REACTOME_REGULATION_OF_RUNX1_EXPRESSION_AND_ACTIVITY | 0.015 |
| 876 | REACTOME_POST_CHAPERONIN_TUBULIN_FOLDING_PATHWAY | 0.014 |
| 877 | REACTOME_GLYOXYLATE_METABOLISM_AND_GLYCINE_DEGRADATION | 0.014 |
| 878 | REACTOME_SEROTONIN_NEUROTRANSMITTER_RELEASE_CYCLE | 0.014 |
| 879 | REACTOME_ASSEMBLY_AND_CELL_SURFACE_PRESENTATION_OF_NMDA_RECEPTORS | 0.014 |
| 880 | REACTOME_FORMATION_OF_TUBULIN_FOLDING_INTERMEDIATES_BY_CCT_TRIC | 0.014 |
| 881 | REACTOME_FBXW7_MUTANTS_AND_NOTCH1_IN_CANCER | 0.014 |
| 882 | REACTOME_RHOBTB2_GTPASE_CYCLE | 0.014 |
| 883 | REACTOME_GLYCOGEN_METABOLISM | 0.014 |
| 884 | REACTOME_DEFECTIVE_B4GALT1_CAUSES_B4GALT1_CDG_CDG_2D | 0.014 |
| 885 | REACTOME_IRAK2_MEDIATED_ACTIVATION_OF_TAK1_COMPLEX | 0.014 |
| 886 | REACTOME_NOTCH3_INTRACELLULAR_DOMAIN_REGULATES_TRANSCRIPTION | 0.014 |
| 887 | REACTOME_GLUCONEOGENESIS | 0.014 |
| 888 | REACTOME_TLR3_MEDIATED_TICAM1_DEPENDENT_PROGRAMMED_CELL_DEATH | 0.013 |
| 889 | REACTOME_RHO_GTPASES_ACTIVATE_KTN1 | 0.013 |
| 890 | REACTOME_SCF_SKP2_MEDIATED_DEGRADATION_OF_P27_P21 | 0.013 |
| 891 | REACTOME_ADENYLATE_CYCLASE_INHIBITORY_PATHWAY | 0.013 |
| 892 | REACTOME_METABOLISM_OF_FOLATE_AND_PTERINES | 0.013 |
| 893 | REACTOME_ACTIVATION_OF_RAC1 | 0.013 |
| 894 | REACTOME_PURINE_SALVAGE | 0.013 |
| 895 | REACTOME_AMINO_ACID_TRANSPORT_ACROSS_THE_PLASMA_MEMBRANE | 0.013 |
| 896 | REACTOME_SUMO_IS_PROTEOLYTICALLY_PROCESSED | 0.013 |
| 897 | REACTOME_FORMATION_OF_THE_EARLY_ELONGATION_COMPLEX | 0.012 |
| 898 | REACTOME_KERATAN_SULFATE_BIOSYNTHESIS | 0.012 |
| 899 | REACTOME_G2_M_DNA_REPLICATION_CHECKPOINT | 0.012 |
| 900 | REACTOME_CARGO_CONCENTRATION_IN_THE_ER | 0.012 |
| 901 | REACTOME_PKA_MEDIATED_PHOSPHORYLATION_OF_CREB | 0.012 |
| 902 | REACTOME_RUNX1_REGULATES_EXPRESSION_OF_COMPONENTS_OF_TIGHT_JUNCTIONS | 0.012 |
| 903 | REACTOME_PHOSPHATE_BOND_HYDROLYSIS_BY_NTPDASE_PROTEINS | 0.012 |
| 904 | REACTOME_MATURATION_OF_SARSCOV2_SPIKE_PROTEIN | 0.012 |
| 905 | REACTOME_HIV_TRANSCRIPTION_ELONGATION | 0.012 |
| 906 | REACTOME_GLUTAMATE_NEUROTRANSMITTER_RELEASE_CYCLE | 0.012 |
| 907 | REACTOME_PLATELET_CALCIUM_HOMEOSTASIS | 0.012 |
| 908 | REACTOME_PTK6_REGULATES_CELL_CYCLE | 0.012 |
| 909 | REACTOME_PI5P_REGULATES_TP53_ACETYLATION | 0.011 |
| 910 | REACTOME_PEPTIDE_HORMONE_BIOSYNTHESIS | 0.011 |
| 911 | REACTOME_ADENYLATE_CYCLASE_ACTIVATING_PATHWAY | 0.011 |
| 912 | REACTOME_MET_PROMOTES_CELL_MOTILITY | 0.011 |
| 913 | REACTOME_TICAM1_TRAF6_DEPENDENT_INDUCTION_OF_TAK1_COMPLEX | 0.011 |
| 914 | REACTOME_CARBOXYTERMINAL_POST_TRANSLATIONAL_MODIFICATIONS_OF_TUBULIN | 0.011 |
| 915 | REACTOME_HIV_ELONGATION_ARREST_AND_RECOVERY | 0.011 |
| 916 | REACTOME_DRUG_MEDIATED_INHIBITION_OF_CDK4_CDK6_ACTIVITY | 0.011 |
| 917 | REACTOME_SODIUM_PROTON_EXCHANGERS | 0.011 |
| 918 | REACTOME_HIGHLY_SODIUM_PERMEABLE_POSTSYNAPTIC_ACETYLCHOLINE_NICOTINIC_RECEPTORS | 0.011 |
| 919 | REACTOME_INTESTINAL_ABSORPTION | 0.010 |
| 920 | REACTOME_UNBLOCKING_OF_NMDA_RECEPTORS_GLUTAMATE_BINDING_AND_ACTIVATION | 0.010 |
| 921 | REACTOME_PROTEIN_METHYLATION | 0.010 |
| 922 | REACTOME_MISMATCH_REPAIR | 0.010 |
| 923 | REACTOME_E2F_ENABLED_INHIBITION_OF_PRE_REPLICATION_COMPLEX_FORMATION | 0.010 |
| 924 | REACTOME_FORMATION_OF_INCISION_COMPLEX_IN_GG_NER | 0.010 |
| 925 | REACTOME_TERMINATION_OF_TRANSLESION_DNA_SYNTHESIS | 0.010 |
| 926 | REACTOME_POLB_DEPENDENT_LONG_PATCH_BASE_EXCISION_REPAIR | 0.010 |
| 927 | REACTOME_PTK6_REGULATES_PROTEINS_INVOLVED_IN_RNA_PROCESSING | 0.010 |
| 928 | REACTOME_METABOLISM_OF_NITRIC_OXIDE_NOS3_ACTIVATION_AND_REGULATION | 0.010 |
| 929 | REACTOME_ATF6_ATF6_ALPHA_ACTIVATES_CHAPERONES | 0.009 |
| 930 | REACTOME_DUAL_INCISION_IN_GG_NER | 0.009 |
| 931 | REACTOME_PASSIVE_TRANSPORT_BY_AQUAPORINS | 0.009 |
| 932 | REACTOME_ACETYLCHOLINE_NEUROTRANSMITTER_RELEASE_CYCLE | 0.009 |
| 933 | REACTOME_ABERRANT_REGULATION_OF_MITOTIC_G1_S_TRANSITION_IN_CANCER_DUE_TO_RB1_DEFECTS | 0.009 |
| 934 | REACTOME_MET_ACTIVATES_RAP1_AND_RAC1 | 0.009 |
| 935 | REACTOME_FORMATION_OF_RNA_POL_II_ELONGATION_COMPLEX | 0.009 |
| 936 | REACTOME_RAS_ACTIVATION_UPON_CA2_INFLUX_THROUGH_NMDA_RECEPTOR | 0.009 |
| 937 | REACTOME_MATURATION_OF_SARSCOV1_SPIKE_PROTEIN | 0.009 |
| 938 | REACTOME_POLYMERASE_SWITCHING_ON_THE_C_STRAND_OF_THE_TELOMERE | 0.009 |
| 939 | REACTOME_GLUTATHIONE_SYNTHESIS_AND_RECYCLING | 0.009 |
| 940 | REACTOME_RHOBTB3_ATPASE_CYCLE | 0.008 |
| 941 | REACTOME_DISPLACEMENT_OF_DNA_GLYCOSYLASE_BY_APEX1 | 0.008 |
| 942 | REACTOME_MUCOPOLYSACCHARIDOSES | 0.008 |
| 943 | REACTOME_CREB1_PHOSPHORYLATION_THROUGH_THE_ACTIVATION_OF_ADENYLATE_CYCLASE | 0.008 |
| 944 | REACTOME_RHO_GTPASES_ACTIVATE_PAKS | 0.008 |
| 945 | REACTOME_DISEASES_OF_MITOTIC_CELL_CYCLE | 0.008 |
| 946 | REACTOME_SEROTONIN_AND_MELATONIN_BIOSYNTHESIS | 0.008 |
| 947 | REACTOME_GLYCOGEN_STORAGE_DISEASES | 0.007 |
| 948 | REACTOME_DUAL_INCISION_IN_TC_NER | 0.007 |
| 949 | REACTOME_G2_PHASE | 0.007 |
| 950 | REACTOME_NECTIN_NECL_TRANS_HETERODIMERIZATION | 0.007 |
| 951 | REACTOME_INTRAFLAGELLAR_TRANSPORT | 0.007 |
| 952 | REACTOME_DEFECTIVE_ST3GAL3_CAUSES_MCT12_AND_EIEE15 | 0.007 |
| 953 | REACTOME_RESOLUTION_OF_ABASIC_SITES_AP_SITES | 0.007 |
| 954 | REACTOME_ANTIGEN_PRESENTATION_FOLDING_ASSEMBLY_AND_PEPTIDE_LOADING_OF_CLASS_I_MHC | 0.007 |
| 955 | REACTOME_PHENYLALANINE_AND_TYROSINE_METABOLISM | 0.007 |
| 956 | REACTOME_LAGGING_STRAND_SYNTHESIS | 0.006 |
| 957 | REACTOME_MITOCHONDRIAL_FATTY_ACID_BETA_OXIDATION | 0.006 |
| 958 | REACTOME_TETRAHYDROBIOPTERIN_BH4_SYNTHESIS_RECYCLING_SALVAGE_AND_REGULATION | 0.006 |
| 959 | REACTOME_E2F_MEDIATED_REGULATION_OF_DNA_REPLICATION | 0.006 |
| 960 | REACTOME_ALPHA_PROTEIN_KINASE_1_SIGNALING_PATHWAY | 0.006 |
| 961 | REACTOME_DEFECTS_IN_BIOTIN_BTN_METABOLISM | 0.006 |
| 962 | REACTOME_RECYCLING_OF_EIF2_GDP | 0.006 |
| 963 | REACTOME_SYNTHESIS_OF_ACTIVE_UBIQUITIN_ROLES_OF_E1_AND_E2_ENZYMES | 0.006 |
| 964 | REACTOME_GLYCOGEN_BREAKDOWN_GLYCOGENOLYSIS | 0.006 |
| 965 | REACTOME_CREB1_PHOSPHORYLATION_THROUGH_THE_ACTIVATION_OF_CAMKII_CAMKK_CAMKIV_CASCASDE | 0.006 |
| 966 | REACTOME_INACTIVATION_OF_CDC42_AND_RAC1 | 0.006 |
| 967 | REACTOME_CROSS_PRESENTATION_OF_PARTICULATE_EXOGENOUS_ANTIGENS_PHAGOSOMES | 0.005 |
| 968 | REACTOME_DNA_REPLICATION_INITIATION | 0.005 |
| 969 | REACTOME_APC_C_CDC20_MEDIATED_DEGRADATION_OF_CYCLIN_B | 0.005 |
| 970 | REACTOME_ACTIVATION_OF_RAC1_DOWNSTREAM_OF_NMDARS | 0.005 |
| 971 | REACTOME_TELOMERE_C_STRAND_LAGGING_STRAND_SYNTHESIS | 0.005 |
| 972 | REACTOME_LOSS_OF_MECP2_BINDING_ABILITY_TO_THE_NCOR_SMRT_COMPLEX | 0.005 |
| 973 | REACTOME_NEGATIVE_REGULATION_OF_NMDA_RECEPTOR_MEDIATED_NEURONAL_TRANSMISSION | 0.005 |
| 974 | REACTOME_CREATINE_METABOLISM | 0.004 |
| 975 | REACTOME_CALNEXIN_CALRETICULIN_CYCLE | 0.004 |
| 976 | REACTOME_TELOMERE_C_STRAND_SYNTHESIS_INITIATION | 0.004 |
| 977 | REACTOME_TRISTETRAPROLIN_TTP_ZFP36_BINDS_AND_DESTABILIZES_MRNA | 0.004 |
| 978 | REACTOME_REDUCTION_OF_CYTOSOLIC_CA_LEVELS | 0.004 |
| 979 | REACTOME_CAMK_IV_MEDIATED_PHOSPHORYLATION_OF_CREB | 0.004 |
| 980 | REACTOME_SYNTHESIS_OF_DIPHTHAMIDE_EEF2 | 0.004 |
| 981 | REACTOME_INHIBITION_OF_REPLICATION_INITIATION_OF_DAMAGED_DNA_BY_RB1_E2F1 | 0.004 |
| 982 | REACTOME_DNA_STRAND_ELONGATION | 0.004 |
| 983 | REACTOME_PROCESSIVE_SYNTHESIS_ON_THE_LAGGING_STRAND | 0.004 |
| 984 | REACTOME_GAP_FILLING_DNA_REPAIR_SYNTHESIS_AND_LIGATION_IN_GG_NER | 0.004 |
| 985 | REACTOME_RESOLUTION_OF_AP_SITES_VIA_THE_MULTIPLE_NUCLEOTIDE_PATCH_REPLACEMENT_PATHWAY | 0.004 |
| 986 | REACTOME_SEMA3A_PLEXIN_REPULSION_SIGNALING_BY_INHIBITING_INTEGRIN_ADHESION | 0.003 |
| 987 | REACTOME_PCNA_DEPENDENT_LONG_PATCH_BASE_EXCISION_REPAIR | 0.003 |
| 988 | REACTOME_DEFECTS_IN_VITAMIN_AND_COFACTOR_METABOLISM | 0.003 |
| 989 | REACTOME_FORMATION_OF_XYLULOSE_5_PHOSPHATE | 0.003 |
| 990 | REACTOME_APEX1_INDEPENDENT_RESOLUTION_OF_AP_SITES_VIA_THE_SINGLE_NUCLEOTIDE_REPLACEMENT_PATHWAY | 0.003 |
| 991 | REACTOME_SODIUM_CALCIUM_EXCHANGERS | 0.003 |
| 992 | REACTOME_POLYMERASE_SWITCHING | 0.003 |
| 993 | REACTOME_RNA_POLYMERASE_I_TRANSCRIPTION_TERMINATION | 0.003 |
| 994 | REACTOME_RESPONSE_OF_EIF2AK1_HRI_TO_HEME_DEFICIENCY | 0.003 |
| 995 | REACTOME_PROPIONYL_COA_CATABOLISM | 0.003 |
| 996 | REACTOME_RUNX3_REGULATES_YAP1_MEDIATED_TRANSCRIPTION | 0.003 |
| 997 | REACTOME_REELIN_SIGNALLING_PATHWAY | 0.002 |
| 998 | REACTOME_PP2A_MEDIATED_DEPHOSPHORYLATION_OF_KEY_METABOLIC_FACTORS | 0.002 |
| 999 | REACTOME_PROCESSIVE_SYNTHESIS_ON_THE_C_STRAND_OF_THE_TELOMERE | 0.002 |
| 1000 | REACTOME_UPTAKE_OF_DIETARY_COBALAMINS_INTO_ENTEROCYTES | 0.001 |
| 1001 | REACTOME_CYTOSOLIC_IRON_SULFUR_CLUSTER_ASSEMBLY | 0.001 |

**Table S2. Antibody suppliers, catalog numbers and molecular weight.**

| Antibodies | Application | Supplier | Catalog number | Molecular weight (kDa) |
| --- | --- | --- | --- | --- |
| β-actin | WB | CST | 4967 | 43 |
| STING | WB/Co-IP/IF | CST | 13647 | 39 |
| p-STING (S365) | WB | CST | 72971 | 39 |
| p-TBK1 (S172) | WB/IF | CST | 5483 | 84 |
| TBK1 | WB/Co-IP | Abclonal | A3458 | 84 |
| IRF3 | WB/Co-IP | Abclonal | A2172 | 51 |
| p-IRF3 (S396) | WB/IF | CST | 4947 | 51 |
| GPX4 | WB | Abcam | ab125066 | 22 |
| Tomm20 | IF | Abclonal | A19403 | / |
| TFAM | IF | Abclonal | A3173 | / |
| VDAC1 | WB | Proteintech | 66345-1-Ig | 35 |
| CD68 | IF | CST | 76437 | / |
| Vinculin | WB | Abclonal | A2752 | 124 |
| GAPDH | WB | Abclonal | A19056 | 36 |
| NLRP3 | WB/IF | Wanlei bio | WL02635 | 117 |
| CASP1 | WB/IF | Abmart | M025280 | 50, 20 |
| IL-18 | WB | CST | 57058 | 22, 17 |
| GSDMD | WB | CST | 39754 | 53, 30 |
| ACSL4 | WB | CST | 38493 | 80 |
| FTH1 | WB | CST | 4393 | 21 |
| cGAS | WB/IF | Abclonal | A8335 | 62 |
| iNOS | WB | CST | 13120 | 130 |
| CD86 | WB/IF | CST | 19589 | 86 |
| MUC2 | WB/IF | Abcam | ab272692 | 180 |
| ZO-1 | IF | Proteintech | 21773-1-AP | / |
| Claudin-1 | WB/IF | Abcam | ab307692 | 18 |
| Occludin | WB/IF | Abcam | ab216327 | 65 |
| TFF3 | IF | Abcam | ab300427 | / |
| E-cadherin | IF | CST | 3195 | / |
| Ki67 | IF | CST | 34330 | / |
| F4/80 | IF | CST | 70076 | / |
| Anti-Mouse IgG | IF | Abcam | ab150114 | / |
| Anti-Rabbit IgG | IF | Abcam | ab150077 | / |
| APC/Cyanine7 anti-mouse CD45 Antibody | FC | Biolegend | 103116 | / |
| FITC anti-mouse/human CD11b Antibody | FC | Biolegend | 101206 | / |
| PerCP/Cyanine5.5 anti-mouse F4/80 Antibody | FC | Biolegend | 123128 | / |
| APC anti-mouse CD86 Antibody | FC | Biolegend | 105012 | / |
| Alexa Fluor® 700 anti-mouse CD206 (MMR) Antibody | FC | Biolegend | 141734 | / |

**Table S3. Clinical characteristics of patients with UC and control donors.**

|  | Controls | UC |
| --- | --- | --- |
| Number | 10 | 21 |
| Male, n (%) | 6 (60%) | 15 (71.4%) |
| Mean age, years (range) | 35.17 (23-55) | 31.36 (19-58) |
| Origin of samples, n (%)  Ileum  Rectum  Ileocecal  Colon | 0  0  0  10 (100%) | 2 (9.5%)  2 (9.5%)  3 (14.3%)  14 (66.7%) |
| Current therapy, n (%)  5-aminosalicylates  Immunosuppressants  Biologics  Nutritional therapy | - | 5 (23.8%)  2 (9.5%)  11 (52.4%)  3 (14.3%) |
| Disease location, n (%)  E1  E2  E3 | - | 4 (19.0%)  14 (66.7%)  3 (14.3%) |

Note: E1: Proctitis; E2: Left-sided colitis; E3: Extensive colitis.

**Table S4. Primer sequences for real-time PCR.**

|  | Gene | Forward sequence | Reverse sequence |
| --- | --- | --- | --- |
| Mouse | *IL-1β* | GCAACTGTTCCTGAACTCAACT | ATCTTTTGGGGTCCGTCAACT |
|  | *IL-6* | TAGTCCTTCCTACCCCAATTTCC | TTGGTCCTTAGCCACTCCTTC |
|  | *TNF-α* | CCCTCACACTCAGATCATCTTCT | GCTACGACGTGGGCTACAG |
|  | *IL-23* | CATGCTAGCCTGGAACGCACAT | ACTGGCTGTTGTCCTTGAGTCC |
|  | *ACSL4* | CTCACCATTATATTGCTGCCTGT | TCTCTTTGCCATAGCGTTTTTCT |
|  | *GPX4* | GATGGAGCCCATTCCTGAACC | CCCTGTACTTATCCAGGCAGA |
|  | *IFNB* | CAGCTCCAAGAAAGGACGAAC | GGCAGTGTAACTCTTCTGCAT |
|  | *CCL5* | GCTGCTTTGCCTACCTCTCC | TCGAGTGACAAACACGACTGC |
|  | *CXCL10* | CCAAGTGCTGCCGTCATTTTC | GGCTCGCAGGGATGATTTCAA |
|  | *IFIT1* | CTGAGATGTCACTTCACATGGAA | GTGCATCCCCAATGGGTTCT |
|  | *ISG15* | GGTGTCCGTGACTAACTCCAT | TGGAAAGGGTAAGACCGTCCT |
|  | *iNOS* | GTTCTCAGCCCAACAATACAAGA | GTGGACGGGTCGATGTCAC |
|  | *18S rRNA* | AGGGGAGAGCGGGTAAGAGA | GGACAGGACTAGGCGGAACA |
|  | *CD86* | TGTTTCCGTGGAGACGCAAG | TTGAGCCTTTGTAAATGGGCA |
|  | *mt-Nd1* | TATCTCAACCCTAGCAGAAA | TAACGCGAATGGGCCGGCTG |
|  | *mt-Nd2* | AGGGGCATGAGGAGGACTTA | TTGAGTAGAGTGAGGGATGGGT |
|  | *D-loop* | AATCTACCATCCTCCGTGAAACC | TCAGTTTAGCTACCCCCAAGTTTAA |
|  | *mt-Cytb* | GCAACCTTGACCCGATTCTTCGC | TGAACGATTGCTAGGGCCGCG |
| Human | *IFNB* | GCTTGGATTCCTACAAAGAAGCA | ATAGATGGTCAATGCGGCGTC |
|  | *CCL5* | CCAGCAGTCGTCTTTGTCAC | CTCTGGGTTGGCACACACTT |
|  | *CXCL10* | GTGGCATTCAAGGAGTACCTC | TGATGGCCTTCGATTCTGGATT |
|  | *IFIT1* | AGAAGCAGGCAATCACAGAAAA | CTGAAACCGACCATAGTGGAAAT |
|  | *ISG15* | TGGACAAATGCGACGAACCTC | TCAGCCGTACCTCGTAGGTG |
|  | *MT-ND1* | GGCTATATACAACTACGCAAAGGC | GGTAGATGTGGCGGGTTTTAGG |
|  | *MT-ND2* | CACACTCATCACAGCGCTAA | GGATTATGGATGCGGTTGCT |
|  | *D-LOOP* | CCTCCCACTCCCATACTACTAA | GTGATGTGAGCCCGTCTAAACA |
|  | *MT-CYB* | GCGTCCTTGCCCTATTACTATC | CTTACTGGTTGTCCTCCGATTC |
